# Development of Piperazine- and Oxazine-Linked Pyrimidines as p65 Subunit Binders of NF–κB in Human Breast Cancer Cells

**DOI:** 10.3390/biomedicines11102716

**Published:** 2023-10-06

**Authors:** Akshay Ravish, Bhanuprakash C. Narasimhachar, Zhang Xi, Divakar Vishwanath, Arunkumar Mohan, Santosh L. Gaonkar, Paduvalahippe Gowdegowda Chandrashekara, Kwang Seok Ahn, Vijay Pandey, Peter E. Lobie, Basappa Basappa

**Affiliations:** 1 Laboratory of Chemical Biology, Department of Studies in Organic Chemistry, University of Mysore, Mysore 570006, Karnataka, India; akshayrv533@gmail.com (A.R.); divakardivi166@gmail.com (D.V.); arunmysore3@gmail.com (A.M.); 2 Department of Chemistry, Yuvaraja’s College, University of Mysore, Mysuru 570005, Karnataka, India; bhanuprakashcn28@gmail.com (B.C.N.); pgc2031@gmail.com (P.G.C.); 3 Shenzhen Bay Laboratory, Shenzhen 518055, China; zhangxi@szbl.ac.cn; 4 Department of Chemistry, Manipal Institute of Technology, Manipal Academy of Higher Education, Manipal 576104, Karnataka, India; sl.gaonkar@manipal.edu; 5 Department of Science in Korean Medicine, Kyung Hee University, 24 Kyungheedaero, Dongdaemungu, Seoul 02447, Republic of Korea; ksahn@khu.ac.kr; 6 Tsinghua Berkeley Shenzhen Institute, Tsinghua Shenzhen International Graduate School, Tsinghua University, Shenzhen 518055, China; vijay.pandey@sz.tsinghua.edu.cn; 7 Institute of Biopharmaceutical and Health Engineering, Tsinghua Shenzhen International Graduate School, Tsinghua University, Shenzhen 518055, China

**Keywords:** oxazines, piperazines, pyrimidines, NF–κB, Alamar Blue assay, molecular docking, apoptosis assay, western blot

## Abstract

Nuclear factor kappa B (NF–κB) is a potential therapeutic target in breast cancer. In the current study, a new class of oxazine– and piperazine–linked pyrimidines was developed as inhibitors of NF–κB, overcoming the complexity of the oxazine structure found in nature and enabling synthesis under laboratory conditions. Among the series of synthesized and tested oxazine–pyrimidine and piperazine–pyrimidine derivatives, compounds **3a** and **5b** inhibited breast cancer cell (MCF–7) viability with an IC_50_ value of 9.17 and 6.29 µM, respectively. *In silico* docking studies showed that the pyrimidine ring of **3a** and the 4–methoxybenzyl thiol group of **5b** could strongly bind the p65 subunit of NF–κB, with the binding energies −9.32 and −7.32 kcal mol^−1^. Furthermore, compounds **3a** and **5b** inhibited NF–κB in MCF–7 breast cancer cells. In conclusion, we herein report newer structures that target NF–κB in BC cells.

## 1. Introduction

Breast cancer has become the world’s second-leading cause of cancer-related death (lung cancer being first), accounting for about 13.7% of all cancer-related fatalities [1,2]. Nuclear factor kappa B (NF–κB) signaling has been extensively studied for over three decades since its discovery by Sen et al. [3]. Recent evidence confirms that activation of NF–κB promotes human breast cancer progression. For that reason, NF–κB has emerged as a potential therapeutic target in breast cancer treatment [4,5]. The NF–κB family comprises five transcription factors: NF–κB1/p50, NF–κB2/p52, RelA/p65, RelB, and c–Rel [6]. These factors can either hetero- or homodimerize to produce NF–κB complexes. The p65 subunit of NF–κB is a critical component in activating and regulating downstream target genes. Most of them are found in the cytoplasm of dormant cells; when they are activated, they move to the nucleus for transcription, which in turn causes hundreds of genes to be activated or repressed directly, indirectly, or both [7].

NF–κB is activated by viral and bacterial antigens, UV radiation, and cytokines such as IL–2 and TNF–α. The nuclear factor supports cell proliferation, apoptosis, and immunological responses to infection and inflammation. However, system disruption is associated with disorders including cancer, immunosuppression, and chronic inflammation [8]. NF–κB activation promotes cell survival by inhibiting apoptosis (programmed cell death). It controls the expression of anti-apoptotic proteins that aid cancer cells to avoid cell death and promote their survival, including Bcl–2, Bcl–xL, and inhibitors of apoptosis (IAPs) [9]. Additionally, NF–κB signaling promotes cell proliferation by increasing the expression of genes essential for cell cycle progression, including cyclins and cyclin-dependent kinases (CDKs) [10,11]. 

**Figure 1:**
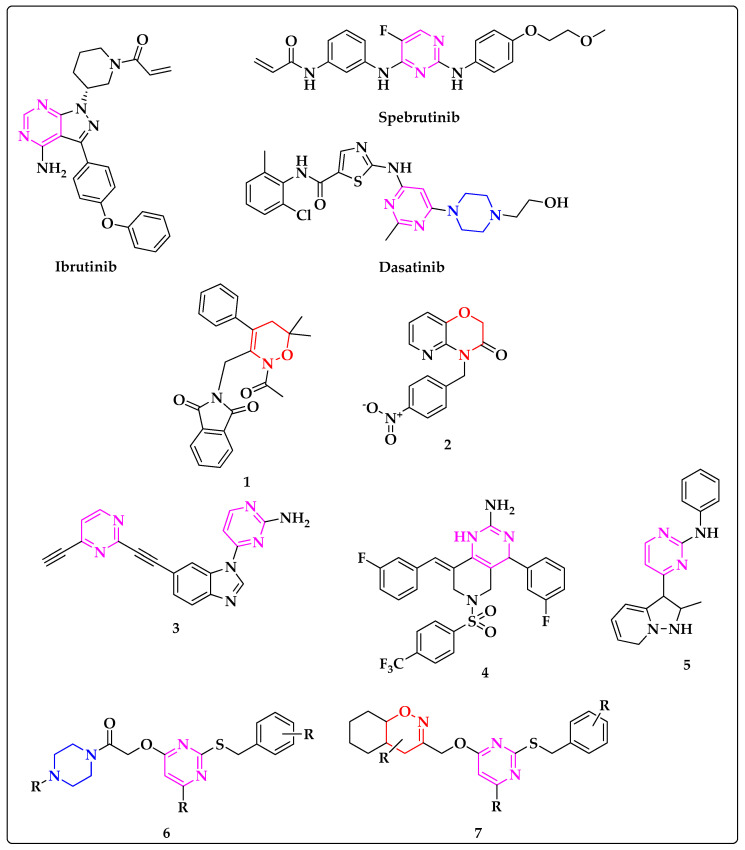
Marketed drugs and potent inhibitors of NF–κB. (**1**, **2**) bearing oxazines (red), (**3, 4, 5**) pyrimidine (pink) motifs are reported inhibitors. **6** and **7** are newly synthesized piperazine (blue)– and oxazine (red)–linked pyrimidine (pink) derivatives as NF–κB inhibitors.

It has been demonstrated that several drugs, including aspirin, sodium salicylate, and dexamethasone, decrease NF–κB activation by preventing the breakdown of IκB [12,13,14]. The current anti-TNF–α antibodies approved by the FDA include infliximab, adalimumab, and golimumab [15]. Ongoing research is being conducted to produce innovative small molecules that target the NF–κB pathway. New compounds and therapeutic strategies are continually explored and may emerge as potential candidates for further investigation in breast cancer treatment [16]. Piperazines derivatives, among other heterocycles, were discovered as promising anti-cancer agents [17,18,19,20], and many of the FDA-approved drugs include piperazines [21]. Novel piperazine compounds could suppress NF–κB translocation to the nucleus [22] and inhibit NF–κB by decreasing TNF–α levels [23]. Pyrimidines also play a vital role in anti-cancer drug discovery [24]. Ibudilast, spebrutinib, and dasatinib are a few pyrimidine-based drugs (Figure 1) that block the NF–κB pathway [25]. Furthermore, many reports have shown that oxazine derivatives might emerge as promising anti-cancer agents [26,27], and that they are potential candidates for NF–κB inhibitors [28,29,30]. The oxazine derivative compound **1** decreased the DNA binding ability of NF–κB and NF–κB-dependent luciferase expression and IκBα phosphorylation in hepatocellular carcinoma (HCC) and HCT116 cells. Furthermore, treatment of inflammatory bowel disease (IBD)-induced mice with compound **1** decreased myeloperoxidase activity in colonic extracts and modulated the colon length and serum levels of cytokines such as TNF–α, IFN–γ, IL–6, IL–1β, and IL–10 [31,32]. Similarly, compound **2** inhibited proliferation in HepG2, HCCLM3, and Huh–7 cells in a dose- and time-dependent manner, as well as decreased p65 subunit DNA binding capacity, p65 phosphorylation, and the consequent production of NF–κB-dependent luciferase gene expression in several HCC cell lines [33]. From the abovementioned discoveries, Lys28 was observed to be the active site of the p65 subunit in NF–κB. Benzimidazole-clubbed pyrimidines (**3**) were demonstrated as covalent inhibitors of cysteine in NF–κB inducing kinase [34]. Pyrido–pyrimidine (**5**) inhibited NF–κB activation by suppressing IκBα and LPS-induced phosphorylation levels of p65 and Akt, and by indirectly suppressing the MAPK signaling pathway [35], and pyralopyridine (**5**)-substituted pyrimidines were discovered as NF–κB transcription inhibitors [36].

Herein, we have synthesized novel oxazine– and piperazine–linked pyrimidine small molecules using thiouracils active in breast cancer cells (MCF–7) that target NF–κB. Alamar Blue assay showed that newly synthesized compounds **3a** and **5b** produced an IC_50_ of 9.17 and 6.29 µM in MCF–7 cells. In silico docking studies showed that compounds **3a** and **5b** exhibited −9.32 and −7.32 kcal mol^−1^ binding energy. Lys28 of the p65 subunit of NF–κB and the pyrimidine ring of **3a** and 4–methoxy benzyl thiol group of **5b** showed strong pi–alkyl interactions.

## 2. Materials and Methods

All chemicals and solvents were purchased from Sigma-Aldrich (Bangalore, India). The completion of the reaction was monitored by pre-coated silica gel TLC plates. An Agilent mass spectrophotometer was used to record the mass of the synthesized compounds. ^1^H and ^13^C NMR (Santa Clara, CA, USA) were recorded on Agilent and Jeol NMR spectrophotometers (400 MHz). TMS was used as an internal standard, and DMSO was used as a solvent. Chemical shifts were expressed as ppm.

### 2.1. General Procedure for the Synthesis of Oxazine and Piperazine Clubbed Pyrimidine Derivatives

#### 2.1.1. Synthesis of Compound **2**

Substituted thiouracils (**1**) (1.0 mmol) and various benzyl chlorides (1.2 mmol) were refluxed with KOH (1.2 mmol) in EtOH: H_2_O (1:1) as a solvent for 1 h. After the completion of the reaction, the solid mass was filtered off and washed with aqueous NaHCO_3_ solution and water, yielding compound **2**.

#### 2.1.2. Synthesis of Compound **3**

Compound **2** (1.0 mmol), substituted oxazines (1.0 mmol), and K_2_CO_3_ (2 mmol) were refluxed in acetone for 2–3 h. After the completion of the reaction, the crude reaction mass was extracted to ethyl acetate (25 mL × 3). The combined organic layer was distilled under reduced pressure and purified by column chromatography using ethyl acetate and hexane.

#### 2.1.3. Synthesis of Compounds **4**/**5**

Compound **2** (1.0 mmol) was treated with *tert*–butyl bromoacetate (1.2 mmol) and K_2_CO_3_ (1.5 mmol) in refluxing DMF. After the completion of the reaction, reaction mass was extracted with ethyl acetate and the crude product was purified by column chromatography, yielding compound **4**. The solution of **4** in trifluoroacetic acid was stirred at room temperature for 1–2 h. After completion of the reaction, it was quenched with sodium bicarbonate and extracted with ethyl acetate. The solid formed was filtered off and dried, yielding compound **5**.

#### 2.1.4. Synthesis of Compound **5a**

Compound **4** (1.0 mmol) was treated with 2–((2–((4–methoxybenzyl)thio) –pyrimidine–4–yl)oxy)–1–(4–methyl piperazin–1–yl)ethanone with EDC.HCl and DMAP as catalysts in basic conditions in DCM as solvent under nitrogen atmosphere for 2 h. After completion of the reaction, the crude mass was extracted with ethyl acetate, and the combined organic layer was distilled off and purified through column chromatography.

#### 2.1.5. Synthesis of Compound **5b**/**f**/**k**/**o**

Compound **4** (1.0 mmol) was treated with acetyl piperazines (1.2 mmol) with EDC.HCl and DMAP as catalysts in basic conditions in DCM as solvent under nitrogen atmosphere for 2 h. After completion of the reaction, the crude mass was extracted with ethyl acetate, and the combined organic layer was distilled off and purified through column chromatography.

#### 2.1.6. Synthesis of Compounds **5d**/**h**/**m**/**q**

Compound **4** (1.0 mmol) was treated with *N*–boc piperazines (1.2 mmol) with EDC.HCl and DMAP were used as catalysts in basic conditions in DCM as solvent under nitrogen atmosphere for 2 h. After completion of the reaction, the crude mass was extracted with ethyl acetate, and the combined organic layer was distilled off and purified through column chromatography, yields **5d**, **5h**, **5m,** or **5q**.

#### 2.1.7. Synthesis of Compounds **5c/g**/**l**/**p**

Compounds **5d**, **5h**, **5m,** or **5q** (1.0 mmol) were treated with trifluoroacetic acid. After completion of the reaction, the crude mass was neutralized with K_2_CO_3_ and extracted with ethyl acetate. The combined organic layer was distilled off and purified through column chromatography, yielding compounds **5c**, **5g**, **5l,** or **5p**, respectively.

#### 2.1.8. Synthesis of Compounds **5e**/**i**/**j**/**n**

Compounds **5c**, **5g**, **5l**, or **5p** (1 mmol) were treated with 5–bromopyridine–2–carboxylic acid (1.2 mmol) with EDC. HCl and DMAP were used as catalysts in basic conditions in DCM as solvent under nitrogen atmosphere for 2 h. After completion of the reaction, the crude mass was extracted with ethyl acetate, and the combined organic layer was distilled off and purified through column chromatography, yielding compounds **5e**, **5i**, **5j**, or **5n**, respectively.

#### 2.1.9. 6,6–Dimethyl–3–(((2–((3–methylbenzyl)thio)pyrimidin–4–yl)oxy)methyl)–4–phenyl –5,6–dihydro–4H–1,2–oxazine (**3a**)

Yellow solid; MP: 120–122 °C; ^1^H NMR (400 MHz, DMSO) δ 8.34 (s, 1H), 7.34–7.12 (m, 8H), 7.07 (s, 1H), 6.59 (s, 1H), 4.69 (s, 2H), 4.26 (s, 2H), 3.65 (s, 1H), 2.27 (s, 3H), 2.11 (s, 1H), 1.79 (t, *J* = 12.4, 24.8 Hz, 1H), 1.29 (s, 3H), 1.21 (s, 3H); ^13^C NMR (100 MHz, DMSO) δ 170.48, 167.99, 158.56, 153.96, 139.95, 138.00, 137.96, 129.86, 129.25, 128.79, 128.75, 128.19, 127.62, 126.31, 104.34, 79.64, 75.07, 65.73, 37.45, 34.58, 28.45, 22.94, 21.40; Calculated for C_25_H_27_N_3_O_2_S: actual = 433.5658, found = 434.1108 [M + 1]^+^.

#### 2.1.10. 4–(4–Methoxyphenyl)–3–(((2–((3–methylbenzyl)thio)pyrimidin–4–yl)oxy)methyl)-4a,5,–6,7,8,8a–hexahydro–4H–benzo[e][1,2]oxazine (**3b**)

Yellow solid; MP: 128–130 °C; ^1^H NMR (400 MHz, DMSO) δ 8.33 (s, 1H), 7.25–6.98 (m, 6H), 6.88 (s, 2H), 6.56 (s, 1H), 4.87 (s, 2H), 4.26 (s, 2H), 3.91 (s, 1H), 3.69 (s, 3H), 3.28 (s, 1H), 2.25 (s, 3H), 1.88 (s, 1H), 1.61 (s, 2H), 1.38–1.22 (m, 6H); ^13^C NMR (100 MHz, DMSO) δ 170.60, 168.12, 158.66, 158.63, 151.58, 138.00, 137.84, 133.15, 129.85, 129.62, 128.74, 128.17, 126.30, 114.55, 104.30, 69.03, 66.10, 55.43, 42.73, 38.67, 34.66, 28.99, 27.33, 24.68, 21.34, 20.24; Calculated for C_28_H_31_N_3_O_3_S: actual = 489.2086, found = 490.2234 [M + 1]^+^.

#### 2.1.11. 4–(4–Chlorophenyl)–6,6–dimethyl–3–(((2–((3–methylbenzyl)thio)pyrimidin–4–yl)oxy)–methyl)–5,6–dihydro–4H–1,2–oxazine (**3c**)

Yellow solid; MP: 130–132 °C; ^1^H NMR (400 MHz, DMSO) δ 8.34 (s, 1H), 7.32–7.17 (m, 7H), 7.07 (s, 1H), 6.57 (s, 1H), 4.72 (s, 2H), 4.27 (s, 2H), 3.69 (s, 1H), 2.28 (s, 3H), 2.11 (s, 1H), 1.77 (t, *J* = 24, 12 Hz, 1H), 1.29–1.20 (m, 6H); ^13^C NMR (100 MHz, DMSO) δ 170.45, 167.92, 158.59, 153.53, 138.90, 138.02, 137.95, 132.26, 130.72, 129.87, 129.16, 128.80, 128.21, 126.31, 104.34, 75.14, 65.63, 36.74, 34.59, 28.41, 22.97, 21.40; Calculated for C_25_H_26_ClN_3_O_2_S: actual = 467.1434, found = 468.1520 [M + 1]^+^.

#### 2.1.12. 3–(((2–((4–Chlorobezyl)thio)pyrimidin–4–yl)oxy)methyl)–6,6–dimethyl–4–phenyl–5,6–dihydro–4H–1,2–oxazine (**3d**)

Yellow solid; MP: 124–126 °C; ^1^H NMR (400 MHz, DMSO) δ 8.33 (s, 1H), 7.39–7.36 (m, 4H), 7.29 (s, 2H), 7.23 (s, 3H), 6.59 (s, 1H), 4.68 (s, 2H), 4.29 (s, 2H), 3.64 (s, 1H), 2.11 (s, 1H), 1.78 (t, *J* = 12.4, 24.4 Hz, 1H), 1.29 (s, 3H), 1.20 (s, 3H); ^13^C NMR (100 MHz, DMSO) δ 170.12, 168.05, 158.61, 153.92, 139.95, 137.61, 132.08, 131.04, 129.25, 128.80, 128.75, 127.62, 104.48, 75.09, 65.76, 37.42, 33.69, 28.45, 22.95; Calculated for C_24_H_24_ClN_3_O_2_S: actual = 453.9843, found = 454.1443 [M + 1]^+^.

#### 2.1.13. 3–(((2–((4–Chlorobezyl)thio)pyrimidin–4–yl)oxy)methyl)–4–(4–methoxyphenyl)–4a,5,6,–7,8,8a–hexahydro–4H–benzo[e][1,2]oxazine (**3e**)

Yellow solid; MP: 110–112 °C; ^1^H NMR (400 MHz, DMSO) δ 8.34 (s, 1H), 7.37 (d, *J* = 28.1 Hz, 4H), 7.11 (s, 2H), 6.89 (s, 2H), 6.59 (s, 1H), 4.85 (s, 2H), 4.31 (s, 2H), 3.91 (s, 1H), 3.72 (s, 3H), 2.52 (s, 1H), 1.91 (s, 1H), 1.64 (s, 2H), 1.40–1.22 (m, 6H); ^13^C NMR (100 MHz, DMSO) δ 170.20, 168.19, 158.72, 158.65, 151.59, 137.58, 133.19, 132.07, 131.03, 129.65, 128.77, 114.57, 104.47, 69.03, 66.16, 55.49, 42.67, 38.67, 33.73, 28.97, 27.31, 24.65, 20.25; Calculated for C_27_H_28_ClN_3_O_3_S: actual = 509.15, found = 510.1613 [M + 1]^+^.

#### 2.1.14. 3–(((2–((4–Chlorobezyl)thio)pyrimidin–4–yl)oxy)methyl)–4–(4–chlorophenyl)–6,6–di–methyl–5,6–dihydro–4H–1,2–oxazine (**3f**)

Yellow solid; MP: 106–108 °C; ^1^H NMR (400 MHz, DMSO) δ 8.34 (s, 1H), 7.40–7.26 (m, 8H), 6.58 (s, 1H), 4.71 (s, 2H), 4.31 (s, 2H), 3.68 (s, 1H), 2.10 (s, 1H), 1.76 (s, 1H), 1.24–1.20 (m, 6H); ^13^C NMR (100 MHz, DMSO) δ 170.12, 167.98, 158.62, 153.48, 138.90, 137.61, 132.26, 132.09, 131.04, 130.71, 129.16, 128.81, 104.47, 75.14, 65.67, 36.73, 33.70, 28.41, 22.98; Calculated for C_24_H_23_Cl_2_N_3_O_2_S: actual = 488.4293, found = 490.0965 [M + 1]^+^.

#### 2.1.15. 3–(((2–((4–Fluorobenzyl)thio)pyrimidin–4–yl)oxy)methyl)–6,6–dimethyl–4–phenyl–5,6–dihydro–4H–1,2–oxazine (**3g**)

Yellow solid; MP: 136–138 °C; ^1^H NMR (400 MHz, DMSO) δ 8.34 (s, 1H), 7.41 (s, 2H), 7.26 (m, 5H), 7.12 (s, 2H), 6.60 (d, *J* = 2.0 Hz, 1H), 4.68 (s, 2H), 4.29 (s, 2H), 3.65 (s, 1H), 2.17–2.04 (m, 1H), 1.78 (t, *J* = 12.4, 25.8 Hz, 1H), 1.29 (s, 3H), 1.20 (s, 3H); ^13^C NMR (100 MHz, DMSO) δ 170.25, 168.03, 162.91, 160.49, 158.59, 153.93, 139.96, 134.59, 134.56, 131.18, 131.10, 129.25, 128.75, 127.62, 115.73, 115.52, 104.44, 75.08, 65.75, 37.43, 33.67, 28.45, 22.94; Calculated for C_24_H_24_FN_3_O_2_S: actual = 437.5297, found = 438.1714 [M + 1]^+^.

#### 2.1.16. 3–(((2–((4–Fluorobenzyl)thio)pyrimidin–4–yl)oxy)methyl)–4–(4–methoxyphenyl)–4a,5,6,–7,8, 8a–hexahydro–4H–benzo[e][1,2]oxazine (**3h**)

C_27_H_28_FN_3_O_3_S; Yellow solid; MP: 142–144 °C; ^1^H NMR (400 MHz, DMSO) δ 8.35 (s, 1H), 7.42 (s, 2H), 7.11 (s, 4H), 6.89 (s, 2H), 6.59 (s, 1H), 4.85 (s, 2H), 4.30 (s, 2H), 3.91 (s, 1H), 3.71 (s, 3H), 3.28 (s, 1H), 1.90 (d, *J* = 10.0 Hz, 1H), 1.64 (s, 2H), 1.40–1.24 (m, 6H); ^13^C NMR (100 MHz, DMSO) δ 170.33, 168.18, 162.90, 160.48, 158.73, 158.65, 151.62, 134.54, 134.51, 133.19, 131.19, 131.11, 129.66, 115.71, 115.50, 114.57, 104.45, 69.05, 66.12, 55.49, 42.65, 36.68, 33.71, 28.96, 27.31, 24.64, 20.26; Calculated for C_27_H_28_FN_3_O_3_S: actual = 493.5929, found = 494.1919 [M + 1]^+^.

#### 2.1.17. 4–(4–Chlorophenyl)–3–(((2–((4–fluorobenzyl)thio)pyrimidin–4–yl)oxy)methyl)–6,6–dimethyl–5,6–dihydro–4H–1,2–oxazine (**3i**)

C_24_H_23_ClFN_3_O_2_S; Yellow solid; MP: 120–122 °C; ^1^H NMR (400 MHz, DMSO) δ 8.34 (s, 1H), 7.42 (s, 2H), 7.29 (m, 4H), 7.13 (s, 2H), 6.58 (s, 1H), 4.71 (s, 2H), 4.31 (s, 2H), 3.69 (s, 1H), 2.10 (s, 1H), 1.76 (t, *J* = 11.6, 23.6 Hz, 1H), 1.29–1.20 (m, 6H); ^13^C NMR (100 MHz, DMSO) δ 170.24, 167.95, 162.91, 160.49, 158.61, 153.50, 138.90, 134.58, 134.55, 132.26, 131.19, 131.11, 130.72, 129.16, 115.74, 115.53, 104.43, 75.14, 65.64, 36.72, 33.67, 28.40, 22.96; Calculated for C_24_H_23_ClFN_3_O_2_S: actual = 471.12, found = 472.1271 [M + 1]^+^.

#### 2.1.18. 3–(((2–((4–Fluorobenzyl)thio)–6–methylpyrimidin–4–yl)oxy)methyl)–6,6–dimethyl–4–phenyl–5,6–dihydro–4H–1,2–oxazine (**3j**)

Yellow solid; MP: 130–132 °C; ^1^H NMR (400 MHz, DMSO) δ 7.41 (s, 2H), 7.30–7.22 (m, 5H), 7.12 (s, 2H), 6.45 (s, 1H), 4.66 (s, 2H), 4.27 (s, 2H), 3.62 (s, 1H), 2.31 (s, 3H), 2.11 (s, 1H), 1.77 (t, *J* = 12, 23.2 Hz, 1H), 1.29 (s, 3H), 1.19 (s, 3H); ^13^C NMR (100 MHz, DMSO) δ 169.49, 168.62, 168.54, 162.86, 160.44, 154.11, 140.01, 134.85, 134.82, 131.22, 131.14, 129.26, 128.73, 127.60, 115.68, 115.46, 102.72, 75.05, 65.68, 37.40, 33.58, 28.44, 23.65, 22.89; Calculated for C_25_H_26_FN_3_O_2_S: actual = 451.5562, found = 452.1881 [M + 1]^+^.

#### 2.1.19. 3–(((2–((4–Fluorobenzyl)thio)–6–methylpyrimidin–4–yl)oxy)methyl)–4–(4–methoxy–phenyl)–4a,5,6,7,8,8a–hexahydro–4H–benzo[e][1,2]oxazine (**3k**)

Yellow solid; MP: 124–126 °C; ^1^H NMR (400 MHz, DMSO) δ 7.42 (s, 2H), 7.10 (s, 4H), 6.88 (s, 2H), 6.44 (s, 1H), 4.83 (s, 2H), 4.27 (s, 2H), 3.90 (s, 1H), 3.71 (s, 3H), 3.25 (s, 1H), 2.30 (s, 3H), 1.90 (d, *J* = 10.0 Hz, 1H), 1.63 (d, *J* = 7.2 Hz, 2H), 1.40–1.24 (m, 6H); ^13^C NMR (100 MHz, DMSO) δ 169.53, 168.78, 168.64, 162.84, 160.43, 158.62, 151.81, 134.78, 134.74, 133.16, 131.19, 131.11, 129.64, 115.64, 115.43, 114.54, 102.73, 69.03, 65.99, 55.46, 42.62, 38.61, 33.62, 28.95, 27.29, 24.62, 23.58, 20.22; Calculated for C_28_H_30_FN_3_O_3_S: actual = 507.6195, found = 508.2078 [M + 1]^+^.

#### 2.1.20. 4–(4–Chlorophenyl)–3–(((2–((4–fluorobenzyl)thio)–6–methylpyrimidin–4–yl)oxy)–methyl)–6,6–dimethyl–5,6–dihydro–4H–1,2–oxazine (**3l**)

Yellow solid; MP: 100–102 °C; ^1^H NMR (400 MHz, DMSO) δ 7.42 (s, 2H), 7.33 (s, 2H), 7.25 (s, 2H), 7.12 (s, 2H), 6.42 (s, 1H), 4.69 (d, *J* = 11.6 Hz, 2H), 4.28 (s, 2H), 3.66 (s, 1H), 2.31 (s, 3H), 2.10 (s, 1H), 1.75 (t, *J* = 12.4, 24.4 Hz, 1H), 1.29 (s, 3H), 1.19 (s, 3H); ^13^C NMR (100 MHz, DMSO) δ 169.48, 168.65, 168.47, 162.87, 160.45, 153.69, 138.95, 134.82, 132.24, 131.21, 131.13, 130.70, 129.16, 115.68, 115.47, 102.70, 75.11, 65.61, 36.73, 33.60, 28.40, 23.67, 22.92; Calculated for C_25_H_25_ClFN_3_O_2_S: actual = 485.13, found = 486.1400 [M + 1]^+^.

#### 2.1.21. 1,1′–(Piperazine–1,4–diyl)bis(2–((2–((4–methoxybenzyl)thio)pyrimidin–4–yl)oxy)ethan–one) (**5a**)

White solid; MP: 150–152 °C; ^1^H NMR (400 MHz, CDCl_3_): δ 8.30 (d, *J* = 5.2 Hz, 1H), 7.32 (d, *J* = 7.6 Hz, 2H), 6.85 (d, *J* = 8.0 Hz, 2H), 6.55 (d, *J* = 5.2 Hz, 1H), 4.90 (s, 2H), 4.30 (s, 2H), 3.79 (s, 3H), 3.75 (s, 7H); ^13^C NMR (400 MHz, CDCl_3_) δ 171.5, 168.7, 167.7, 159.0, 158.0, 130.1, 129.2, 114.1, 103.9, 62.6, 55.4, 52.3, 34.9. 

#### 2.1.22. 1–(4–Acetylpiperazin–1–yl)–2–((2–((4–methoxybenzyl)thio)pyrimidin–4–yl)oxy)ethan–one (**5b**)

Yellow thick mass; ^1^H NMR (400 MHz, CDCl_3_) δ 8.29 (d, *J* = 5.4 Hz, 1H), 7.30 (d, *J* = 8.0 Hz, 2H), 6.83 (d, *J* = 8.0 Hz, 2H), 6.56 (d, *J* = 5.5 Hz, 1H), 4.99 (s, 2H), 4.32 (s, 2H), 3.78 (s, 3H), 3.51 (d, *J* = 68.9 Hz, 8H), 2.09 (s, 3H); ^13^C NMR (100 MHz, CDCl_3_): δ 171.3, 169.4, 167.7, 165.9, 159.0, 157.9, 130.1, 129.3, 114.1, 104.1, 63.1, 55.4, 46.0, 44.7, 42.0, 41.3, 34.8, 21.4.

#### 2.1.23. Tert–butyl 4–(2–((2–((4–Methoxybenzyl)thio)pyrimidin–4–yl)oxy)acetyl)piperazine–1–carboxylate (**5c**)

White solid; MP: 160–162 °C; ^1^H NMR (400 MHz, CDCl_3_) δ 8.28 (d, *J* = 5.7 Hz, 1H), 7.30 (d, *J* = 8.4 Hz, 2H), 6.82 (d, *J* = 8.5 Hz, 2H), 6.56 (d, *J* = 5.7 Hz, 1H), 4.98 (s, 2H), 4.31 (s, 2H), 3.77 (s, 3H), 3.57 (s, 2H), 3.42 (d, *J* = 18.1 Hz, 6H), 1.45 (s, 9H); 13C NMR (100 MHz, CDCl_3_) δ 170.7, 167.1, 165.0, 158.3, 157.3, 153.9, 129.5, 128.6, 113.5, 103.5, 79.9, 62.6, 54.8, 44.2, 41.3, 34.3, 27.8; MS: 474.58, *m*/*z* = 475.12 [M + 1]^+^.

#### 2.1.24. 1–(4–(5–Bromopicolinoyl)piperazin–1–yl)–2–((2–((4–methoxybenzyl)thio)pyramidin–4–yl)–oxy)ethanone (**5d**)

Yellow thick mass; ^1^H NMR (400 MHz, CDCl_3_) δ 8.629 (S, 1H), 8.289 (d, *J* = 4.0 Hz, 1H), 7.94 (d, *J* = 6.8 Hz, 1H), 7.73–7.58 (m, 1H), 7.30 (d, *J* = 7.2 Hz, 2H), 6.82 (d, *J* = 6.4 Hz, 2H), 6.60 (d, *J* = 4.4 Hz, 1H), 5.17–4.93 (s, 2H), 4.31 (s, 2H), 3.82–3.51 (m, 11H); ^13^C NMR (100 MHz, CDCl_3_) δ 170.7, 167.1, 165.0, 158.3, 157.6, 157.3, 150.9, 148.8, 139.4, 130.9, 129.5, 128.3, 122.0, 113.4, 103.4, 62.5, 54.8, 46.4, 42.0, 34.2; MS: 558.07, *m*/*z* = 559.99 [M + 1]^+^.

#### 2.1.25. 1–(4–Acetylpiperazin–1–yl)–2–((2–((3–methylbenzyl)thio)pyrimidin–4–yl)oxy)–ethanone (**5e**)

Yellow thick mass; ^1^H NMR (500 MHz, CDCl_3_) δ 8.29 (d, *J* = 5.5 Hz, 1H), 7.20–7.14 (m, 3H), 7.04 (d, *J* = 5.5 Hz, 1H), 6.56 (d, *J* = 5.5 Hz, 1H), 4.98 (s, 2H), 4.32 (s, 2H), 3.64–3.58 (m, 4H), 3.47–3.41 (m, 4H), 2.31 (s, 3H), 2.09 (s, 3H); ^13^C NMR (100 MHz, CDCl_3_) 171.2, 169.4, 167.7, 165.9, 157.9, 138.4, 137.2, 129.6, 128.5, 128.2, 125.9, 104.1, 63.0, 46.0, 44.7, 42.0, 41.2, 35.3, 29.8, 21.4.

#### 2.1.26. 2–((2–((3–Methylbenzyl)thio)pyrimidin–4–yl)oxy)–1–(piperazin–1–yl)ethanone (**5f**)

Yellow thick mass; ^1^H NMR (500 MHz, CDCl_3_) δ 8.28 (d, *J* = 5.5 Hz, 1H), 7.25–7.17 (m, 3H), 7.08–7.01 (m, 1H), 6.56 (d, *J* = 5.5 Hz, 1H), 4.97 (s, 2H), 4.32 (s, 2H), 3.56 (s, 2H), 3.44 (s, 2H), 3.39 (s, 4H), 2.31 (s, 3H), 1.45 (s, 9H); ^13^C NMR (100 MHz, CDCl_3_) δ 171.3, 167.8, 165.6, 157.9, 154.5, 138.3, 137.2, 129.6, 128.5, 128.1, 126.0, 104.1, 80.5, 63.2, 44.8, 43.8, 41.9, 35.3, 28.4, 21.4.

#### 2.1.27. Tert–butyl–4–(2–((2–((3–methylbenzyl)thio)pyrimidin–4–yl)oxy)acetyl)piperazine–1–car–boxylate (**5g**)

White solid; MP: 166–168 °C; ^1^H NMR (500 MHz, CDCl_3_) δ 8.30 (d, *J* = 6.0 Hz, 1H), 7.20–7.17 (m, 3H), 7.05–7.03 (m, 1H), 6.56 (d, *J* = 5.5 Hz, 1H), 4.97 (s, 2H), 4.32 (s, 2H), 3.57 (s, 2H), 3.39 (s, 2H), 2.87–2.82 (m, 4H), 2.31 (s, 3H); ^13^C NMR (100 MHz, CDCl_3_) δ 171.2, 167.8, 165.4, 157.7, 138.3, 137.2, 129.7, 128.4, 128.0, 126.0, 104.1, 63.1, 45.9, 42.9, 35.3, 21.5.

#### 2.1.28. 1–(4–(5–Bromopicolinoyl)piperazin–1–yl)–2–((2–((3–methylbenzyl)thio)pyrimidin–4–yl)–oxy)–ethanone (**5h**)

Yellow thick mass; ^1^H NMR (500 MHz, CDCl_3_) δ 8.62–8.59 (m, 1H), 8.28 (d, *J* = 5.5 Hz, 1H), 7.93 (dd, *J* = 8.5, 2.0 Hz, 1H), 7.61 (dd, *J* = 16.0, 8.5 Hz, 1H), 7.22–7.13 (m, 3H), 7.07–7.01 (m, 1H), 6.56 (d, *J* = 5.5 Hz, 1H), 5.01–4.97 (m, 2H), 4.32 (s, 2H), 3.81–3.72 (m, 4H), 3.64 (s, 2H), 3.54–3.49 (m, 2H), 2.30 (s, 3H); ^13^C NMR (100 MHz, CDCl_3_) δ 171.2, 167.7, 166.7, 165.8, 157.9, 151.4, 149.3, 149.2, 140.0, 138.3, 137.2, 129.6, 128.5, 128.2, 126.2, 126.0, 122.5, 104.1, 63.1, 47.1, 44.7, 42.6, 41.8, 35.3, 21.4.

#### 2.1.29. 1–(4–(5–Bromopicolinoyl)piperazin–1–yl)–2–((2–((4–fluorobenzyl)thio)pyrimidin–4–yl)–oxy)–ethanone (**5i**)

Yellow thick mass; ^1^H NMR (500 MHz, CDCl_3_) δ 8.62–8.60 (m, 1H), 8.28 (d, *J* = 6.0 Hz, 1H), 7.93 (dd, *J* = 8.4, 2.3 Hz, 1H), 7.65–7.60 (m, 1H), 7.35–7.33 (m, 2H), 6.96 (t, *J* = 9.0 Hz, 2H), 6.56 (d, *J* = 5.5 Hz, 1H), 5.01–4.96 (m, 2H), 4.32 (s, 2H), 3.84–3.71 (m, 4H), 3.64 (s, 2H), 3.55–3.50 (m, 2H); ^13^C NMR (100 MHz, CDCl_3_): δ 170.9, 167.7, 166.7, 166.4, 165.8, 163.0, 161.1, 158.0, 157.9, 157.8, 151.4, 149.4, 140.1, 133.3, 130.5, 126.3, 126.0, 122.6, 115.6, 115.5, 115.3, 104.3, 104.2, 63.1, 47.1, 44.72, 42.7, 41.8, 34.5, 29.8.

#### 2.1.30. 1–(4–Acetylpiperazin–1–yl)–2–((2–((4–fluorobenzyl)thio)pyrimidin–4–yl)oxy)–ethanone (**5j**)

Yellow thick mass; ^1^H NMR (500 MHz, CDCl_3_) δ 8.29 (d, *J* = 5.5 Hz, 1H), 7.35–7.33 (m, 2H), 6.97 (t, *J* = 8.6 Hz, 2H), 6.57 (d, *J* = 5.5 Hz, 1H), 4.98 (s, 3H), 4.32 (s, 3H), 3.60 (s, 5H), 3.42 (s, 5H), 2.10 (s, 4H); ^13^C NMR (100 MHz, CDCl_3_) δ 170.8, 169.4, 167.7, 165.8, 163.1, 161.1, 157.9, 133.3, 130.6, 130.5, 115.5, 115.4, 104.3, 77.4, 77.1, 76.9, 63.0, 45.9, 44.8, 42.0, 41.3, 34.4, 21.4.

#### 2.1.31. 2–((2–((4–Fluorobenzyl)thio)pyrimidin–4–yl)oxy)–1–(piperazin–1–yl)ethanone (**5k**)

Yellow thick mass; ^1^H NMR (500 MHz, CDCl_3_) δ ^1^H NMR (500 MHz) δ 8.26 (d, *J* = 5.5 Hz, 1H), 7.33 (dd, *J* = 8.4, 5.5 Hz, 2H), 6.95 (t, *J* = 8.7 Hz, 2H), 6.55 (d, *J* = 5.5 Hz, 1H), 4.95 (s, 2H), 4.31 (s, 2H), 3.54 (s, 2H), 3.37 (s, 2H), 2.84–2.80 (m, 4H), 2.05 (s, 1H); ^13^C NMR (100 MHz, CDCl_3_) δ 170.8, 167.9, 165.3, 163.0, 161.1, 157.8, 133.3, 133.3, 130.6, 130.5, 115.5, 115.4, 104.3, 63.2, 46.2, 46.0, 45.8, 43.1, 34.5.

#### 2.1.32. Tert–butyl–4–(2–((2–((4–fluorobenzyl)thio)pyrimidin–4–yl)oxy)acetyl)piperazine–1–car–boxylate (**5l**)

White solid; MP: 156–158 °C; ^1^H NMR (500 MHz, CDCl_3_) δ 8.28 (d, *J* = 5.5 Hz, 1H), 7.32 (d, *J* = 8.0 Hz, 2H), 7.25 (d, *J* = 4.5 Hz, 2H), 6.56 (d, *J* = 5.5 Hz, 1H), 4.96 (s, 2H), 4.31 (s, 2H), 3.56 (s, 2H), 3.45 (s, 2H), 3.39 (s, 4H), 1.45 (s, 9H); ^13^C NMR (100 MHz, CDCl_3_) δ 170.7, 167.8, 165.5, 157.9, 154.5, 136.2, 133.1, 130.3, 128.7, 104.3, 80.6, 63.2, 44.8, 41.9, 34.5, 28.4.

#### 2.1.33. 1–(4–(5–Bromopicolinoyl)piperazin–1–yl)–2–((2–((4–chlorobenzyl)thio)pyrimidin–4–yl)–oxy)–ethanone (**5m**)

Yellow thick mass; ^1^H NMR (500 MHz, CDCl_3_) δ 8.61 (d, *J* = 5.5 Hz, 1H), 8.28 (d, *J* = 5.5 Hz, 1H), 7.94 (d, *J* = 8.0 Hz, 1H), 7.32–7.60 (m, 1H), 7.31 (d, *J* = 8.0 Hz, 2H), 7.24 (d, *J* = 8.0 Hz, 2H), 6.56 (d, *J* = 4.8 Hz, 1H), 5.00–4.95 (m, 2H), 4.31 (s, 2H), 3.82–3.73 (m, 4H), 3.64 (s, 2H), 3.55–3.50 (m, 2H); ^13^C NMR (100 MHz, CDCl_3_) δ 170.7, 167.7, 166.7, 165.7, 157.9, 151.5, 149.4, 140.0, 136.2, 133.0, 130.3, 128.7, 126.0, 122.6, 104.3, 63.1, 47.1, 45.3, 44.7, 42.6, 34.5.

#### 2.1.34. 1–(4–Acetylpiperazin–1–yl)–2–((2–((4–chlorobenzyl)thio)pyrimidin–4–yl)oxy)–ethanone (**5n**)

Yellow thick mass; ^1^H NMR (500 MHz, CDCl_3_) δ 8.28 (d, *J* = 5.5 Hz, 1H), 7.32 (d, *J* = 8.0 Hz, 2H), 7.25 (d, *J* = 4.8 Hz, 2H), 6.57 (d, *J* = 5.5 Hz, 1H), 4.97 (s, 2H), 4.31 (s, 2H), 3.66–3.60 (m, 4H), 3.42 (s, 4H), 2.10 (s, 3H); ^13^C NMR (100 MHz, CDCl_3_) δ 170.7, 169.4, 167.8, 165.8, 158.0, 136.2, 133.1, 130.3, 128.7, 104.3, 63.0, 46.0, 44.8, 42.0, 41.3, 34.5, 21.4.

#### 2.1.35. 2–((2–((4–Chlorobenzyl)thio)pyrimidin–4–yl)oxy)–1–(piperazin–1–yl)ethanone (**5o**)

Yellow thick mass; ^1^H NMR (500 MHz, CDCl_3_) δ 8.26 (d, *J* = 5.5 Hz, 1H), 7.31 (d, *J* = 8.0 Hz, 2H), 7.24 (d, *J* = 8.0 Hz, 2H), 6.56 (d, *J* = 5.5 Hz, 1H), 4.95 (s, 2H), 4.31 (s, 2H), 3.56 (s, 2H), 3.38 (s, 2H), 2.86–2.81 (m, 4H); ^13^C NMR (100 MHz, CDCl_3_) 170.7, 167.9, 165.3, 157.8, 136.2, 133.1, 130.3, 128.7, 104.3, 63.2, 46.0, 43.0, 34.6.

#### 2.1.36. Tert–butyl–4–(2–((2–((4–chlorobenzyl)thio)pyrimidin–4–yl)oxy)acetyl)piperazine–1–car–boxylate (**5p**)

White solid; MP: 160–170 °C;^1^H NMR (500 MHz, CDCl_3_) δ 8.28 (d, *J* = 5.5 Hz, 1H), 7.32 (d, *J* = 8.0 Hz, 2H), 7.25 (d, *J* = 4.5 Hz, 2H), 6.56 (d, *J* = 5.5 Hz, 1H), 4.96 (s, 2H), 4.31 (s, 2H), 3.56 (s, 2H), 3.45 (s, 2H), 3.39 (s, 4H), 1.61 (s, 3H), 1.45 (s, 9H); ^13^C NMR (100 MHz, CDCl_3_) δ 170.7, 167.8, 165.6, 157.9, 154.5, 136.2, 133.1, 130.3, 128.7, 104.3, 80.6, 63.2, 44.9, 41.9, 34.5, 28.4. 

### 2.2. Cell Viability Assay

MCF-7, MDAMB-231, BT549, and SUM159PT cells were purchased from Procell Life Science & Technology Co., Ltd. (Wuhan, China). All carcinoma cell lines were cultured according to ATCC propagation instructions. By following the procedure in Basappa et al. [37], first, 2 × 10^3^ MCF-7 cells in 200 μL were grown in MEM enriched with 2% FBS and kept at 37 °C in a humidified 5% CO_2_ environment. The compounds (10 mM) were dissolved in DMSO and were stored as a stock solution. The DMSO and the stock solution of compounds were diluted to 0.01, 0.1, 10, 100, and 1000 μM solutions in cell culture medium, keeping a DMSO amount less than 1%. MCF-7 cells (2 × 10^3^) were incubated for 72 h with exposure to pyrimidines and Alamar Blue reagent was used to evaluate cell viability.

### 2.3. Annexin V Apoptosis and Cell Cycle Analysis Assay

MCF-7 cells were cultured at a density of approximately 1 × 10^5^ cells per well on a six cm tissue culture petri dish, and treated with compounds **3a** or **5b** for 72 h. Following the procedure, attached and floating cells were gathered and rinsed twice with ice-cold phosphate buffer solution. The degree of apoptosis was determined using the Annexin V-AbFluorTM 488/PI Apoptosis Detection Kit (Abbkine, KTA0002, Wuhan, China) following the manufacturer’s instructions. A quantity of 1 × 10^5^ cells were collected, washed once in ice-cold PBS, and permeabilized with 100 L of 0.5% Triton X-100 to evaluate cell cycle distribution. A quantity of 1 × 10^5^ cells werefixed with 75% ethanol at −20 °C overnight and stained with 50 µg/mL PI in 200 µL PBS supplemented with 20 μg/mL (*w*/*v*) RNase A (Abbkine, KTA2020, Wuhan, China) for 1 h at 4 °C. Cytofluorometric acquisitions were performed on a BECKMAN COULER CytoFlex at a low flow rate mode. 

### 2.4. Western Blot Analysis

MCF-7 cells were treated with compounds **3a** or **5b** and harvested, and the cell lysates were obtained. SDS-PAGE was used to separate the proteins of interest, and were transferred onto PVDF (Millipore, ISEQ00010, Burlington, MA, USA) membrane. The membrane was sequentially incubated with primary and secondary antibodies and the corresponding proteins were visualized using an ECL kit Clarity™ and Clarity Max™ Western ECL Blotting Substrates (BIO-RAD, Hercules, CA, USA).

### 2.5. Data Analysis and Statistics

The results are presented as mean ± standard deviation. A one-way analysis of variance (one-way ANOVA), with Bonferroni’s multiple comparison tests, was used to analyze the statistical change between treatment groups. A 0.05 confidence level was the significant change cutoff.

### 2.6. Molecular Docking

The docking studies were determined by using The Scripps Research Institute’s AutoDock4 tools (v.1.5.6) [38]. Initially, **3a** or **5b** structures were obtained from the molecular drawing software tools, and the compounds were converted to the PDBQT format. Later protein preparation was performed by BIOVIA Discovery studio. Before this, the crystal structure of NF–κB (PDB ID: 1IKN) was retrieved from the Protein Data Bank. The protein structure was prepared by removing water molecules and adding hydrogen atoms. Kollman charges were assigned to the protein. Later ligand preparations were performed for both compounds **3a** and **5b** and further used for docking purposes. Docking simulations were performed using AutoDock4. The Lamarckian Genetic Algorithm was employed for both ligands. The grid box was defined around the active site of the NF–kappa B p65 subunit, and the grid dimensions were 40 Å × 40 Å × 28 Å with a spacing of 0.375 Å. The docking parameters were set to default values, and 10 docking runs were performed for each compound. Later, the resulting docking poses were visualized using BIOVIA Discovery Studio [39], PyMOL [40], and UCSF Chimera1.16 [41]. 

## 3. Results 

### 3.1. Synthesis of Piperazine- and Oxazine-Linked Pyrimidine Derivatives

**Scheme 1:**
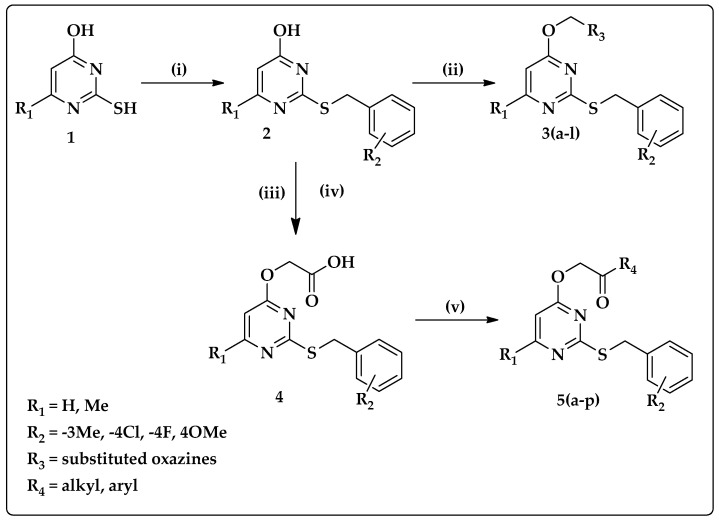
Synthesis of oxazine- and piperazine-clubbed pyrimidine. Reaction Conditions: (i) substituted benzyl chlorides (1.2 mmol), KOH (1.4 mmol), EtOH: H_2_O (1:1), reflux; (ii) substituted oxazines (1.0 mmol), K_2_CO_3_ (2.0 mmol), acetone, reflux; (iii) *tert*–butylbromoacetate (1.2 mmol), K_2_CO_3_ (2.0 mmol), DMF, reflux; (iv) TFA, rt, (v) substituted amines, EDC.HCl, DMAP, Et_3_N, DCM, rt.

**Figure 2:**
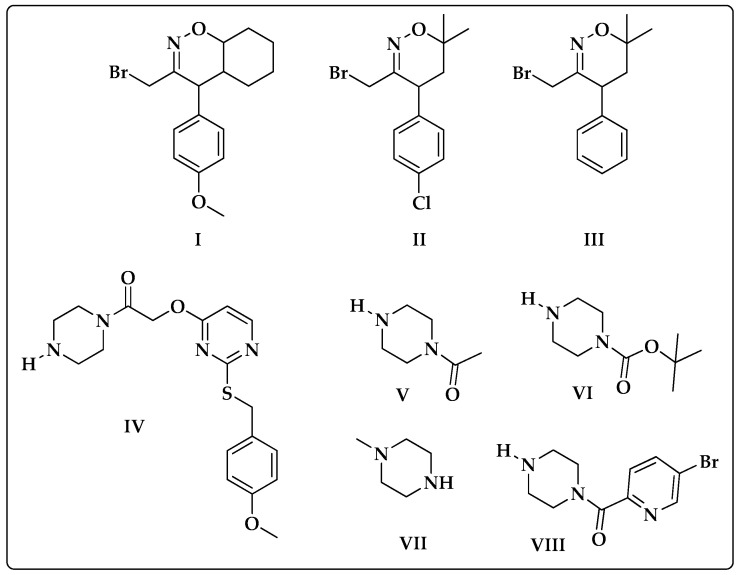
Structures of (**I**–**VIII**) substituent.

S–Benzylated of 2–thiouracil (**2**) was synthesized by refluxing substituted–2–thiouracil (**1**) and substituted benzyl chloride in EtOH:H_2_O at basic conditions. Compound (**2**) was treated with substituted oxazine–bromides (**I**, **II**, **III**) in acetone under basic conditions, yielding thiouracil–oxazine hybrids **3**(**a**–**l**). Also, refluxing compound (**2**) with *tert*–butylbromoacetate in DMF and further deprotection by TFA yielded compound (**4**). Compound **4** on acid–amine coupling with substituted piperazines (**IV**, **V**, **VI**, **VII**, and **VIII**) yielded derivatives **5**(**a**–**p**)(Scheme 1, Figure 2). All the synthesized compounds are characterised by spectroscopic technique (See supplementary file).

### 3.2. Efficacy of Pyrimidine Derivatives in Breast Cancer Cells

**Figure 3:**
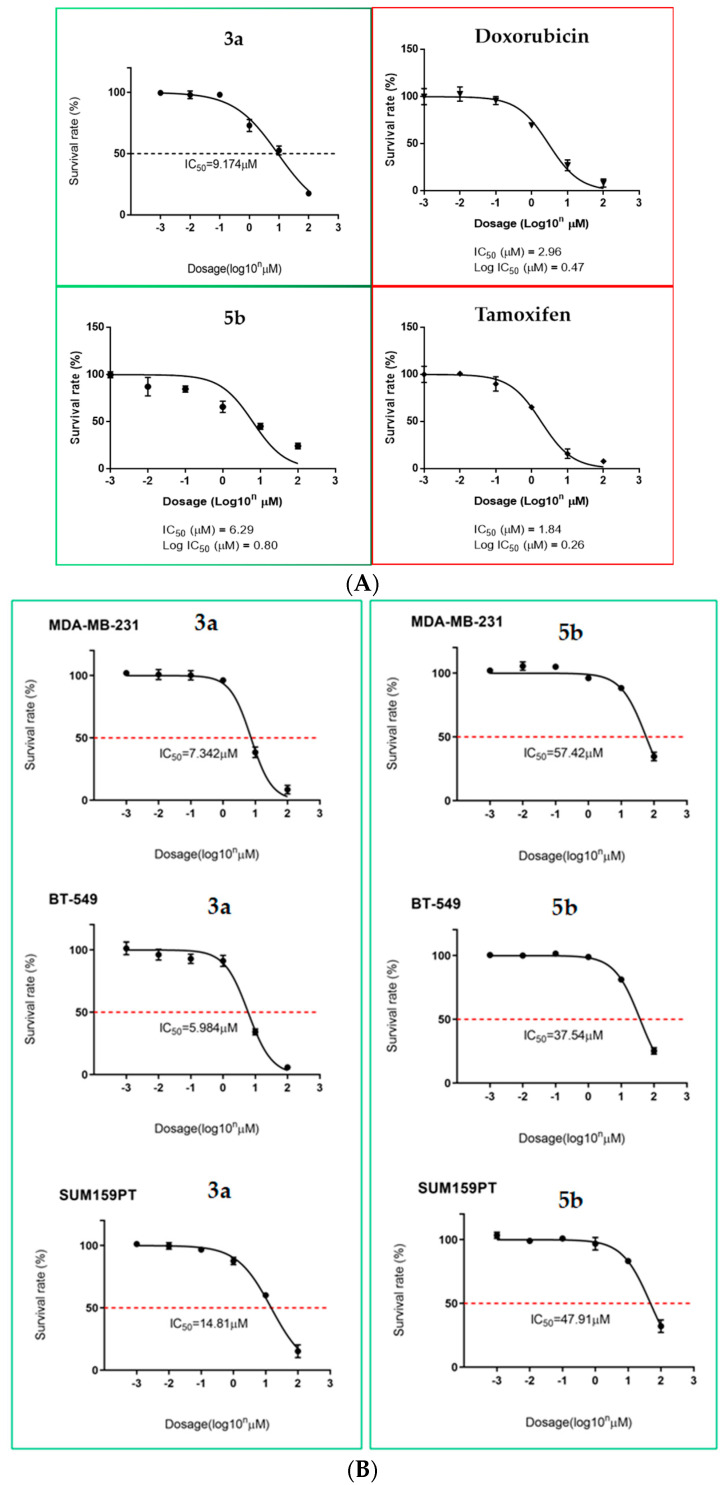
(**A**) IC_50_ of compounds **3a**, **5b**, doxorubicin and tamoxifen in MCF-7 breast cancer cells. (**B**) Lead compounds, **3a** and **5b,** produce loss of cell viability in MDAMB231, BT–549 and SUM159PT cells. The IC_50_ was calculated. Data represents triplicate determinations.

The newly synthesized pyrimidines were examined for inhibition of cell viability of human breast cancer (MCF–7) cells (Table 1 and Table 2). Tamoxifen and doxorubicin were used as internal standards and produced a loss of viability of MCF–7 cells, with IC_50_ values of 2.96 and 1.84 µM, respectively. Among the oxazine-clubbed pyrimidine compounds, **3a** and **3g** produced an IC_50_ of 9.17 and 13.87 μM, respectively. Among piperazines, clubbed pyrimidine compounds **5a** and **5m** exhibited IC_50_ of 6.29 and 14.58 μM. All other derivative IC_50_ values were observed from 17.26 to >100 μM (Figure 3A) (see supplementary file). Among compounds **3**(**a**–**g**)**,** 4–methoxyphenyl-substituted oxazines (**I**) were observed to be active compared to 4–chlorophenyl and phenyl-substituted oxazines, whereas in compounds **5**(**a**–**p**), 4–methoxybenzyl-substituted pyrimidine were more potent than other benzylated derivatives. Also, 5–bromopyridine-substituted piperazines were found to be active, whereas the other piperazines were inactive. Lead molecules **3a** and **5b** were evaluated against MDA–MB–231, BT–549, and SUM159PT cells (Figure 3B) (Table 3). Among the two oxazine–pyrimidine derivatives, **5b** was more potent, with IC_50_ of 7.34, 5.98, and 14.81 µM. 

### 3.3. Title Compounds Induce Apoptosis in MCF–7 Cells

**Figure 4:**
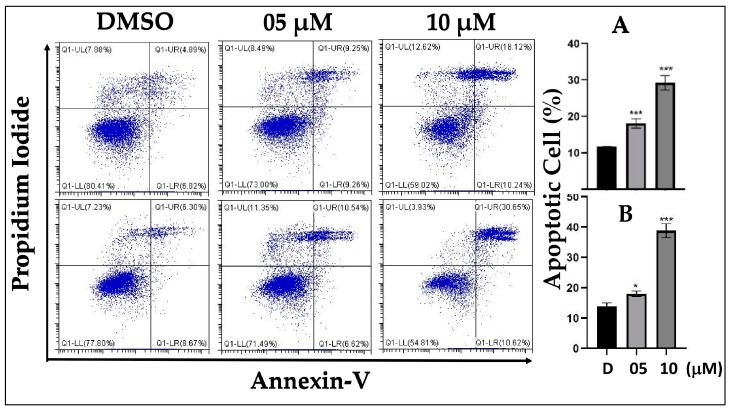
Evaluation of apoptosis in MCF-7 cells induced by lead compounds (**5b**, **A** and **3a**, **B**) at 5 and 10 μM for 24 h. Control MCF–7 cells or MCF–7 cells treated with lead compounds (**5b**, **A** and **3a**, **B**) were stained with Annexin V–AbFluor™ 488/PI Apoptosis Detection (Abbkine, KTA0002, Wuhan, China) followed by flow cytometry analysis. The percentage of apoptotic cells is indicated. Significant changes from the control group are shown by (* *p* < 0.05, *** *p* < 0.01) of three independent assays.

We previously described the discovery of 1,2 oxazines as anti-cancer drugs [32], along with their roles in triggering apoptosis, significantly increasing the population of sub-G1 cells and inhibiting the capacity of NF–κB to bind DNA in HCC cells. We therefore used MCF-7 cells to determine the effect of the lead compounds in Figure 4 on apoptosis. Examination of the data showed that the lead compounds stimulated dose-dependent apoptosis of MCF–7 cells (Figure 4).

### 3.4. Lead Compounds Arrest MCF–7 Cell Cycle at the Sub-G1 Phase

**Figure 5:**
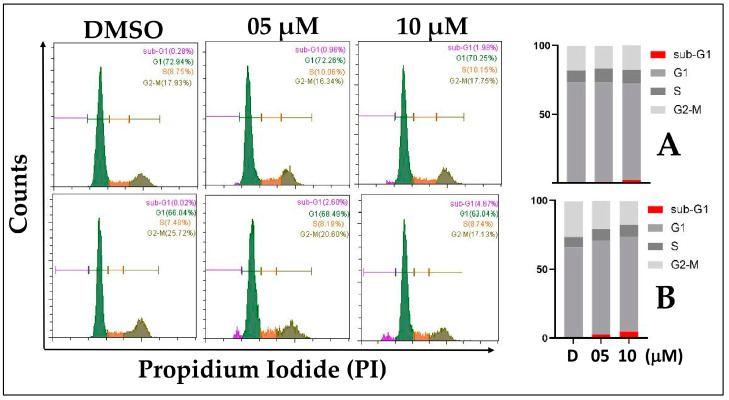
Flow cytometry analyzed MCF–7 cell cycle proportions. Representative cell cycle distribution histograms showing apoptosis in MCF–7 cells treated for 72 h with lead compounds such as **A** (**5b**) and **B** (**3a**) at concentrations of 5 and 10 mM, and analysis of the number of cells at each cell cycle stage with all phases.

We next investigated whether the lead compounds can hinder specific cell cycle progression. Propidium iodide labeling was used for the flow cytometric study of untreated and treated (lead compounds) MCF–7 cells. Lead compounds increased the proportion of cells in the sub-G1 phase relative to untreated cells [42] (Figure 5). 

### 3.5. Lead Compounds Inhibited the Phosphorylation of Human p65 Protein (Serine–536 Amino Acid) of NF–κB Subunit in MCF–7 Cells

**Figure 6:**
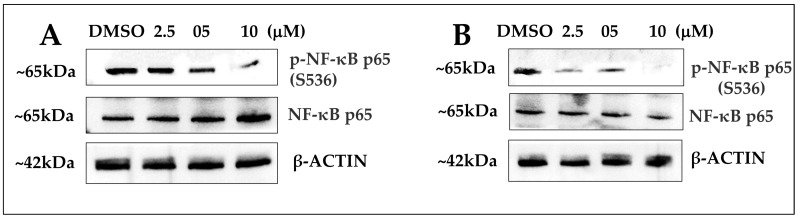
Effects of lead compounds **5b** (**A**) and **3a** (**B**) on the NF–κB signaling pathway in MCF–7 cells. Cells were treated for 24 h with lead compounds (0–10 μM). Western blotting evaluated p65 expression and phospho–p65 levels.

NF–κB activation is regulated by the enzyme inhibitor of κB (IκB) and kinase (IKK), which phosphorylates subunit p65 at serine 536, and which inhibits the NF–κB signaling pathway. Western blot analysis was used to examine if the lead compounds impacted the expression of the p65 protein or the levels of phospho–p65 in MCF–7 cells. Lead compounds **5b** and **3a**, as shown in Figure 6A,B, produce a concentration-dependent decrease of phospho–p65 levels relative to p65 protein expression in MCF-7 cells 24 h after treatment. 

### 3.6. In Silico Analysis of Novel Compounds ***3a*** and ***5b*** Targeting the NF–Kappa B p65 Subunit

**Figure 7:**
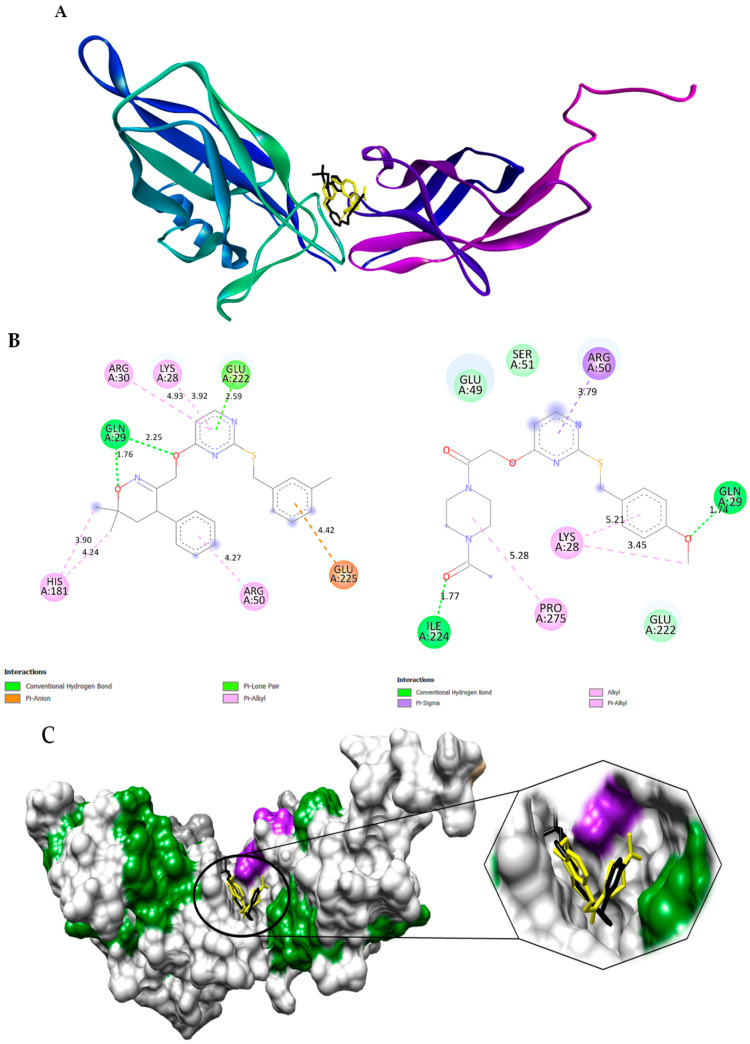
(**A**) Cartoon representation of both docked compounds **3a** (Black) and **5b** (Yellow) inside the binding pocket of the p65 subunit of NF–κB; (**B**) 2D interactions of compound **3a** and **5b** with the walls of the binding pocket, respectively andshowing bond distance (Å) between respective aminoacid and ligands. Pyrimidine ring of **3a** and 4–methoxybenzylthiol group of **5b** showed pi–alkyl interaction with Lys28 of p65 subunit of NF–κB; (**C**) Representation of three-dimensional surface view of docked compounds (**3a**: Black, **5b**: Yellow) inside the grove of NF–κB and its enlarged view for better understandings.

In this study, we performed in silico analysis to evaluate the binding energies and critical interactions of two novel compounds, **3a** or **5b**, targeting p65, the active site of NF–κB. Initially, the NF–κB structure was retrieved from the Protein Data Bank (PDB ID: 1IKN) and further used for molecular docking simulations using AutoDock4 tools. Molecular docking simulation revealed that novel compound **3a** demonstrated a binding energy of −9.32 kcal/mol, indicating a strong binding affinity for the active site of the NF–κB p65 subunit, while **5b** exhibited a binding energy of −7.32 kcal/mol, indicating a relatively weaker binding affinity. Further key interactions revealed that compound **3a** formed a hydrogen bond with the residue GLN–29. The hydrogen bond plays a crucial role in stabilizing the binding of **3a** to the active site. One π–anion bond and one π–lone pair bond formed with the residues GLU–225 and GLU–222, respectively. Additionally, hydrophobic interactions (π–alkyl) were observed between **3a** and specific residues like LYS–28, ARG–30, ARG–50, and HIS–181 in the binding pocket, contributing to its overall binding affinity. In comparison, **5b** engaged in hydrogen bonding interactions with residues GLN–29 and ILE–224 within the active site. These hydrogen bonds contribute to the binding stability of **5b**. One π–sigma bond formed with the residue ARG–50. Furthermore, hydrophobic interactions were observed (LYS–28 and PRO–275), further enhancing the binding of **5b** to the target protein (Figure 7B). The results of the docking study revealed that both **3a** and **5b** have potential as inhibitors of the NF–kappa B p65 subunit.

## 4. Discussion

Pyrimidines have been demonstrated to be effective inhibitors of NF–κB, and many of the pyrimidine-based drugs such as ibudilast, spebrutinib, and dasatinib are also reported as inhibitors of the NF–κB pathway. In the present work, we have designed and synthesized a new series of oxazine- and piperazine-clubbed pyrimidine derivatives as novel inhibitors of NF–κB. Loss of cell viability in MCF-7 cells revealed **3a** and **5b** to be the most potent among the series. Further efficacy of the lead compounds was studied by apoptosis and cell cycle assays, and Western blot analysis. The lead compounds increased the proportion of cells in the sub-G1 phase relative to untreated cells and induced apoptosis in MCF-7 cells. Lead molecules **5b** and **3a** produced a concentration-dependent decrease of phospho–p65 levels in MCF–7 cells. Additionally, an in silico docking study of lead compounds also supported the above data by prediction of strong binding to the p65 subunit of NF–κB.

## 5. Conclusions

In this study, novel compounds consisting of oxazines and piperazines linked to pyrimidines were synthesized and evaluated in MCF–7 breast cancer cells. Compounds **3a** and **5b** exhibited IC_50_s of 9.17 and 6.29 µM, respectively. Through in silico investigation, it was determined that compounds **3a** and **5b** potentially bind to the active site of NF–κB. Subsequent biological assays confirmed that lead compounds **3a** and **5b** effectively inhibited NF–κBp65 phosphorylation in MCF–7 cells, presenting a promising chemical entity targeting NF–κB in breast cancer cells.

## Figures and Tables

**Table 1:** Efficacy of newly synthesized oxazine-tethered thiouracil derivatives in MCF–7 cells.

Entry	R_1_	R_2_	R_3_	Yield in %	IC_50_ in μM
**3a**	H	3Me	I	96	9.17
**3b**	H	3Me	II	96	>100
**3c**	H	3Me	III	95	22.68
**3d**	H	4Cl	I	94	23.53
**3e**	H	4Cl	II	91	>100
**3f**	H	4Cl	III	96	48.42
**3g**	H	4F	I	95	13.87
**3h**	H	4F	II	96	50.74
**3i**	H	4F	III	94	ND
**3j**	Me	4F	I	94	86.46
**3k**	Me	4F	II	96	ND
**3l**	Me	4F	III	97	ND
Doxorubicin				2.96	
Tamoxifen				1.84	

ND = Not Determined.

**Table 2:** Efficacy of newly synthesized piperazine-tethered thiouracil derivatives in MCF–7 cells.

Entry	R_1_	R_2_	R_4_	Yield in %	IC_50_ in μM
**5a**	H	4OMe	IV	95	16.38
**5b**	H	4OMe	V	90	6.29
**5c**	H	4OMe	VI	90	17.26
**5d**	H	4OMe	VIII	94	29.38
**5e**	H	3Me	V	90	>100
**5f**	H	3Me	VII	80	79.00
**5g**	H	3Me	VI	89	>100
**5h**	H	3Me	VIII	90	30.09
**5i**	H	4F	VIII	89	83.30
**5j**	H	4F	V	95	>100
**5k**	H	4F	VII	85	>100
**5l**	H	4F	VI	80	>100
**5m**	H	4Cl	VIII	90	14.58
**5n**	H	4Cl	V	88	>100
**5o**	H	4Cl	VII	92	42.00
**5p**	H	4Cl	VI	80	>100
Doxorubicin				2.96	
Tamoxifen				1.84	

**Table 3:** IC_50_ (µM) of lead molecules **3a** or **5b** in MDA–MB–231, BT–549, and SUM159PT cells.

Entry	MDA–MB–231	BT–549	SUM159PT
**3a**	7.34	5.98	14.81
**5b**	57.42	37.54	47.91

## Data Availability

All data are freely available with this article.

## Supplementary Materials

The following supporting information can be downloaded at: https://www.mdpi.com/article/10.3390/biomedicines11102716/s1. Figures S2–S23 and S24–S40 contain NMR, LCMS of **3**(**a**–**l**), **5**(**a**–**p**), and Figures S44–S50 contains IC_50_ values of newly synthesized compounds.
