# Supplementary material for: Development of Piperazine- and Oxazine-Linked Pyrimidines as p65 Subunit Binders of NF–κB in Human Breast Cancer Cells
Source: Biomedicines. 2023 Oct 6;11(10):2716. doi: 10.3390/biomedicines11102716 (PMC10604619; doi:10.3390/biomedicines11102716)

PAGES	CONTENT
**S2-S23**	^{1}H and ^{13}C NMR of **3**(**a-l**)
**S24-S40**	H and ^{13}C NMR of **5**(**a-p**)
**S41-S43**	IC_{50} of **3**(**a-l**) against MCF-7
**S44-S48**	IC_{50} of **5**(**a-p**) against MCF-7
**S49**	IC_{50} of **3a** against MDA-MB-231, BT-549, SUM159PT
**S50**	IC_{50} of **5b** against MDA-MB-231, BT-549, SUM159PT
**S51**	Original blots of western blot analysis

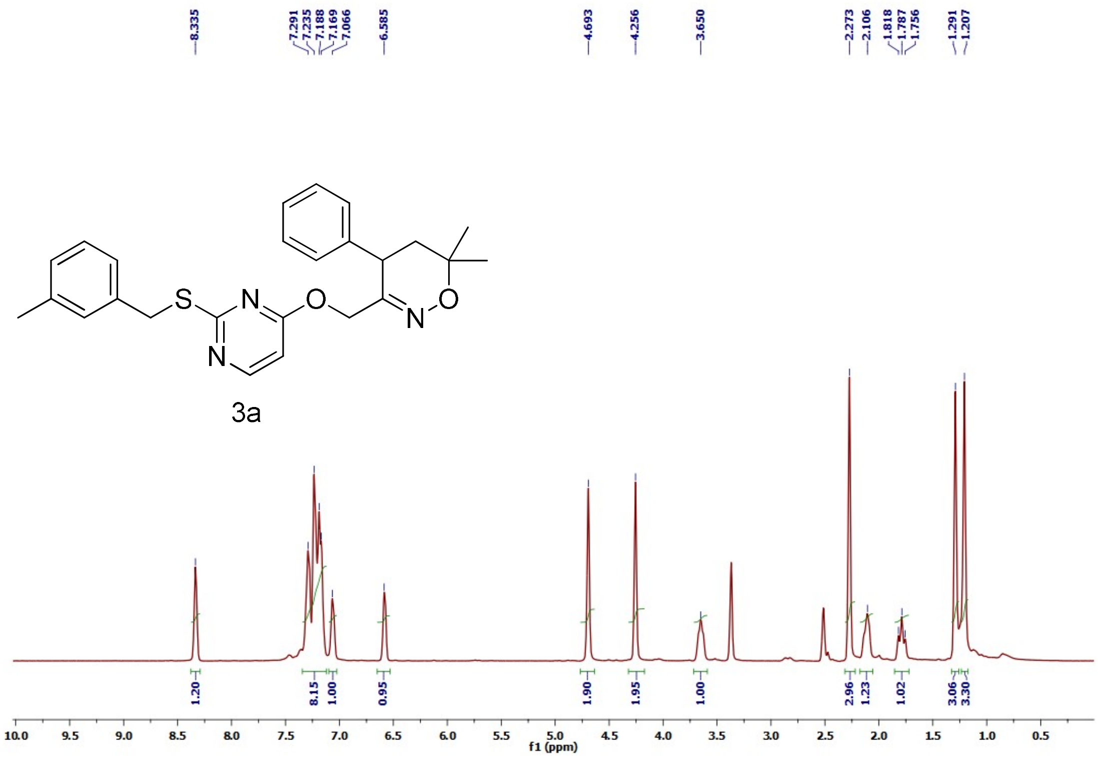

8.335
7.291
7.235
7.188
7.169
7.066
6.585
4.693
4.256
3.650
2.273
2.106
1.818
1.787
1.756
1.291
1.207
3a
1.20
8.15
1.00
0.95
1.90
1.95
1.00
2.96
1.23
1.02
3.06
3.30
10.0
9.5
9.0
8.5
8.0
7.5
7.0
6.5
6.0
5.5
5.0
4.5
4.0
3.5
3.0
2.5
2.0
1.5
1.0
0.5
f1 (ppm)

^{1}H NMR OF **3a**

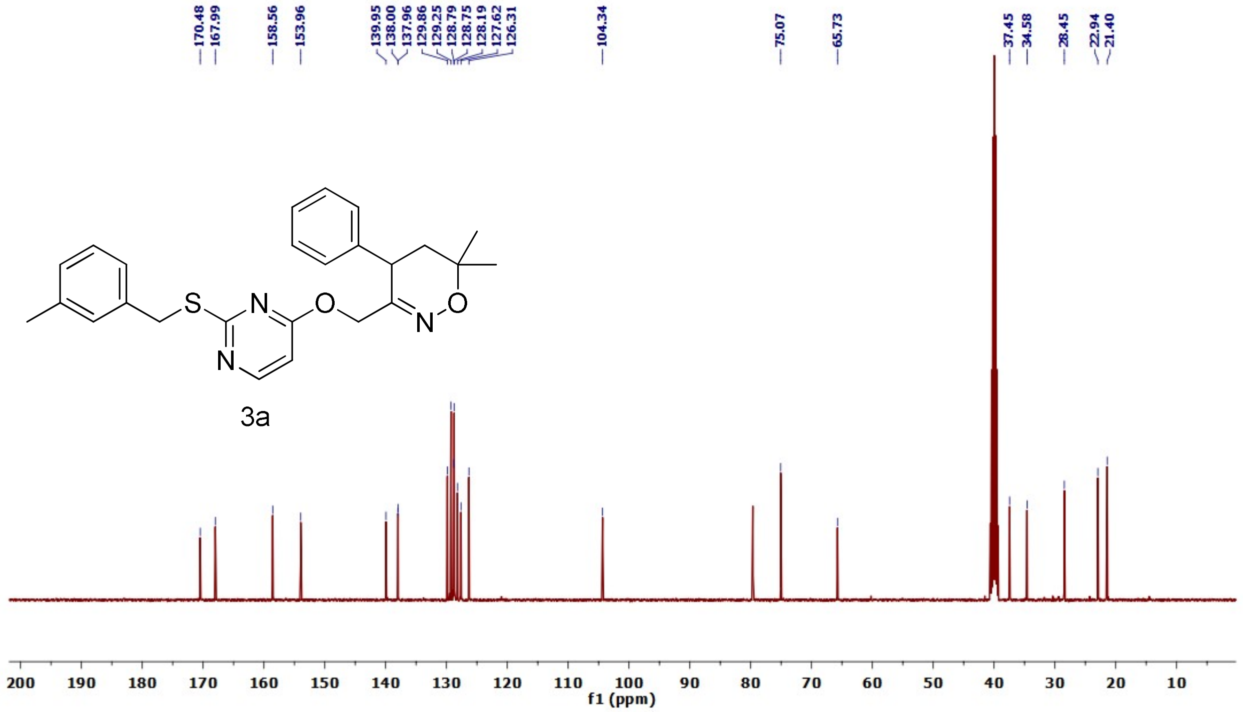

^{13}C NMR OF **3a**

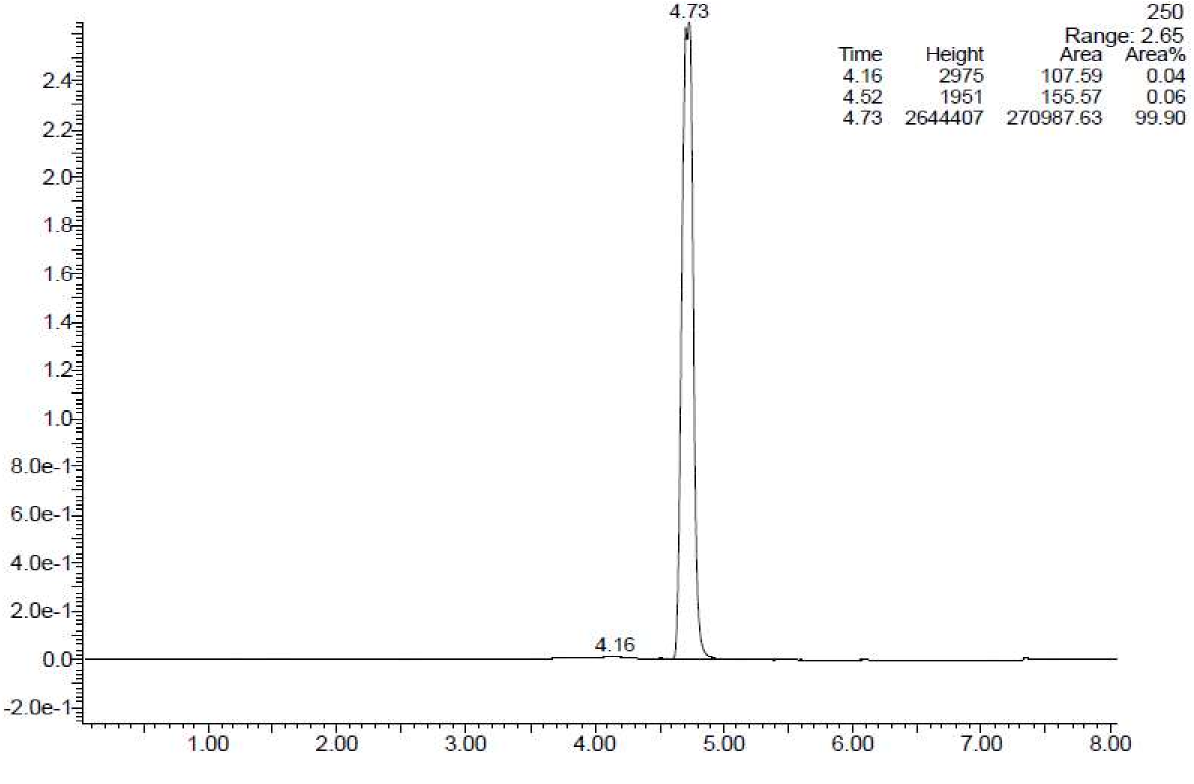

Liquid chromatogram of **3a**

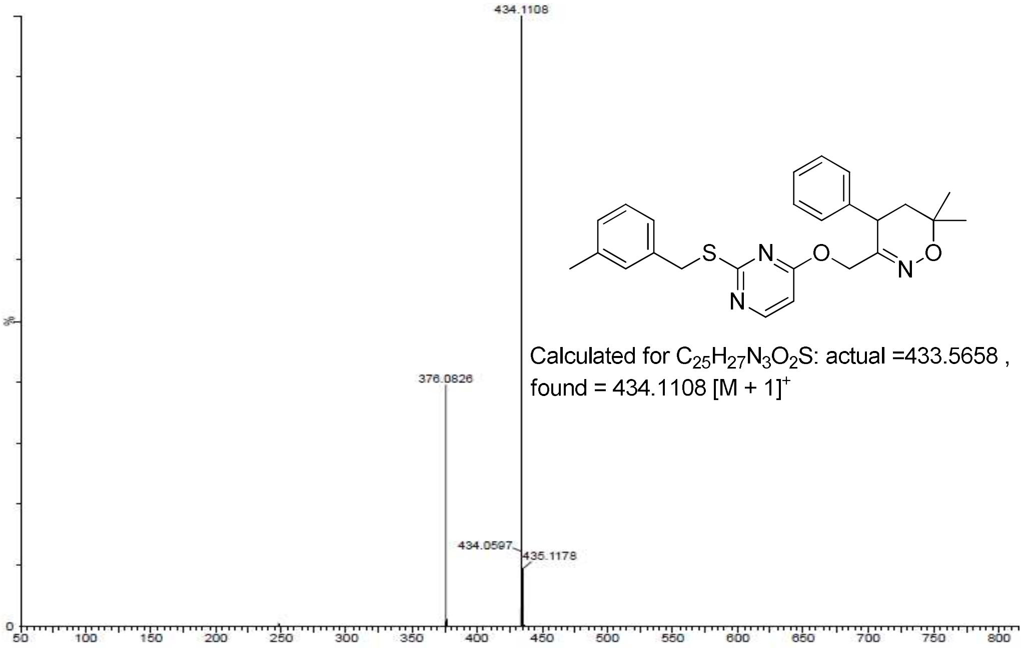

Mass spectra of **3a**

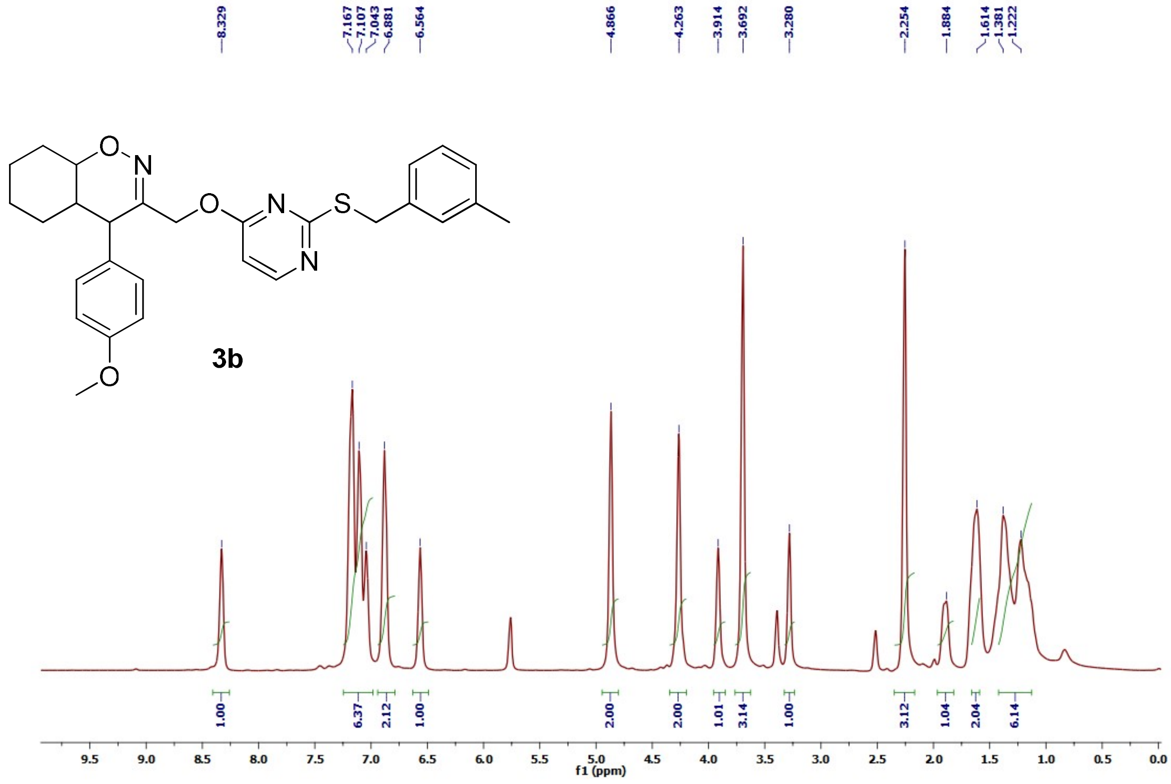

^{1}H NMR OF **3b**

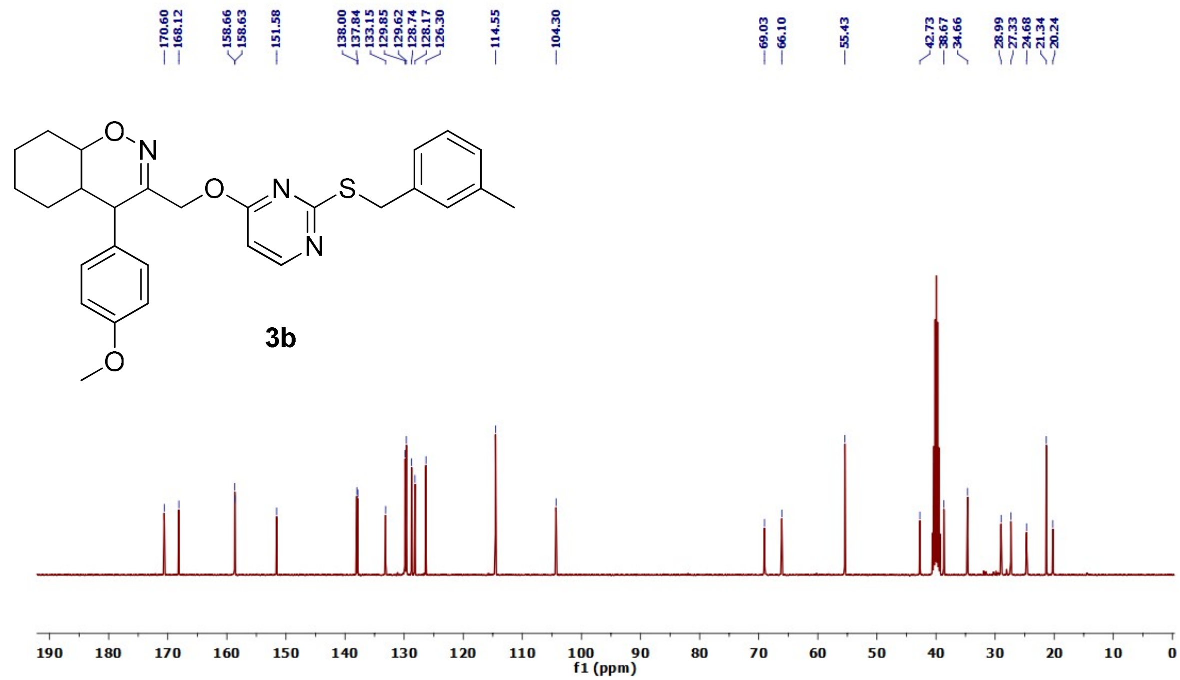

^{13}C NMR OF **3b**

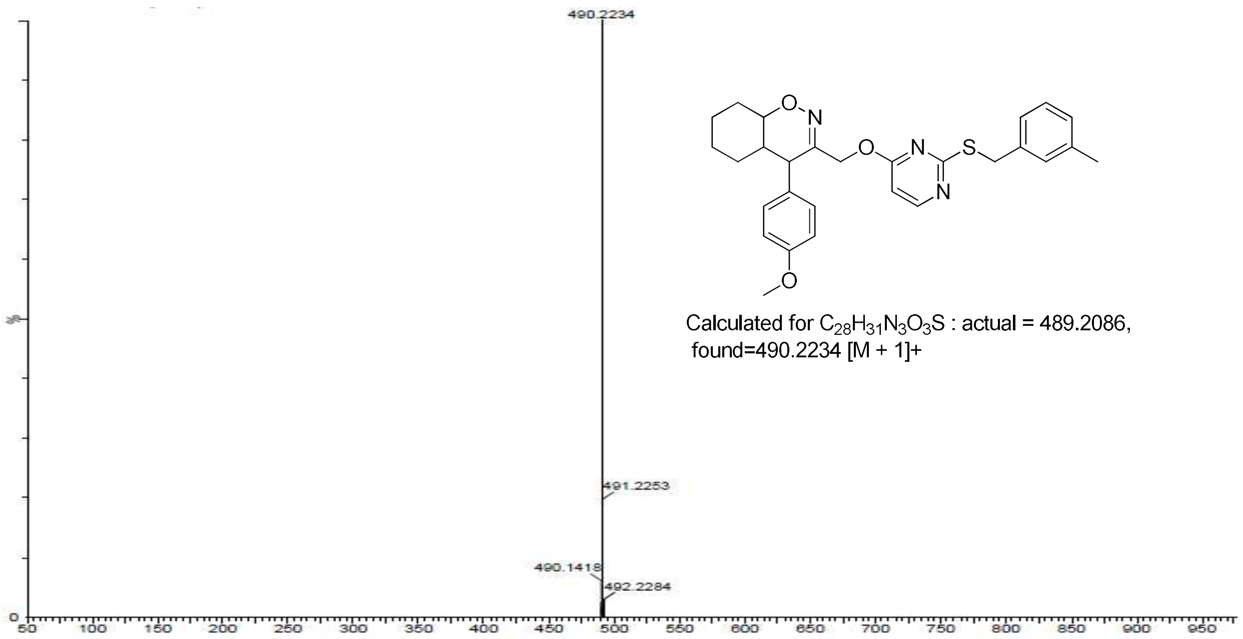

Mass spectra of **3b**

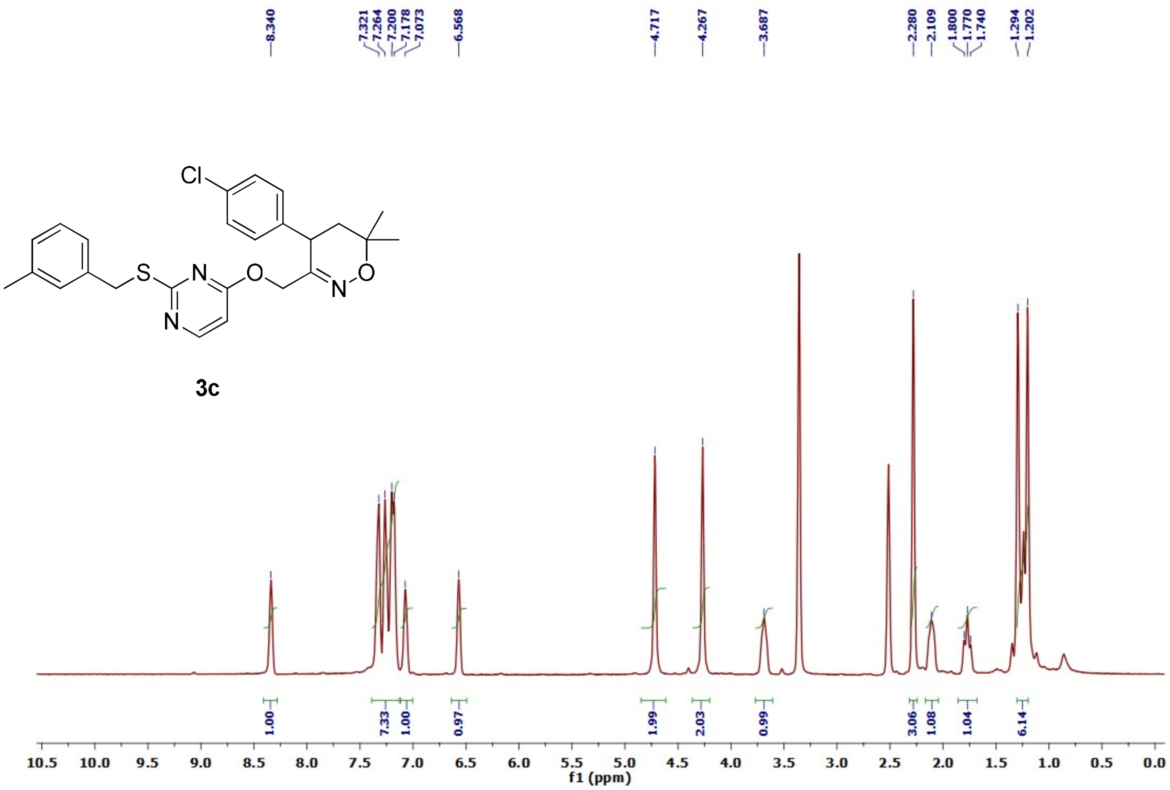

^{1}H NMR OF **3c**

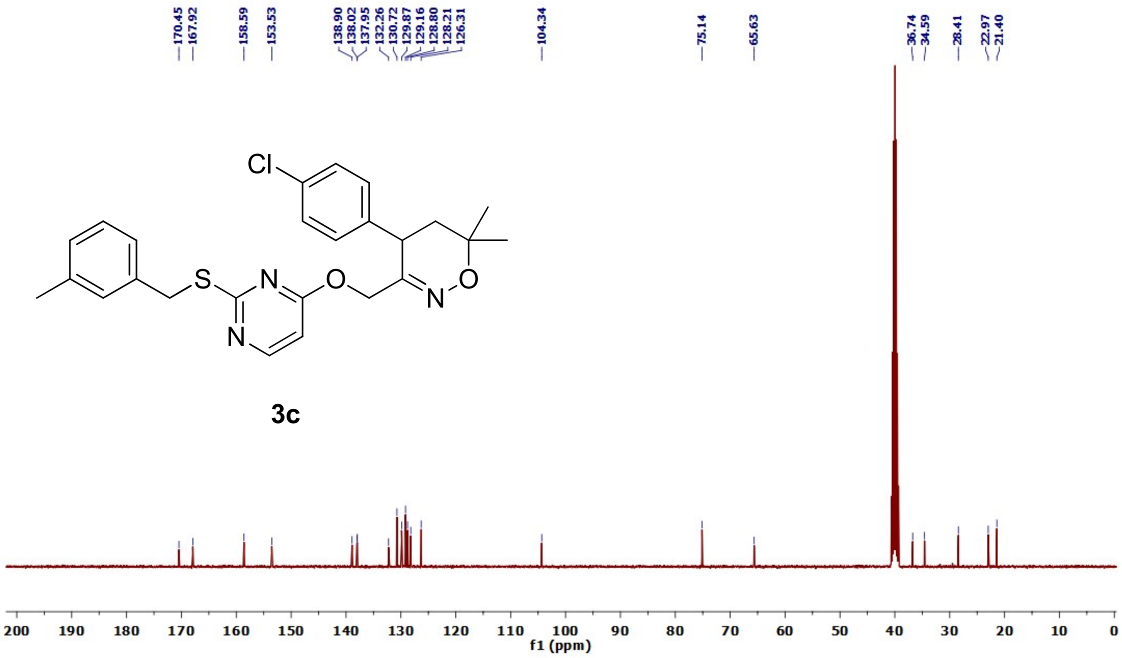

^{13}C NMR OF **3c**

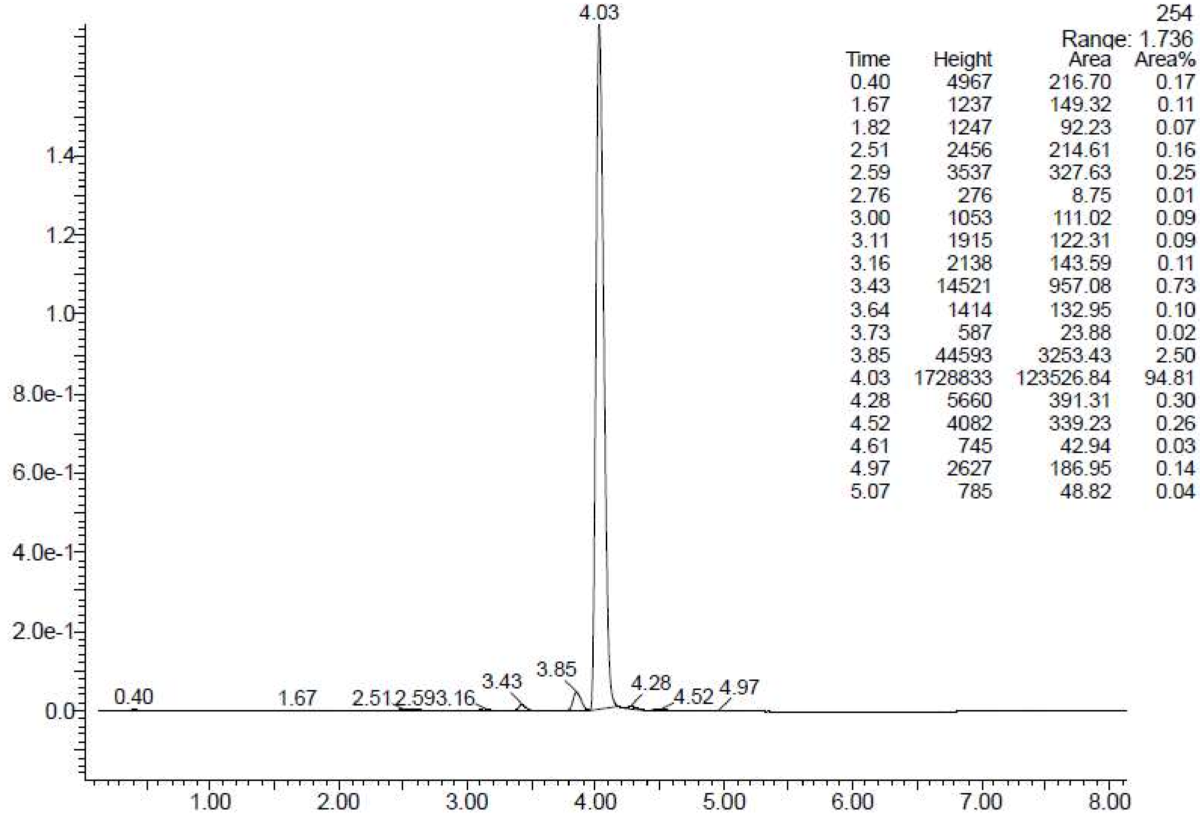

Time	Height	Area	Area%
0.40	4967	216.70	0.17
1.67	1237	149.32	0.11
1.82	1247	92.23	0.07
2.51	2456	214.61	0.16
2.59	3537	327.63	0.25
2.76	276	8.75	0.01
3.00	1053	111.02	0.09
3.11	1915	122.31	0.09
3.16	2138	143.59	0.11
3.43	14521	957.08	0.73
3.64	1414	132.95	0.10
3.73	587	23.88	0.02
3.85	44593	3253.43	2.50
4.03	1728833	123526.84	94.81
4.28	5660	391.31	0.30
4.52	4082	339.23	0.26
4.61	745	42.94	0.03
4.97	2627	186.95	0.14
5.07	785	48.82	0.04

Liquid chromatogram of TRX-03(**3c**)

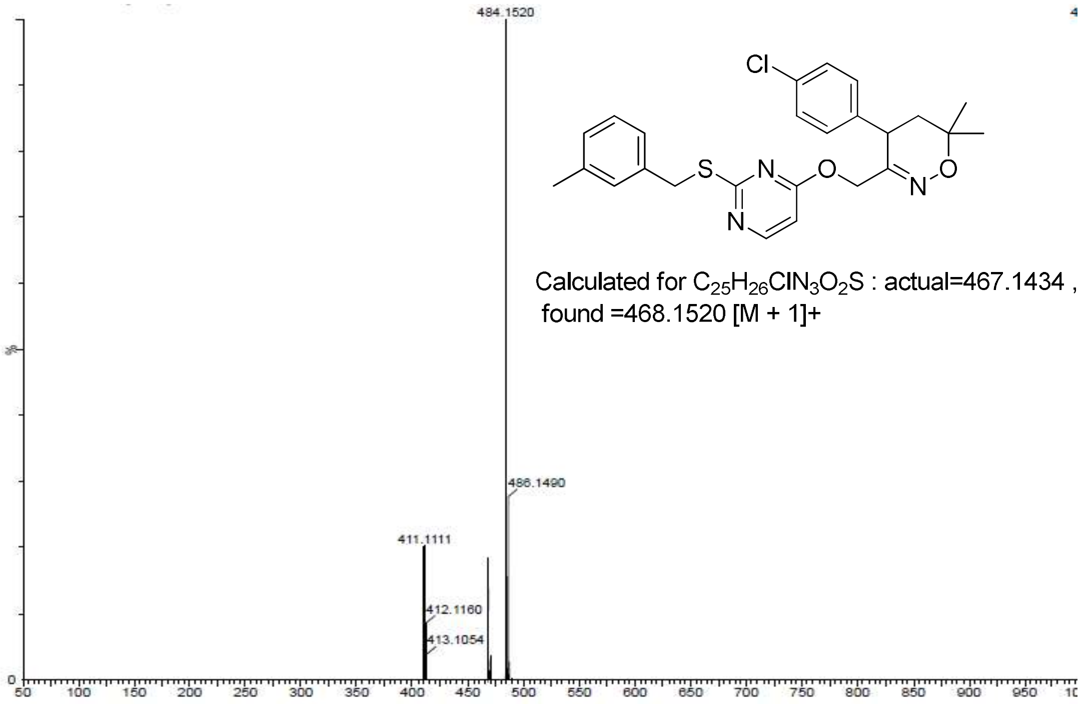

Calculated for $C_{25}H_{26}ClN_3O_2S$: actual=467.1434 , found =468.1520 [M + 1]+

Mass spectra of **3c**

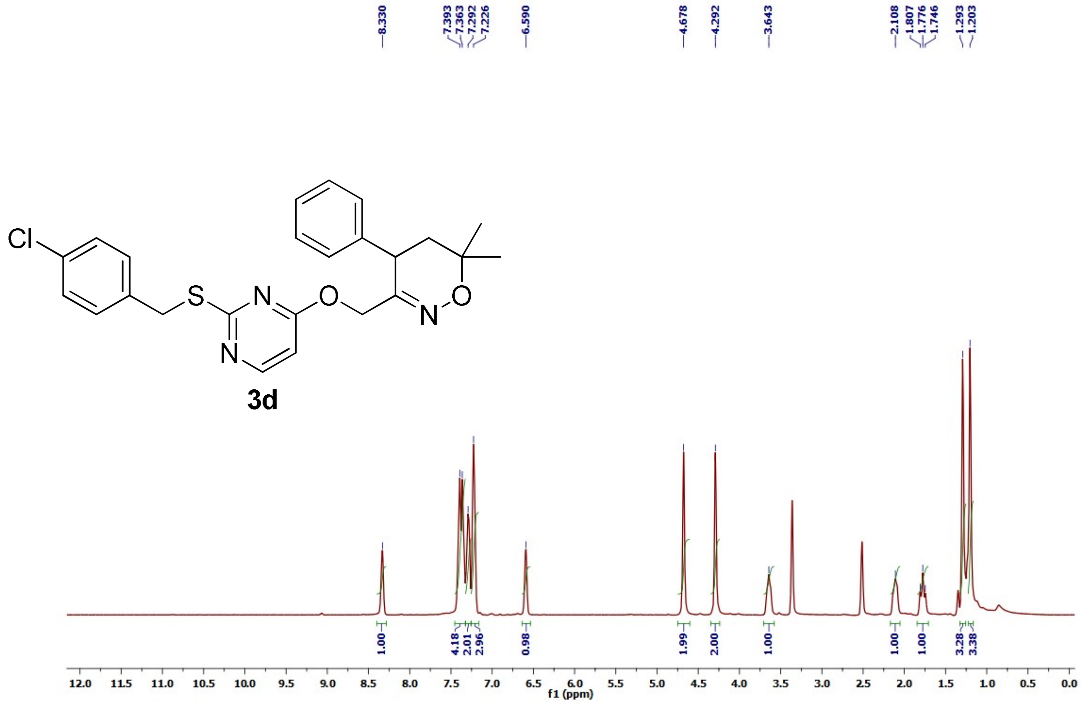

^{1}H NMR OF **3d**

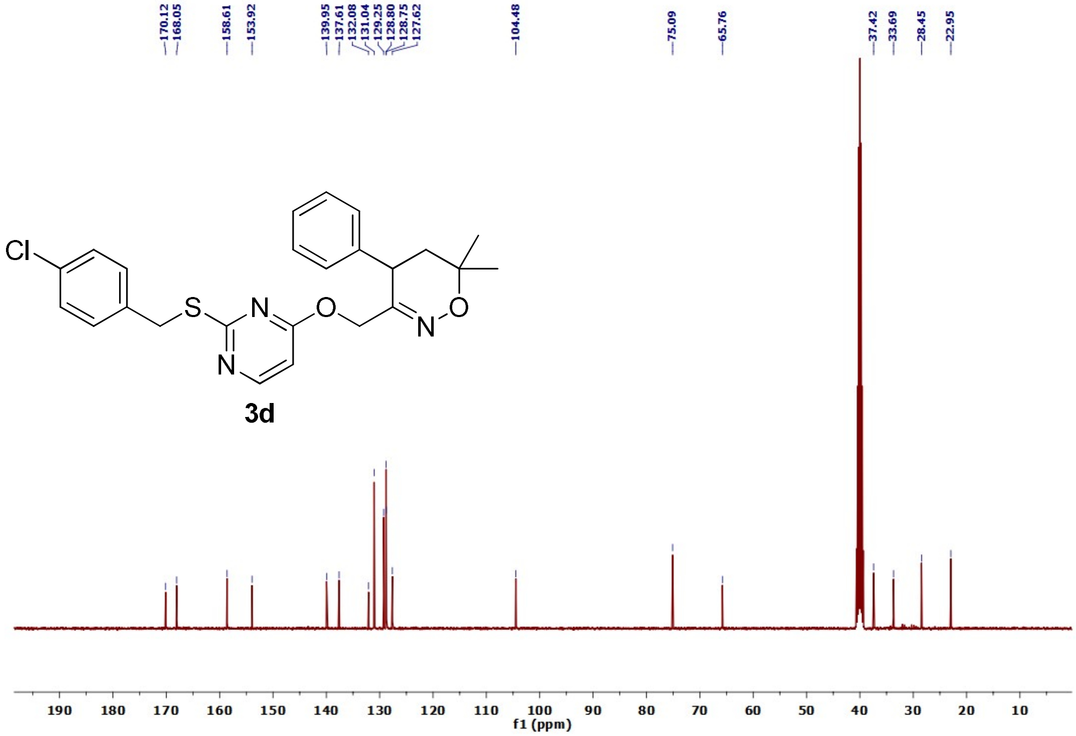

^{13}C NMR OF **3d**

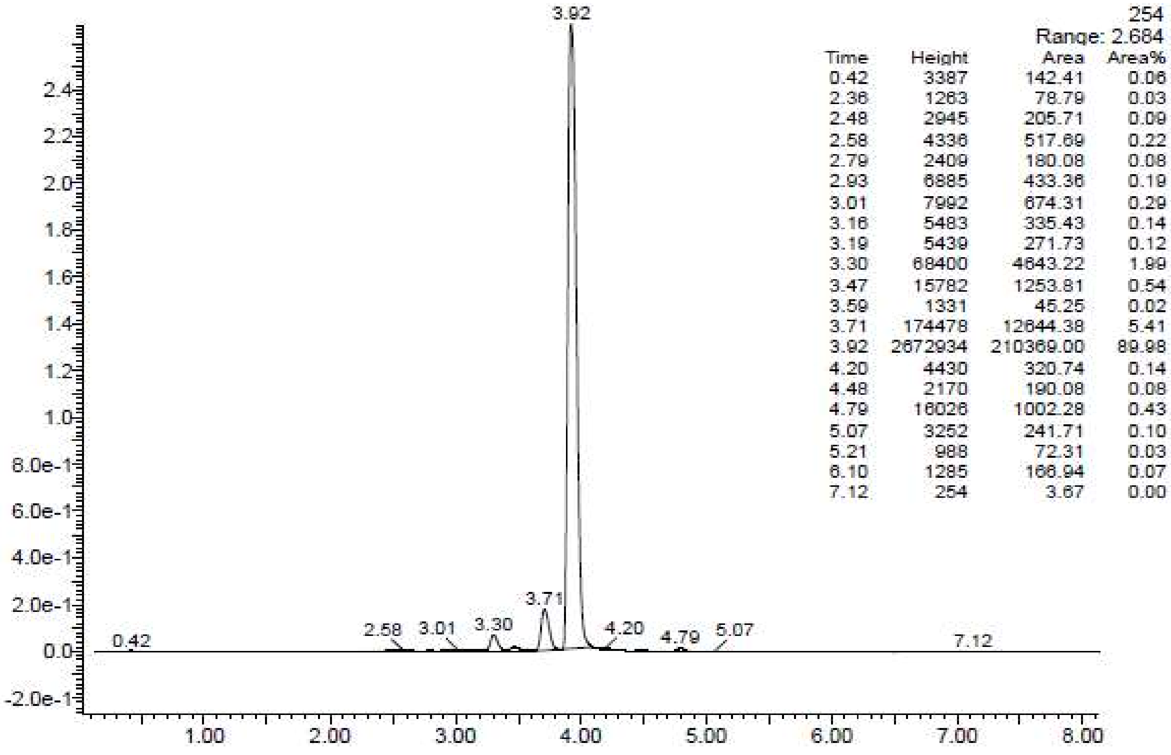

Liquid chromatogram of **3d**

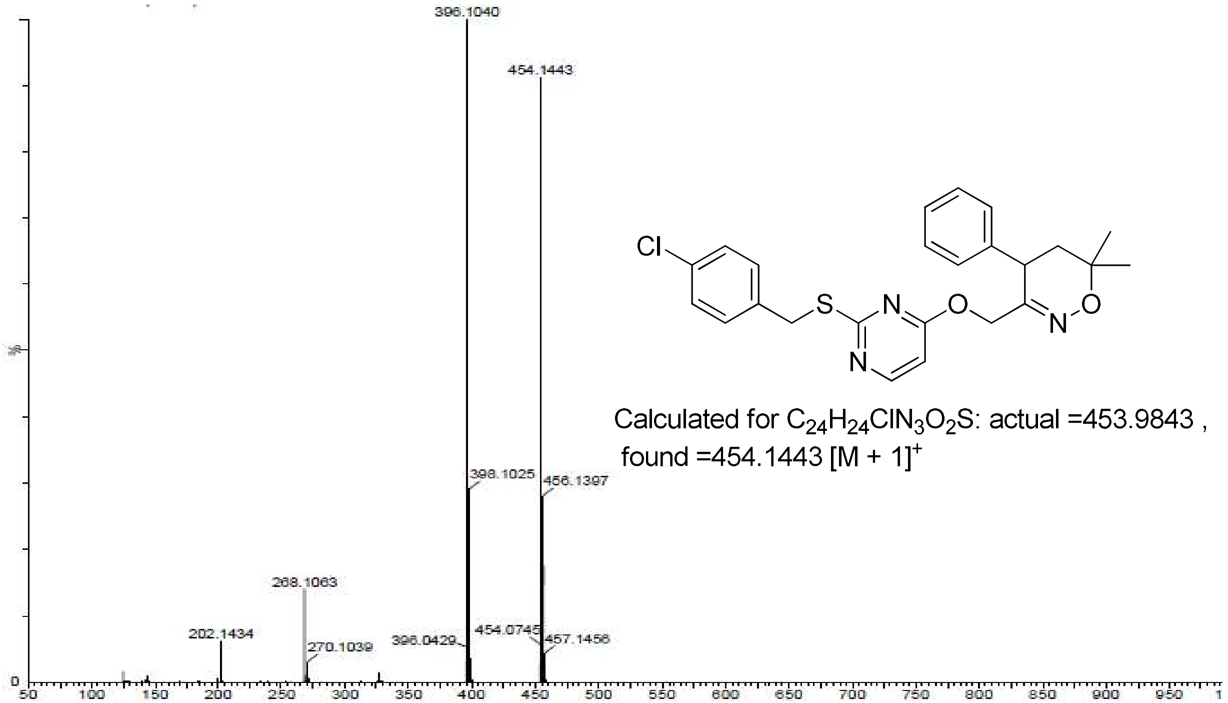

Mass spectra of **3d**

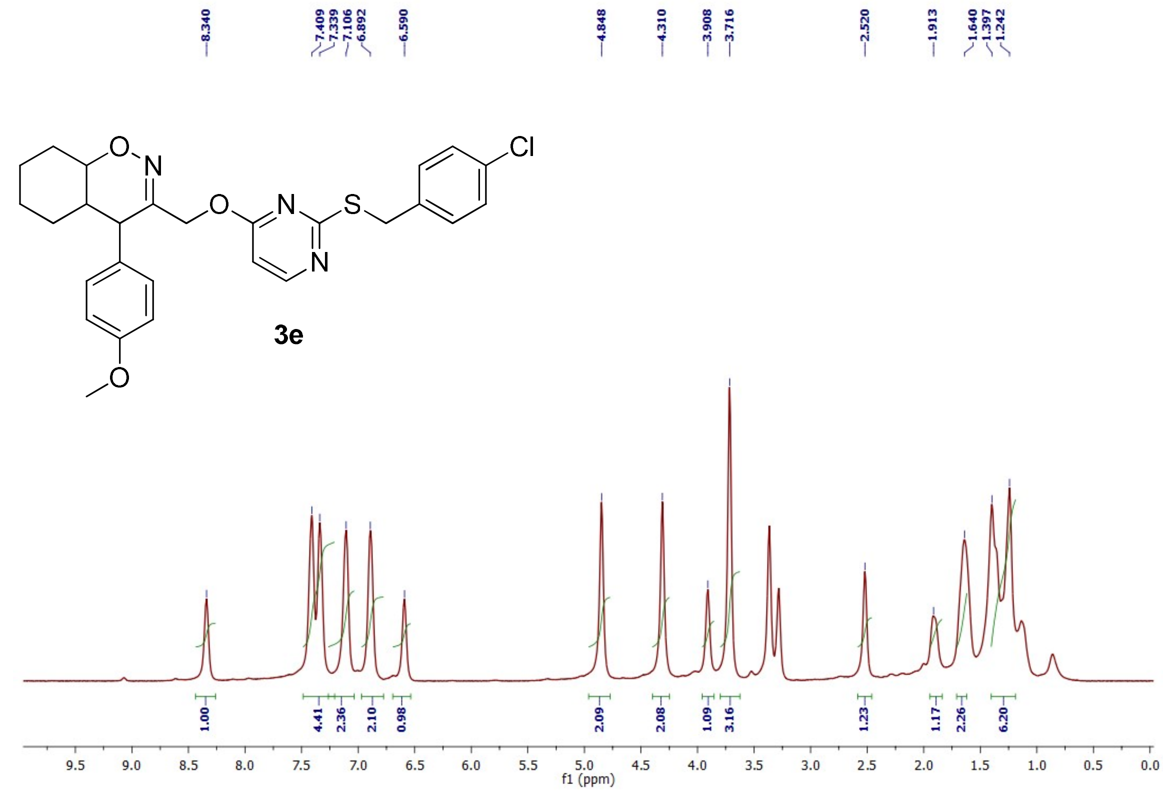

^{1}H NMR OF **3e**

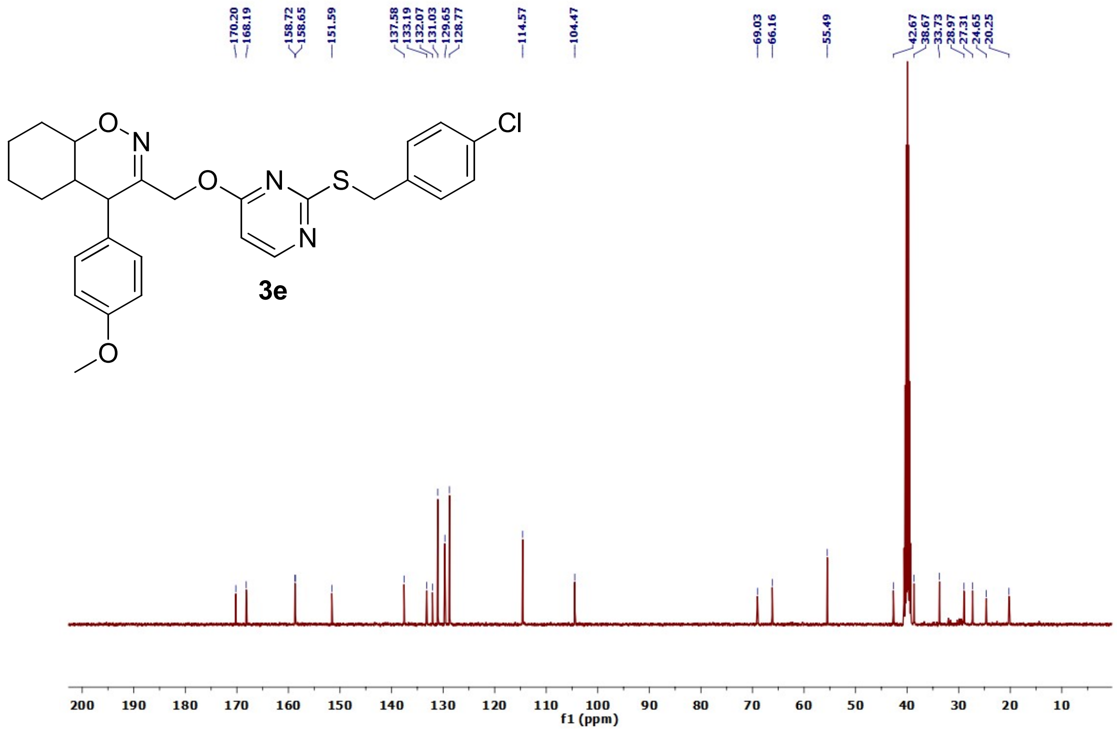

^{13}C NMR OF **3e**

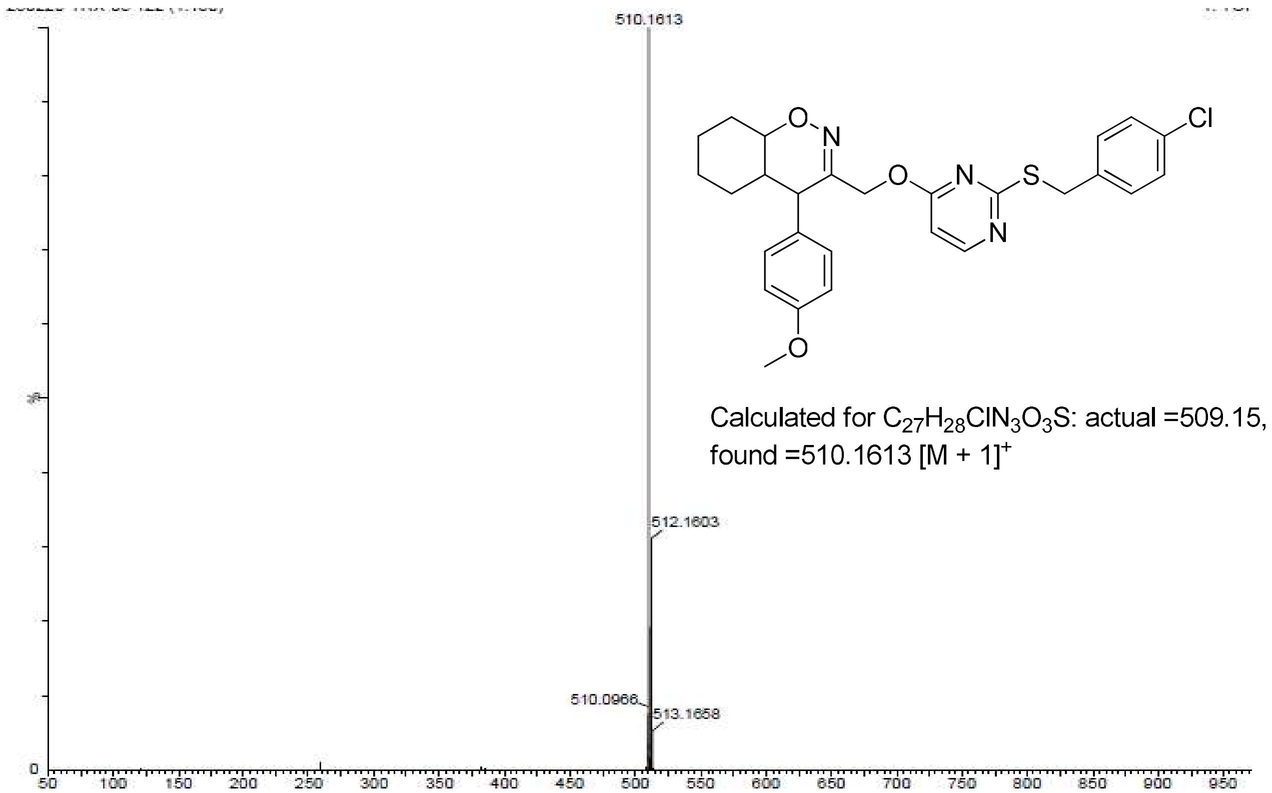

Mass spectra of **3e**

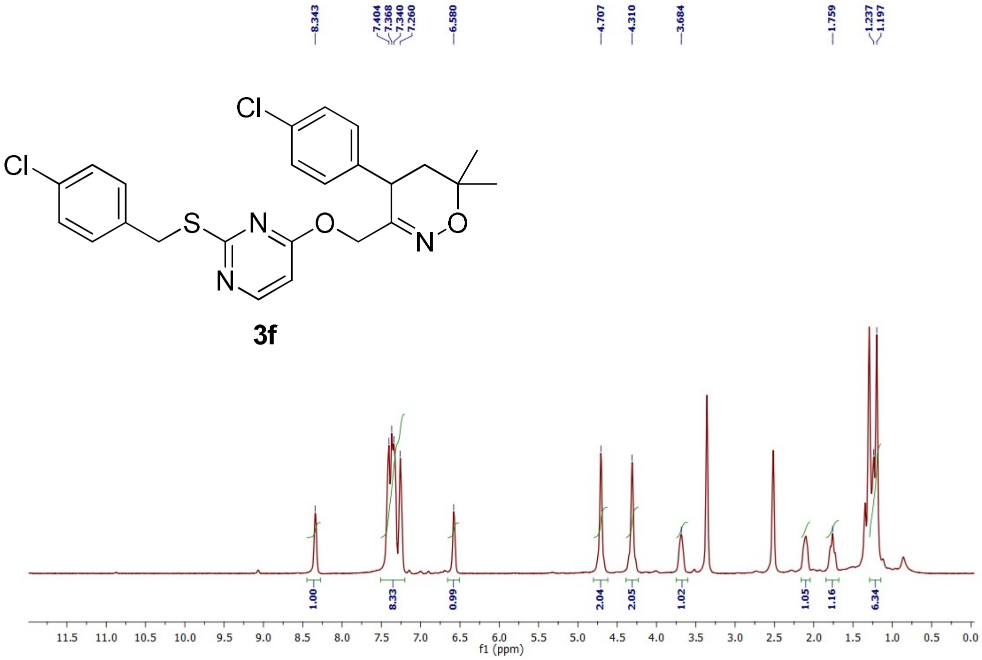

^{1}H NMR OF **3f**

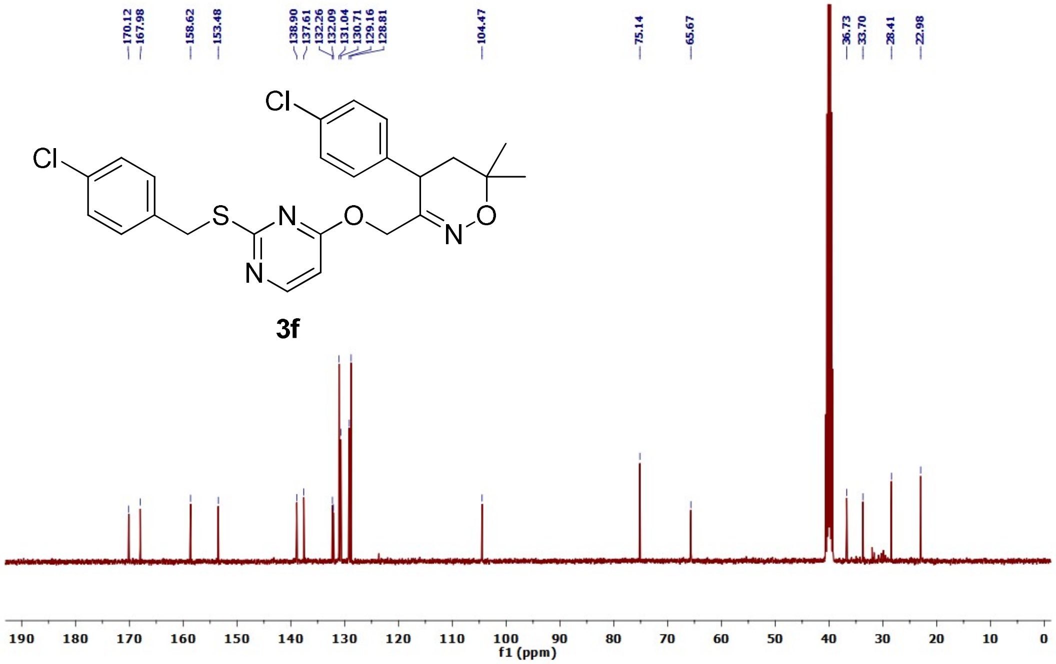

^{13}C NMR OF **3f**

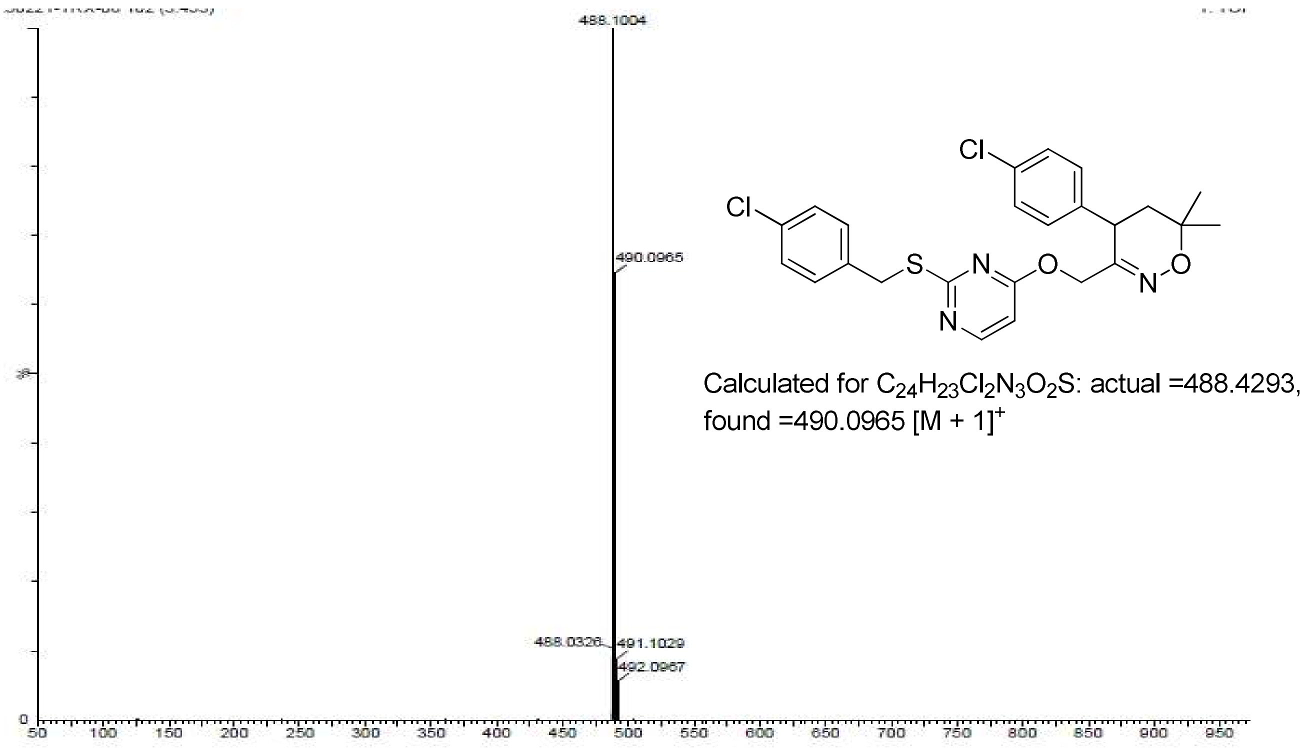

Mass spectra of TRX-05 (**3f**)

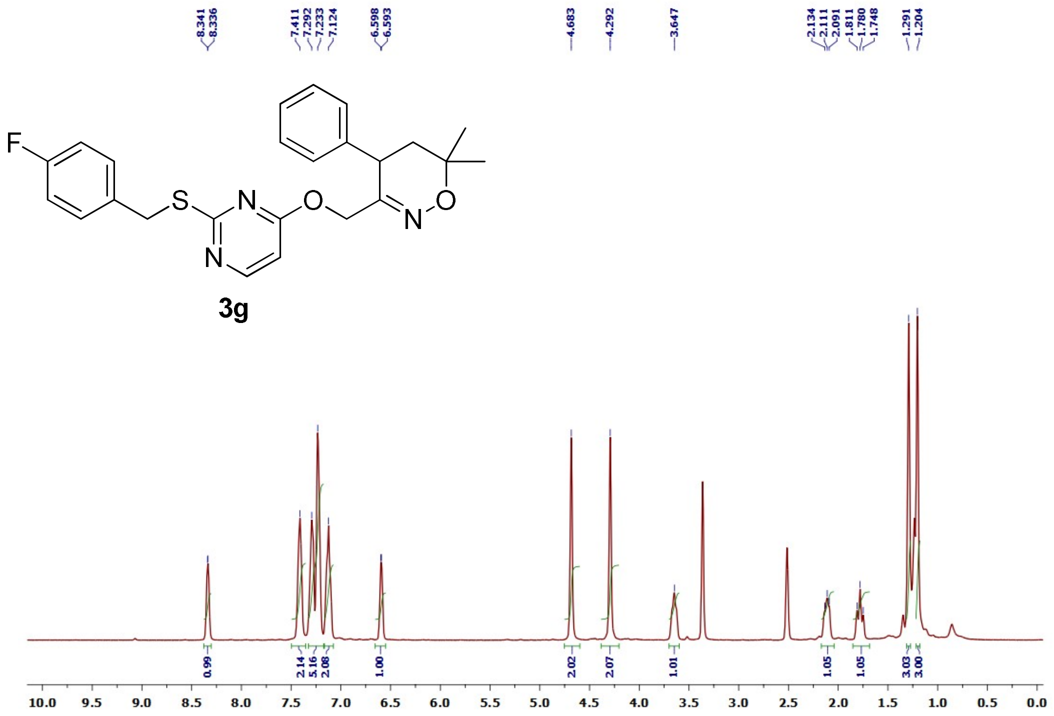

^{1}H NMR OF **3g**

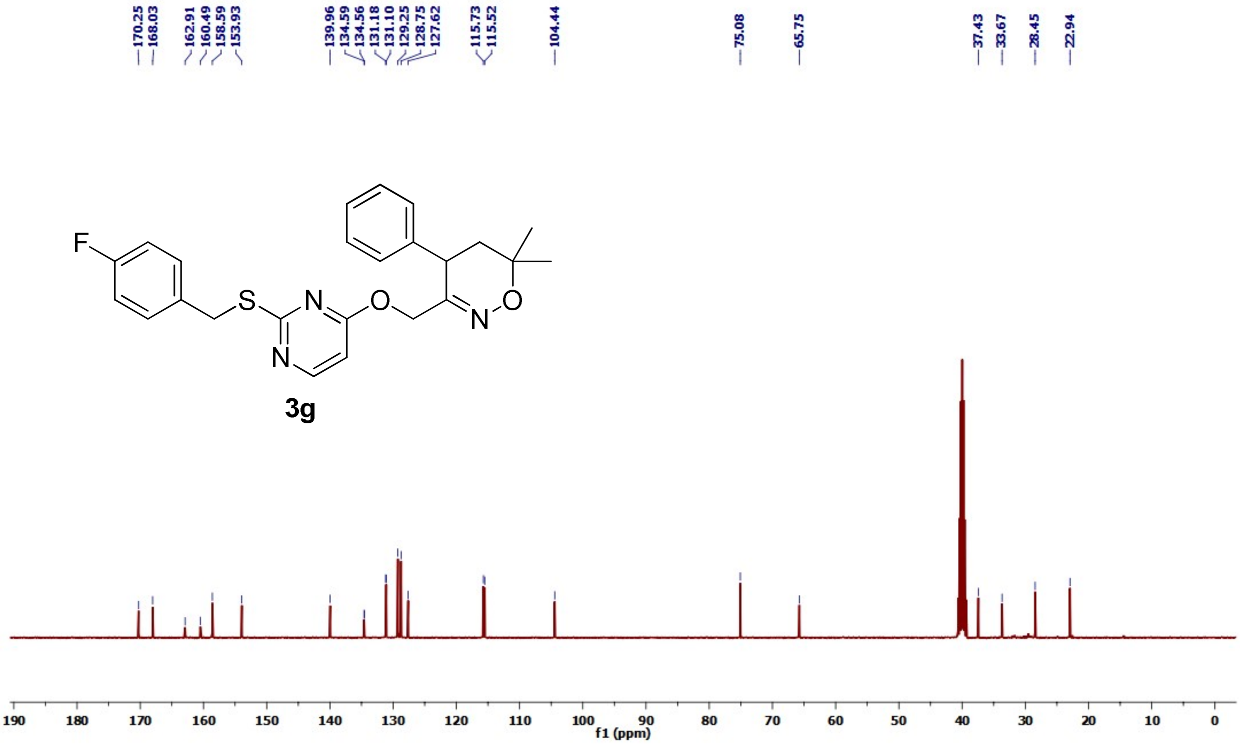

^{13}C NMR OF **3g**

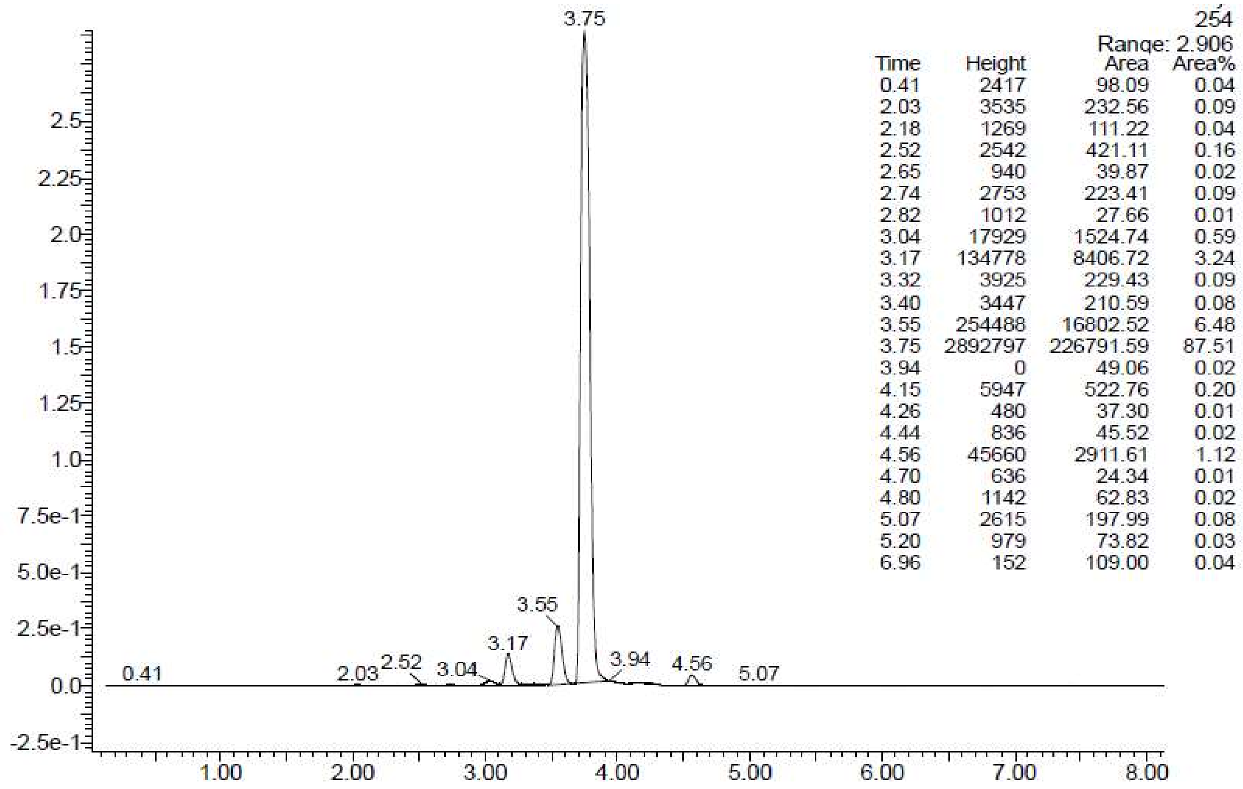

Time	Height	Area	Area%
0.41	2417	98.09	0.04
2.03	3535	232.56	0.09
2.18	1269	111.22	0.04
2.52	2542	421.11	0.16
2.65	940	39.87	0.02
2.74	2753	223.41	0.09
2.82	1012	27.66	0.01
3.04	17929	1524.74	0.59
3.17	134778	8406.72	3.24
3.32	3925	229.43	0.09
3.40	3447	210.59	0.08
3.55	254488	16802.52	6.48
3.75	2892797	226791.59	87.51
3.94	0	49.06	0.02
4.15	5947	522.76	0.20
4.26	480	37.30	0.01
4.44	836	45.52	0.02
4.56	45660	2911.61	1.12
4.70	636	24.34	0.01
4.80	1142	62.83	0.02
5.07	2615	197.99	0.08
5.20	979	73.82	0.03
6.96	152	109.00	0.04

Liquid chromatogram of **3g**

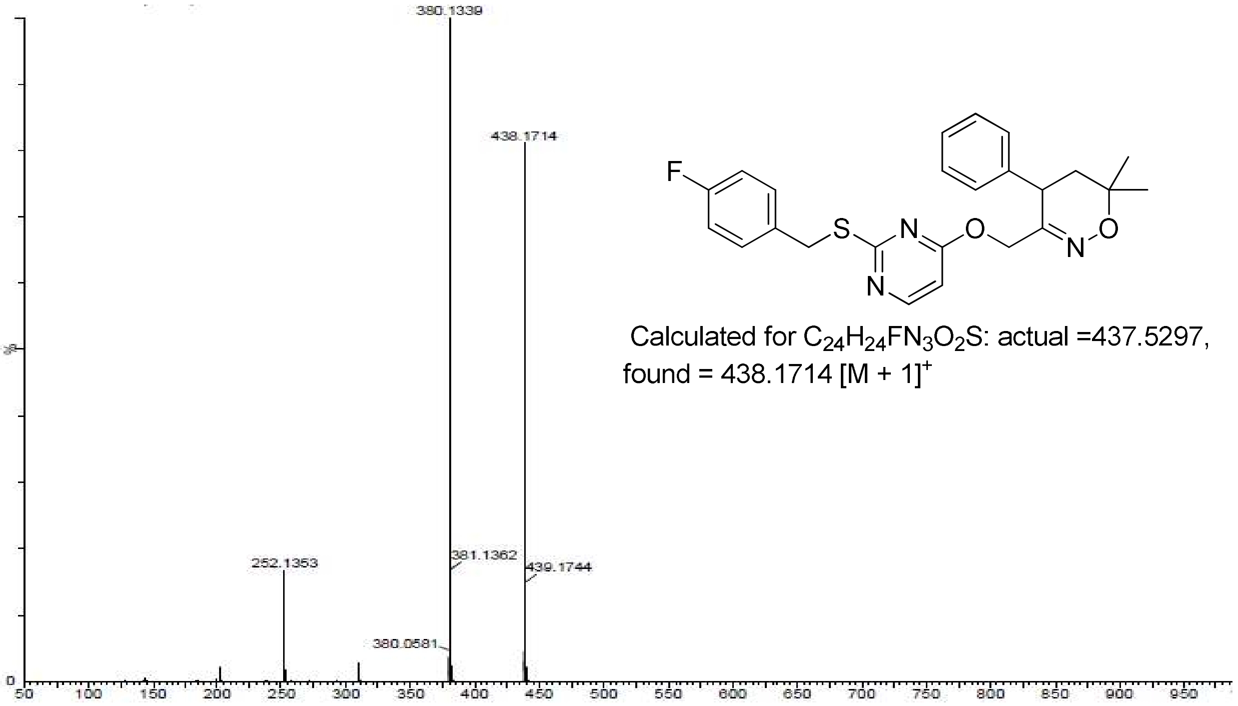

Calculated for $C_{24}H_{24}FN_3O_2S$: actual =437.5297, found = 438.1714 $[M + 1]^+$

Mass spectra of **3g**

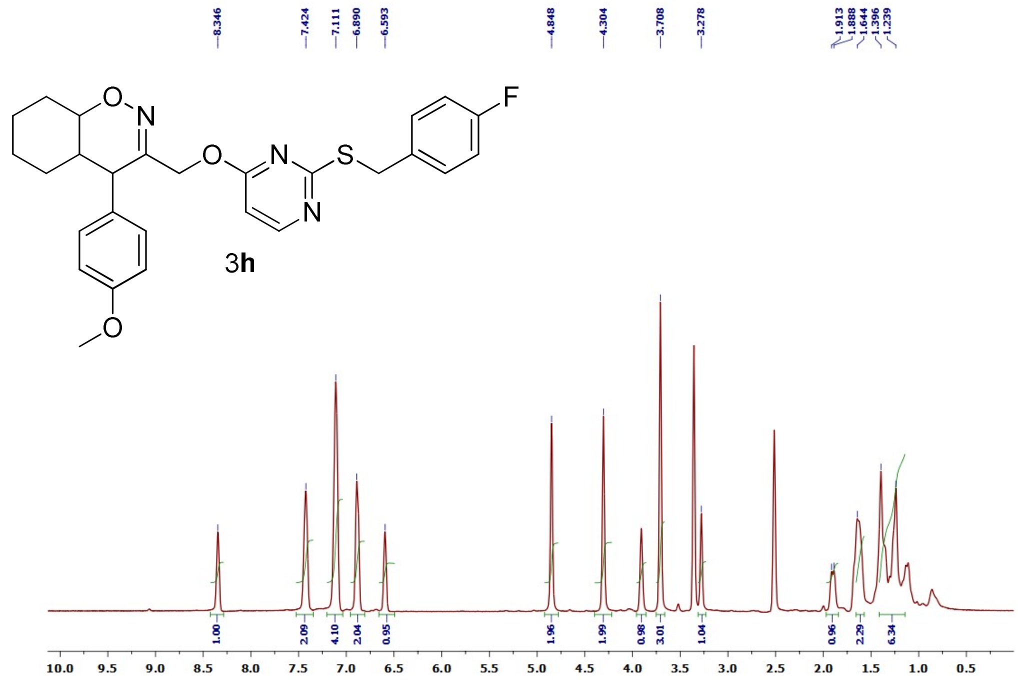

^{1}H NMR OF **3h**

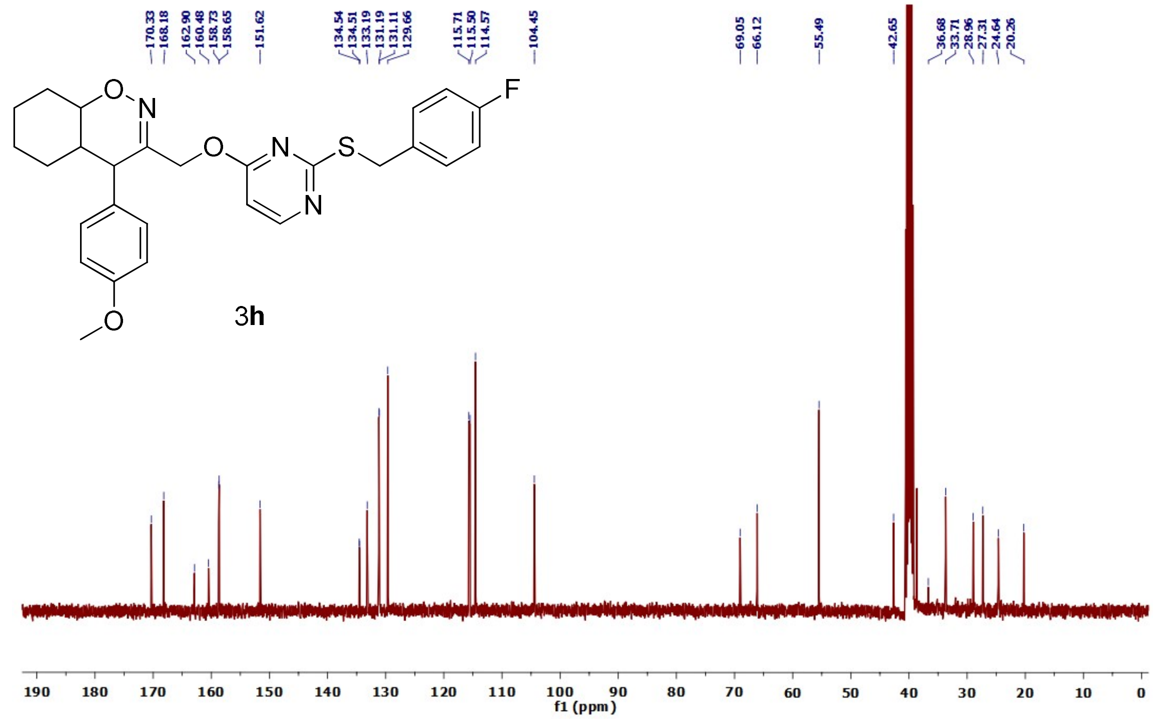

^{13} NMR OF **3h**

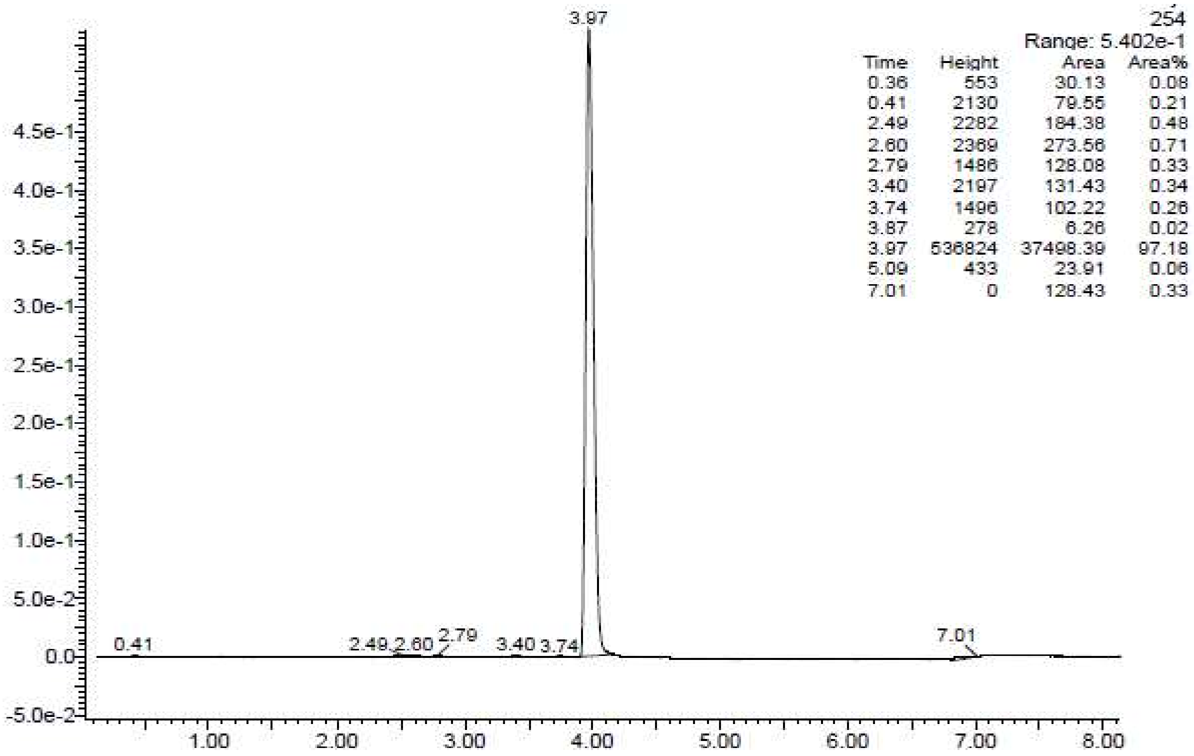

Liquid chromatogram of (**3h**)

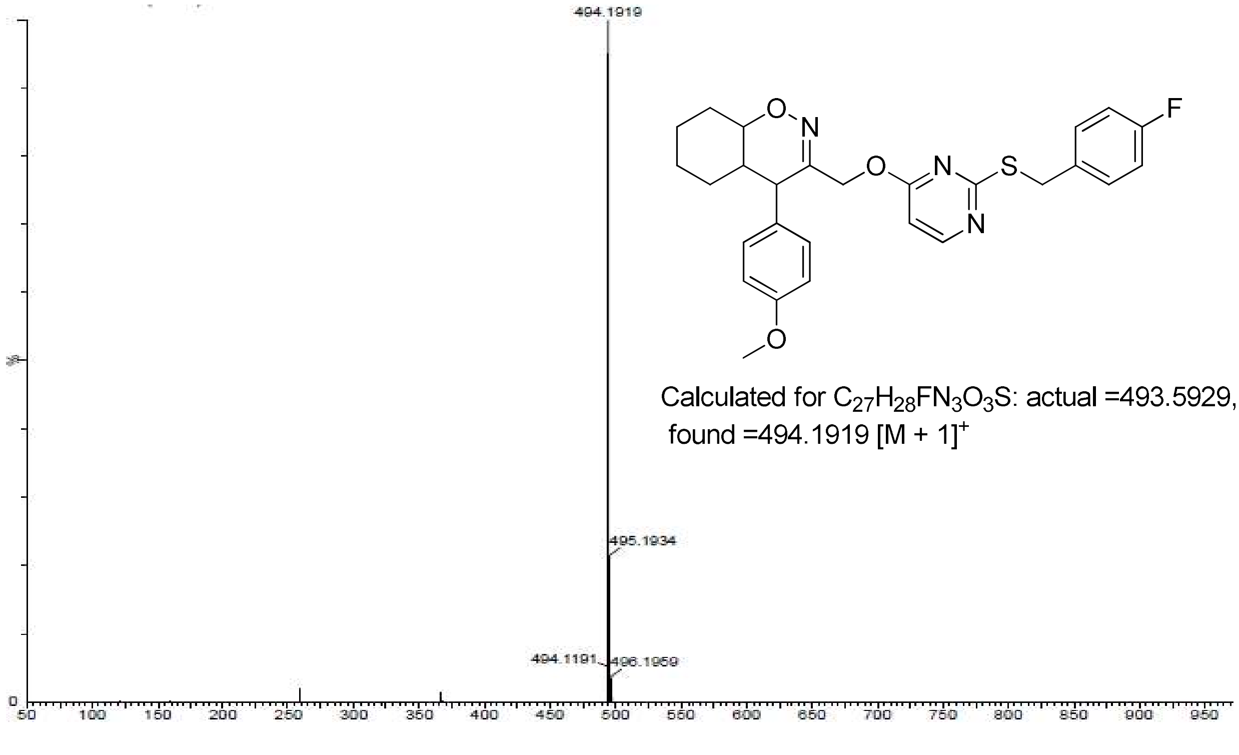

Mass spectra of **3h**

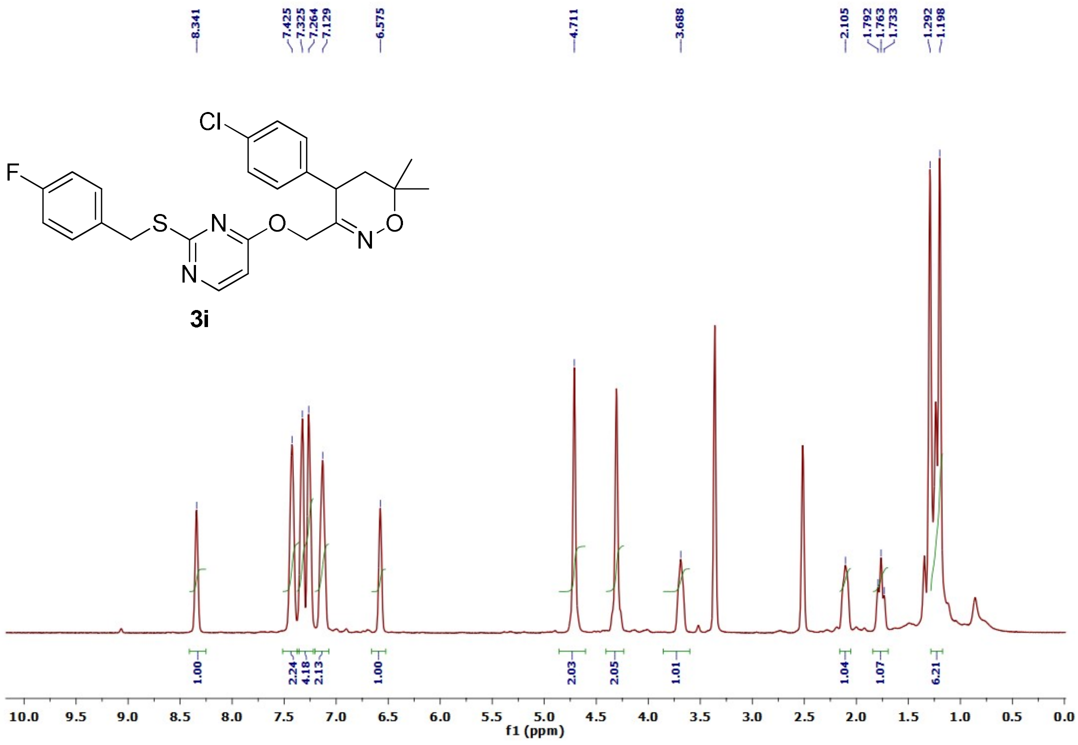

^{1}H NMR OF **3i**

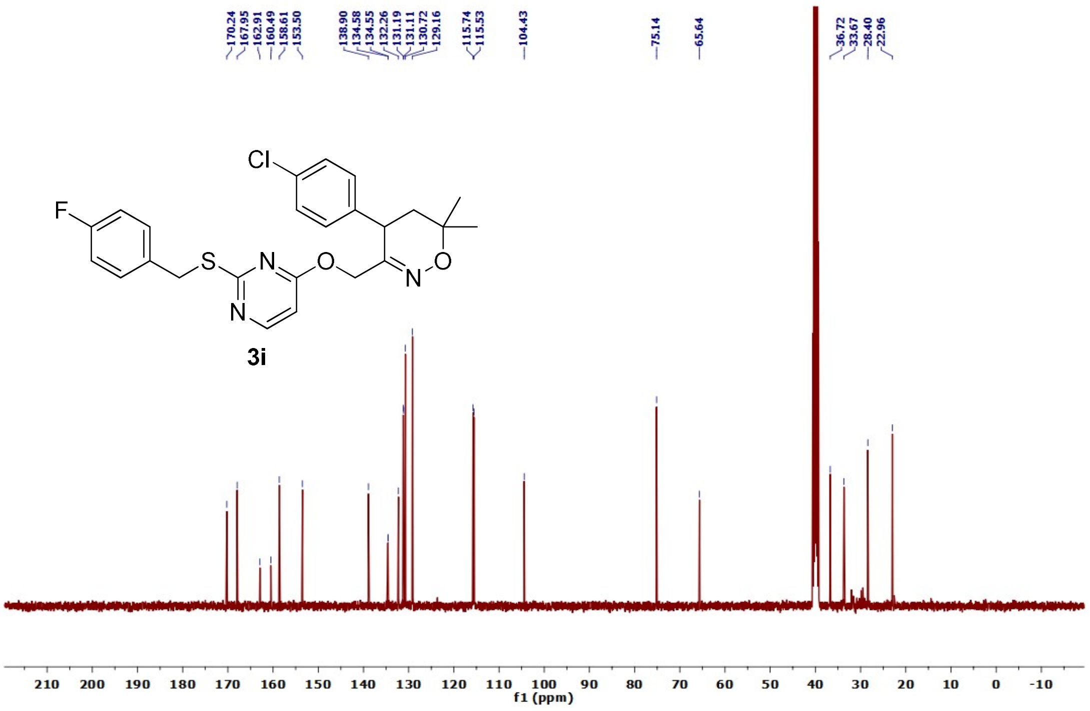

^{13}C NMR OF **3i**

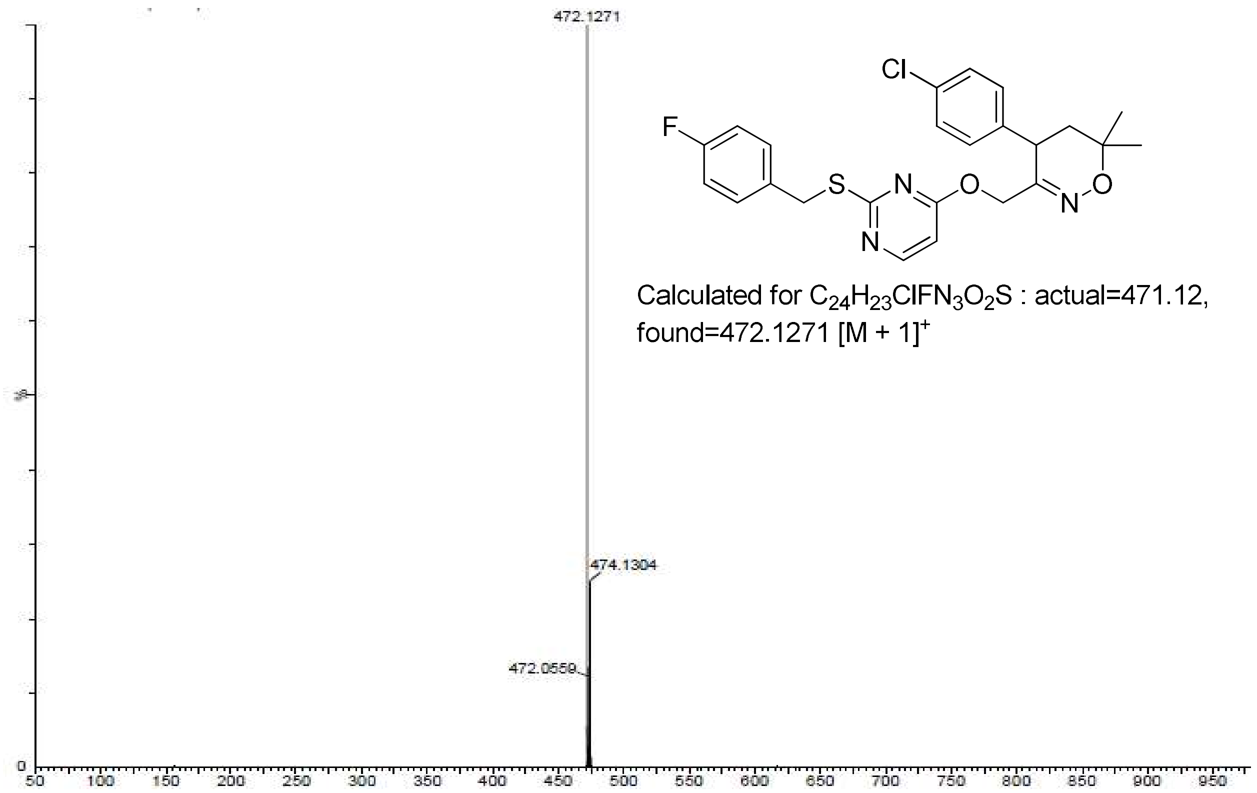

Mass spectra of **3i**

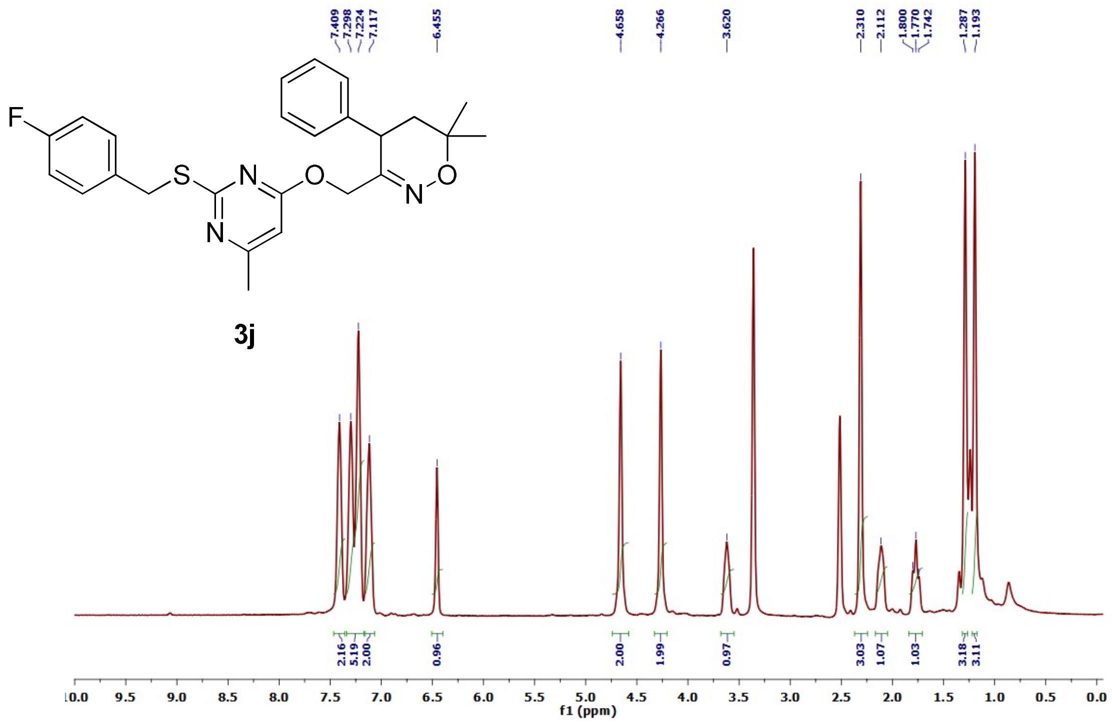

^{1}H NMR OF **3j**

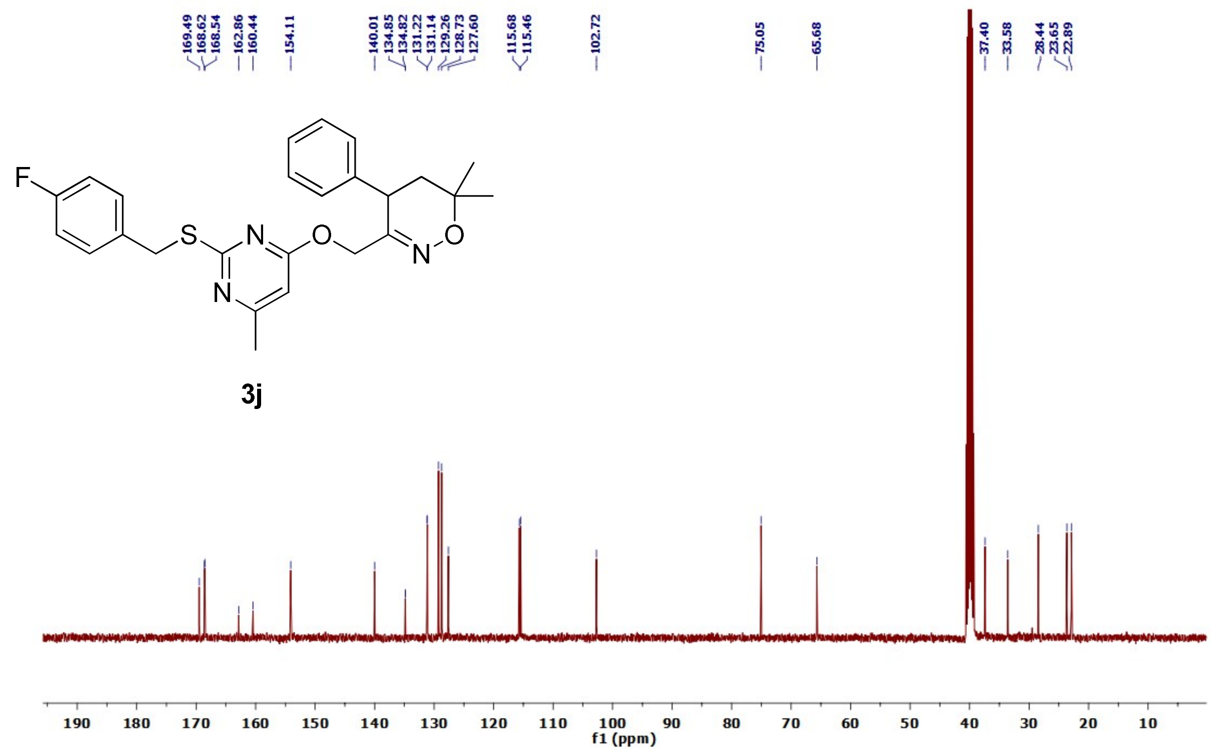

^{13}C NMR OF **3j**

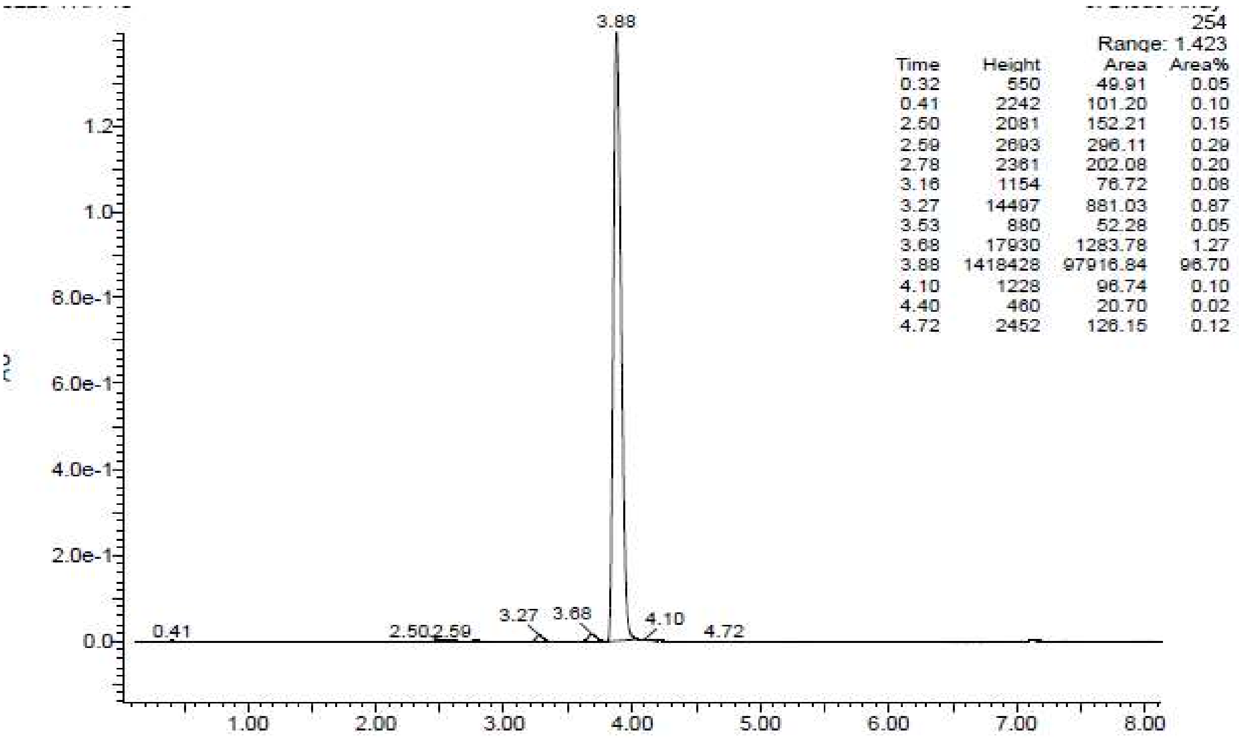

Liquid chromatogram of **3j**

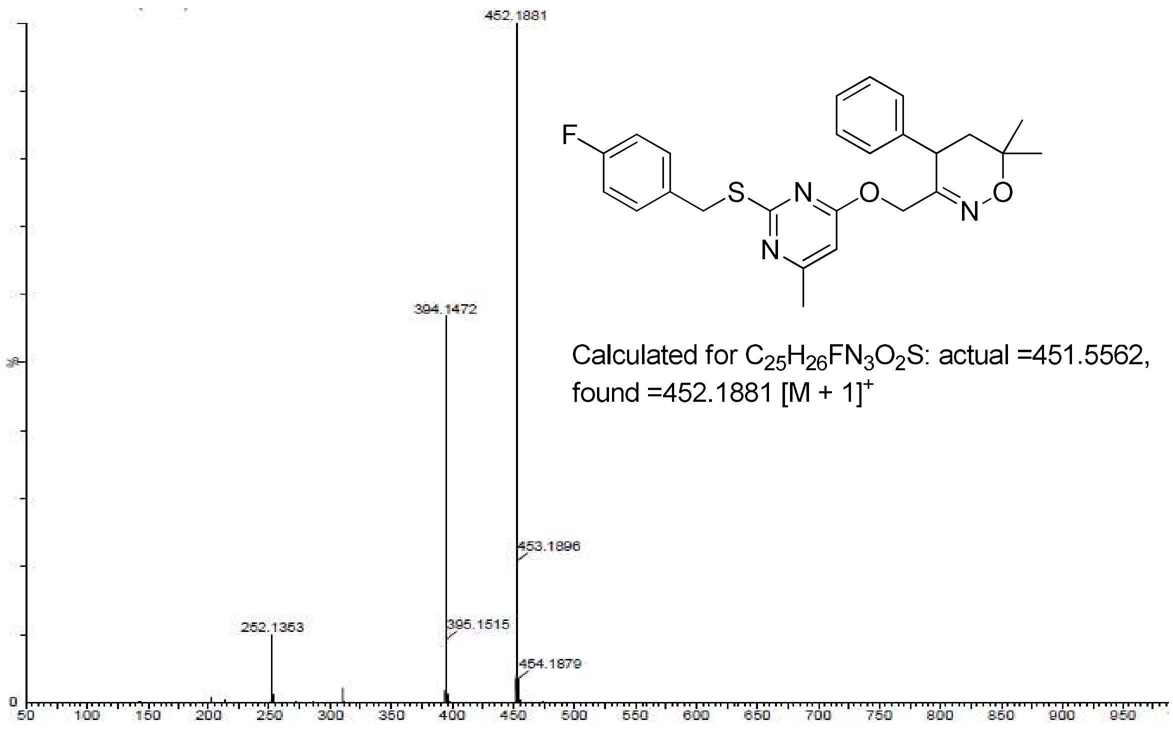

Mass spectra of **3j**

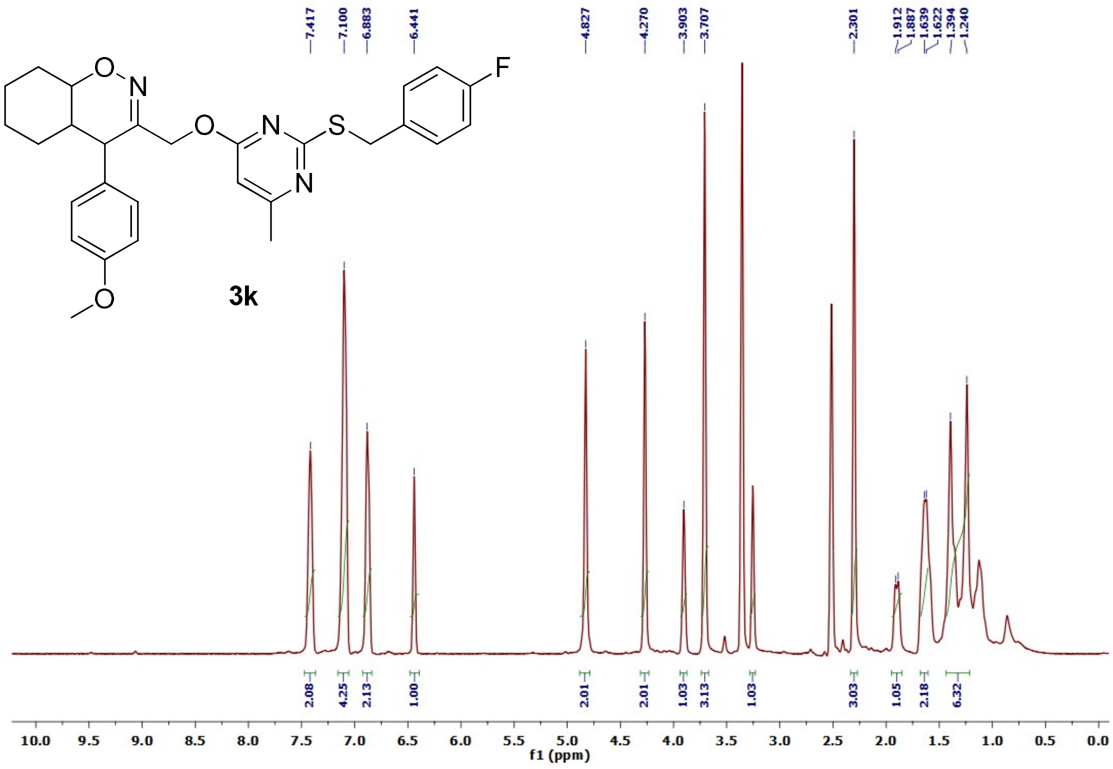

^{1}H NMR OF **3k**

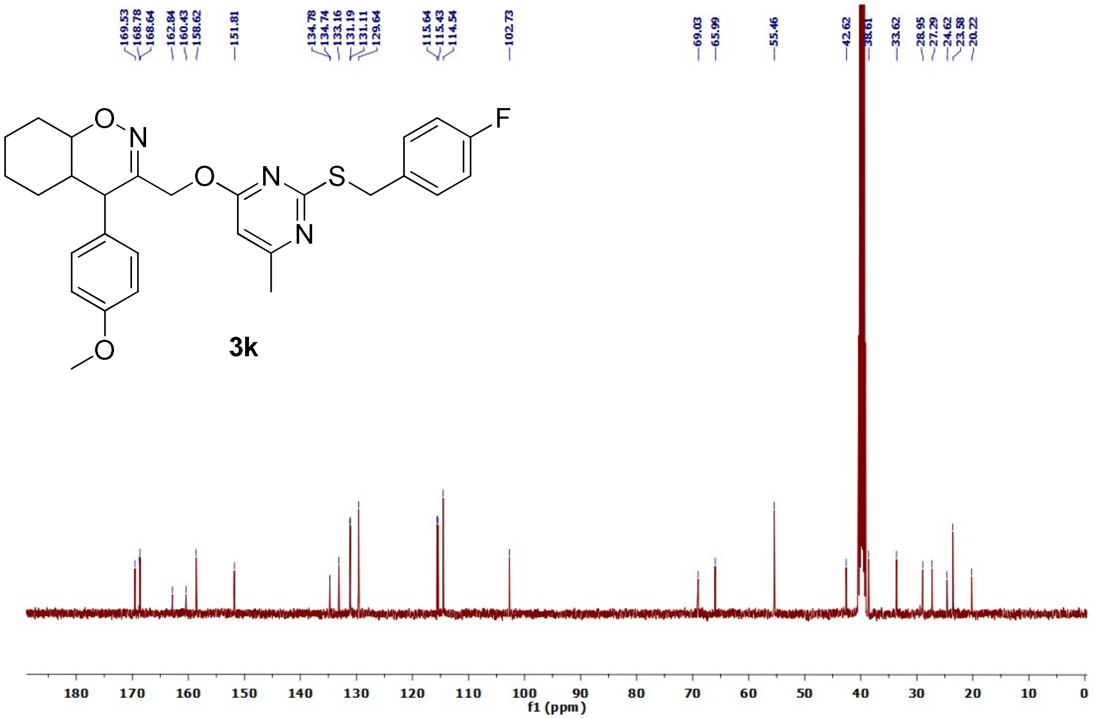

^{13}C NMR OF **3k**

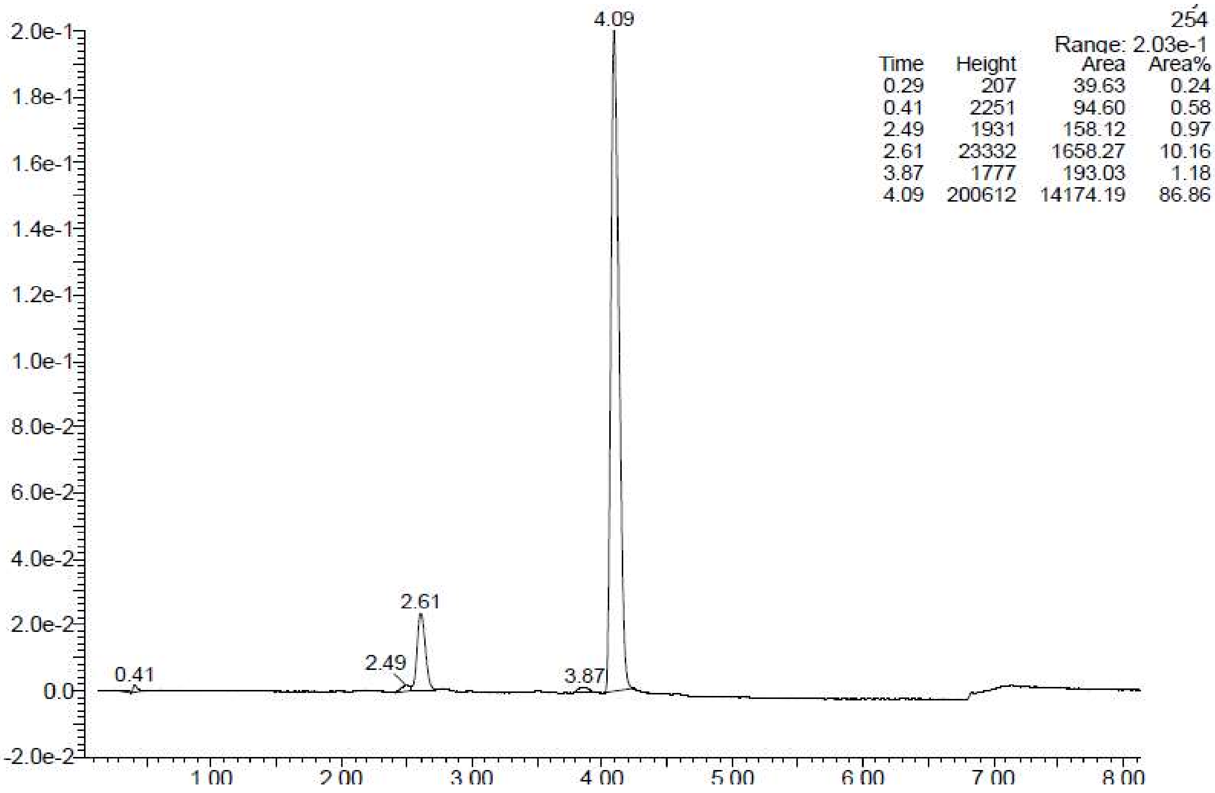

Liquid chromatogram of **3k**

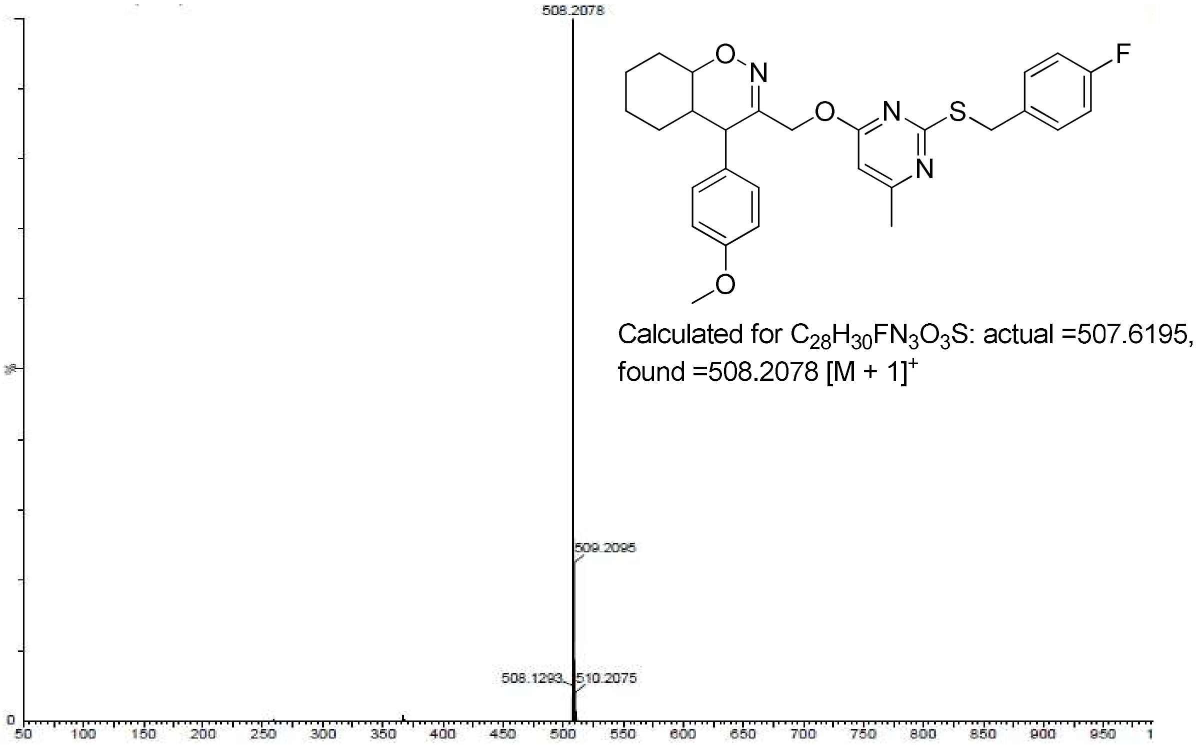

Mass spectra of **3k**

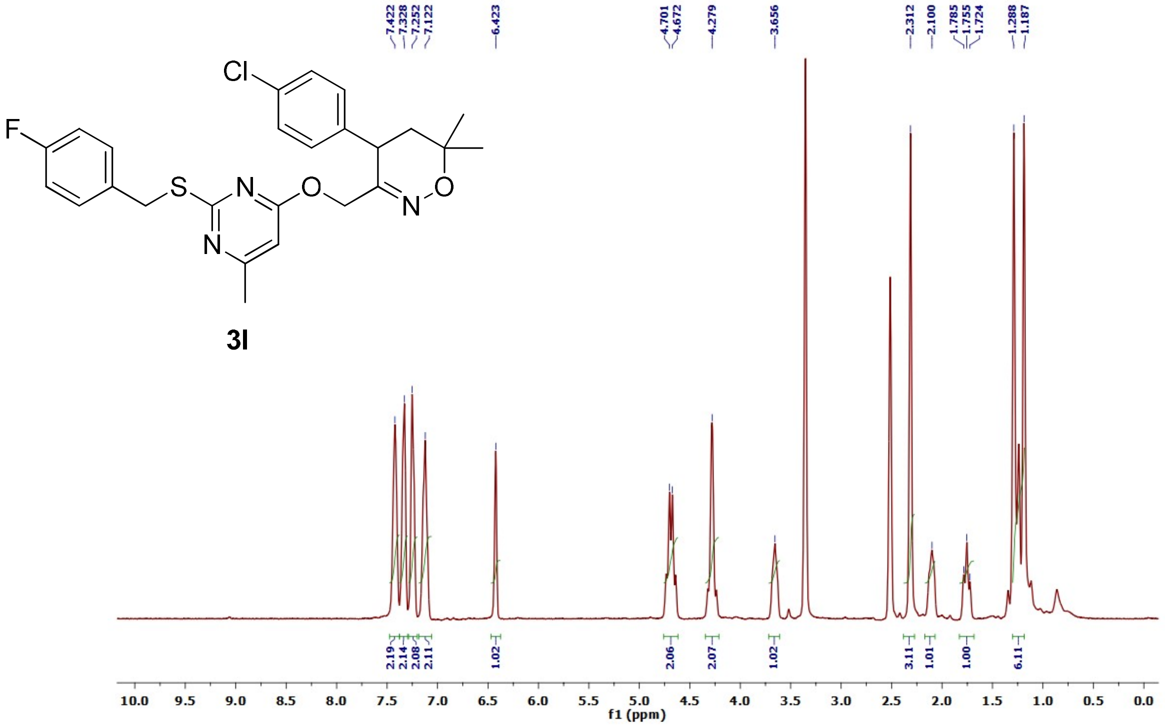

^{1}H NMR OF **3l**

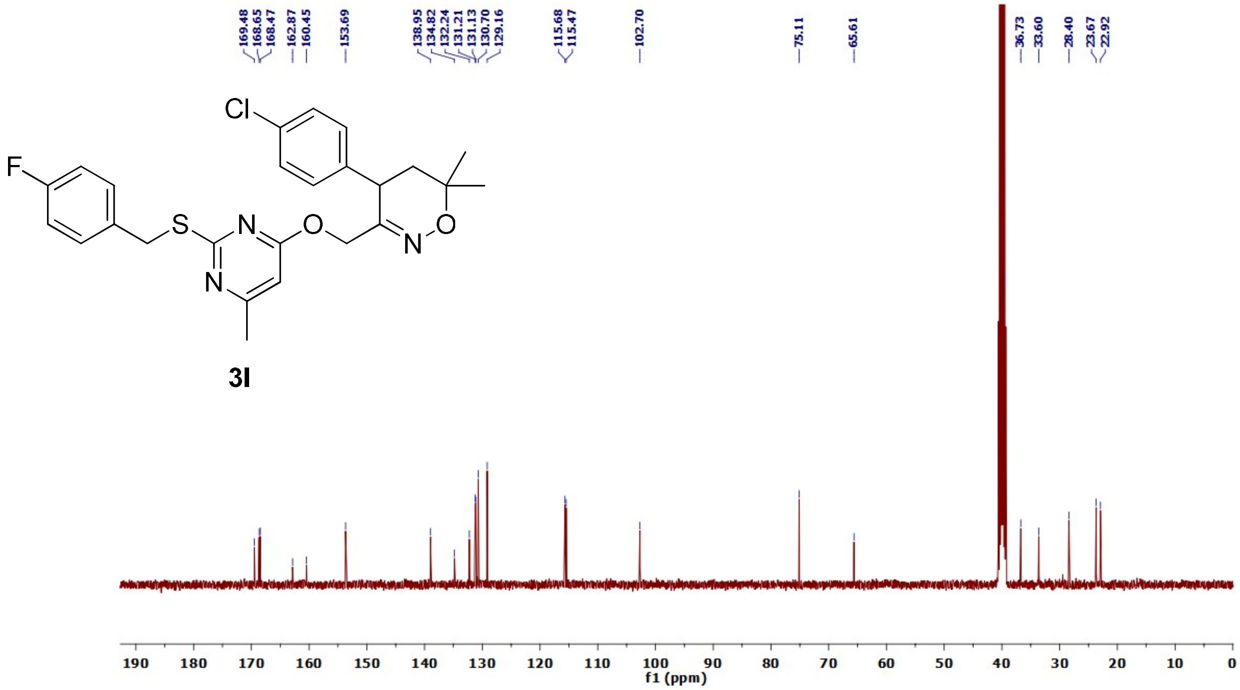

^{13}C NMR OF **3l**

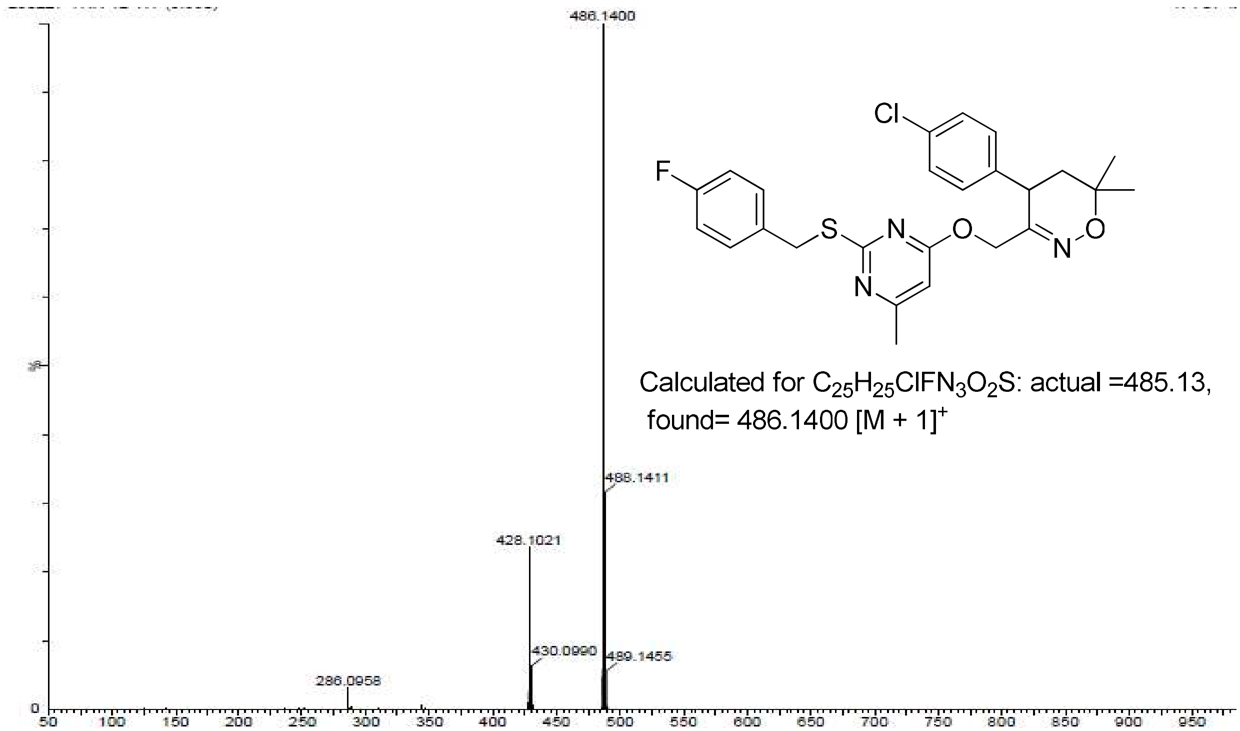

Mass spectra of **3l**

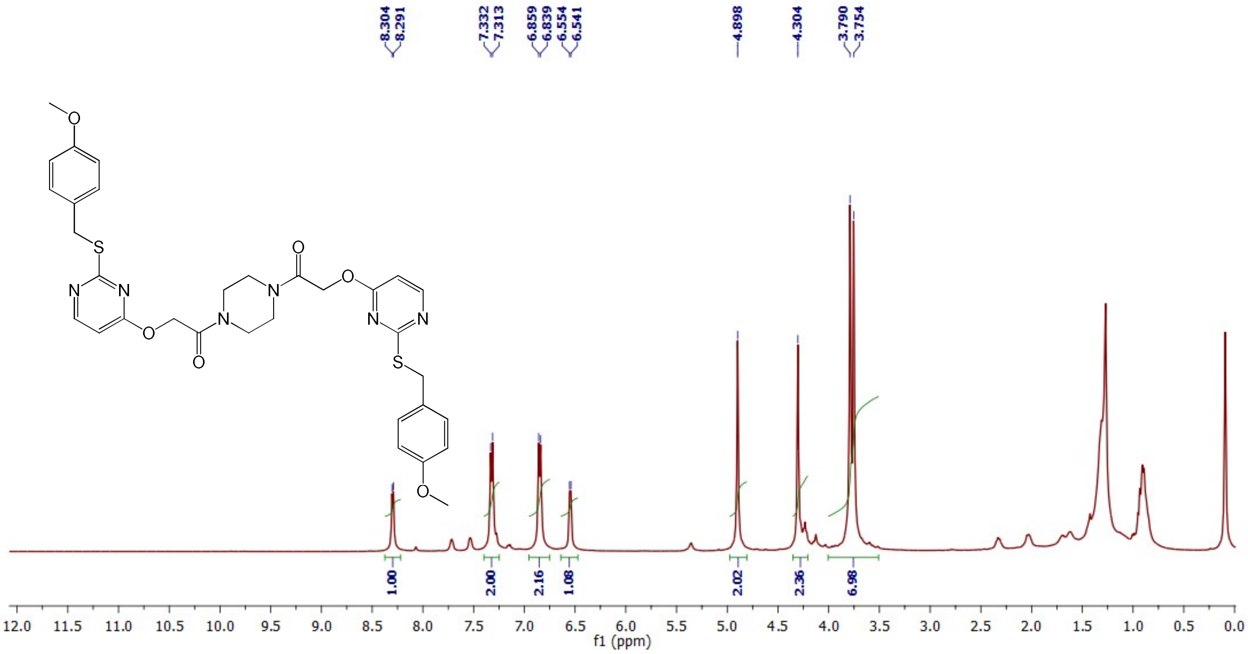

^{1}H NMR of **5a**

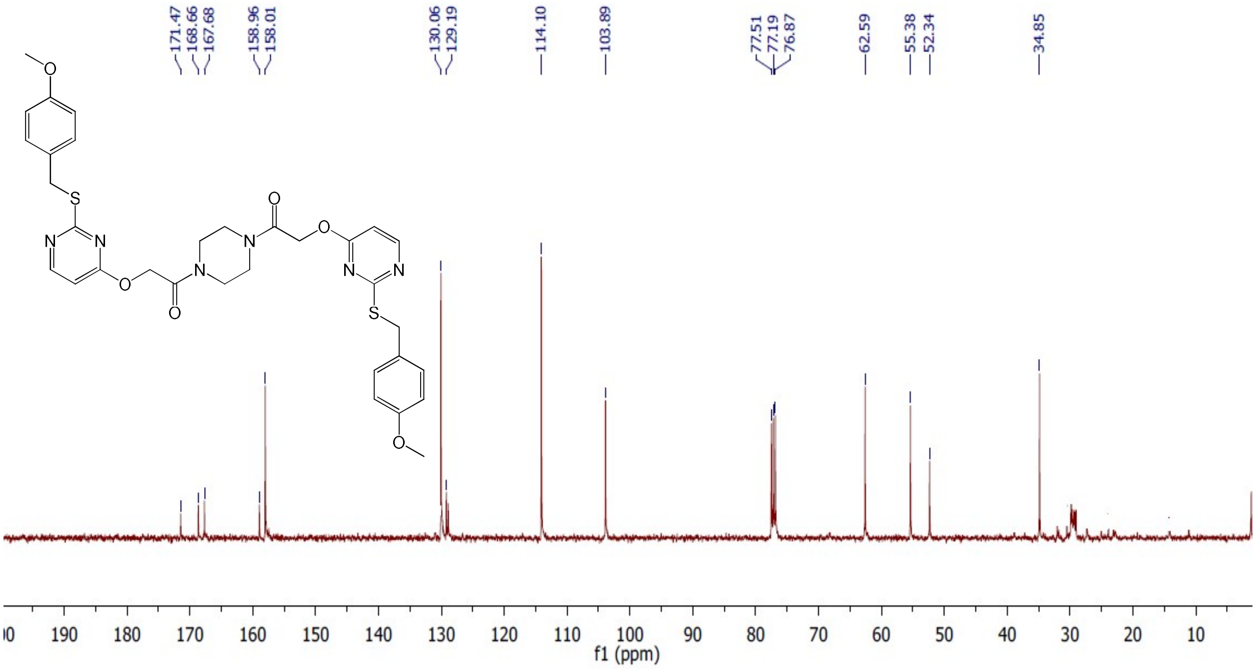

^{13}C NMR of **5a**

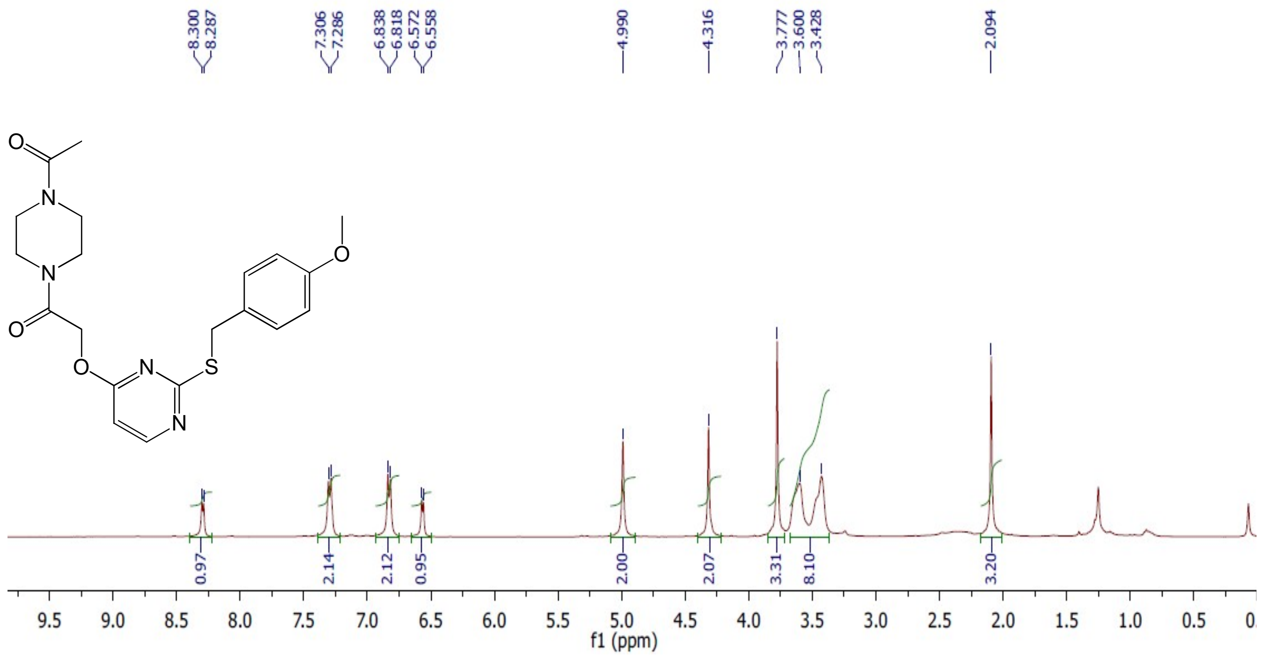

^{1}H NMR of 5**b**

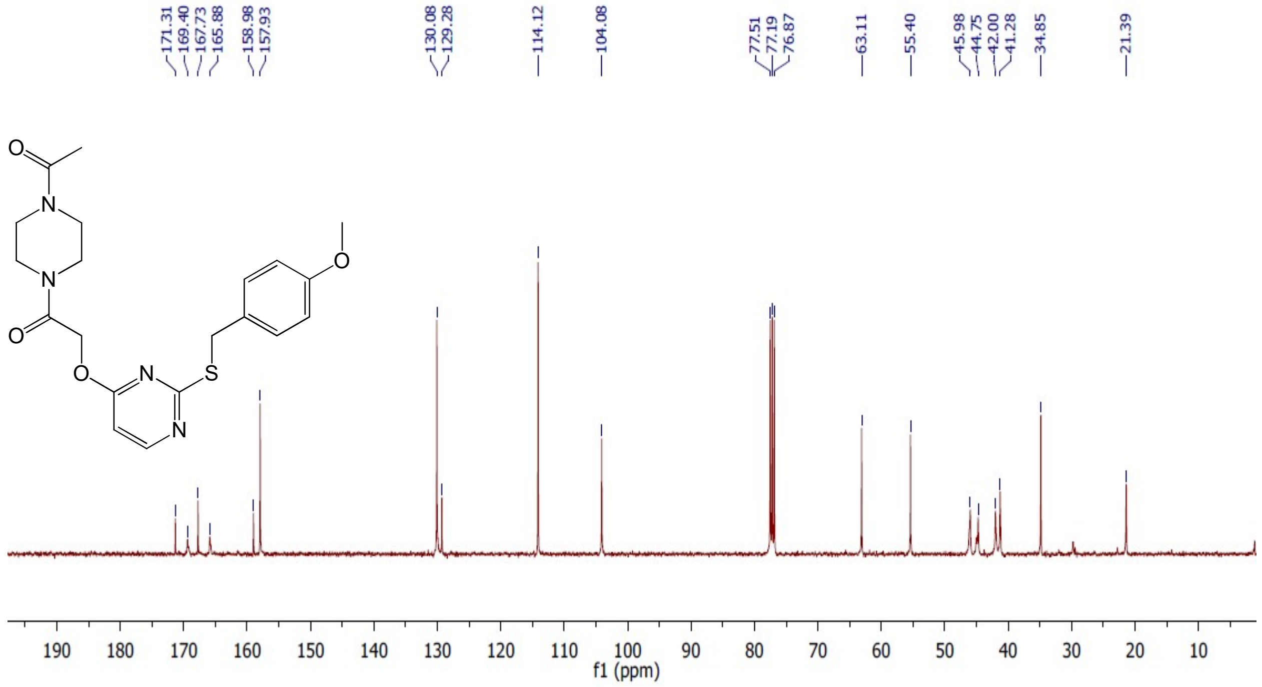

^{13}C NMR of **5b**

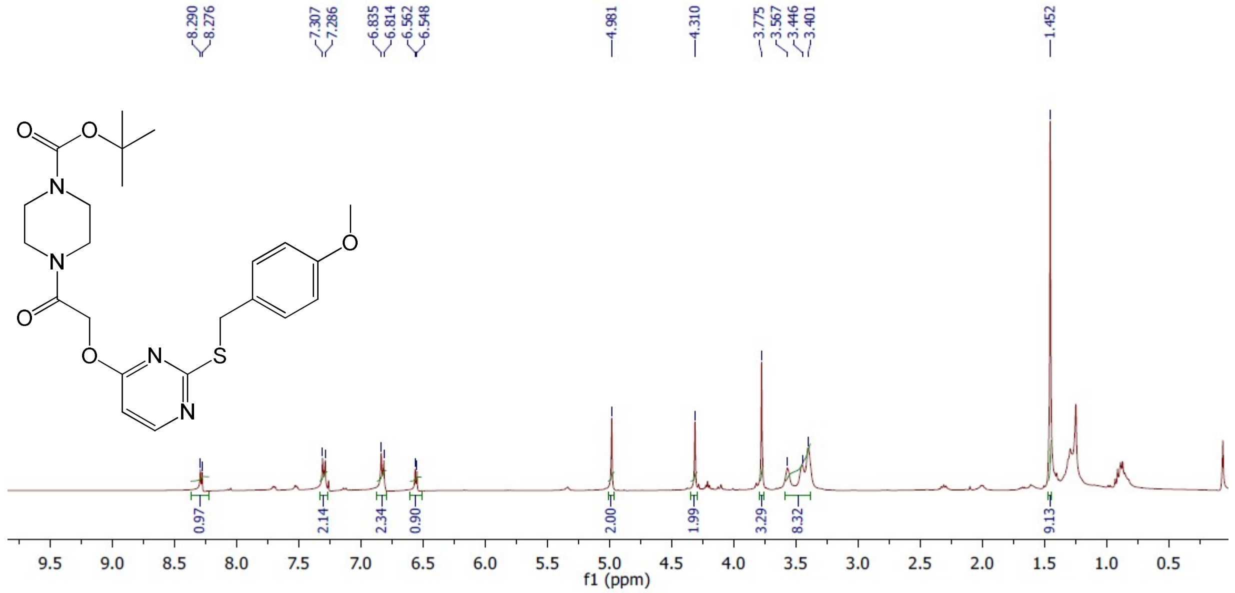

^{1}H NMR of **5c**

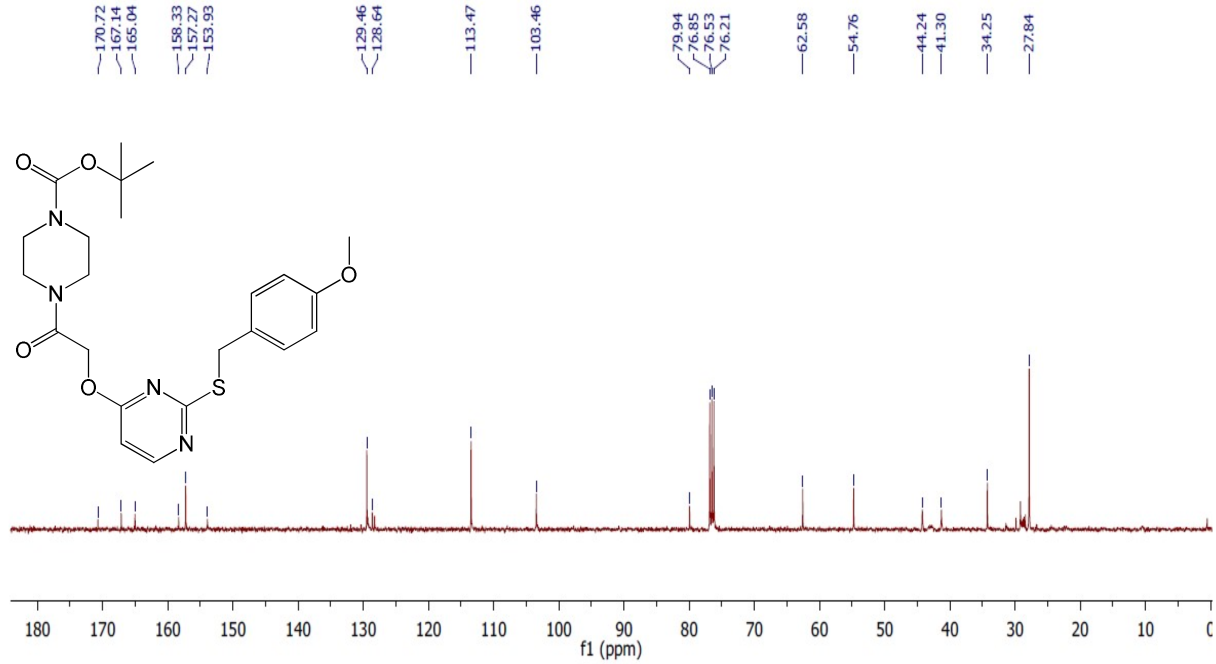

^{13}C NMR of **5c**

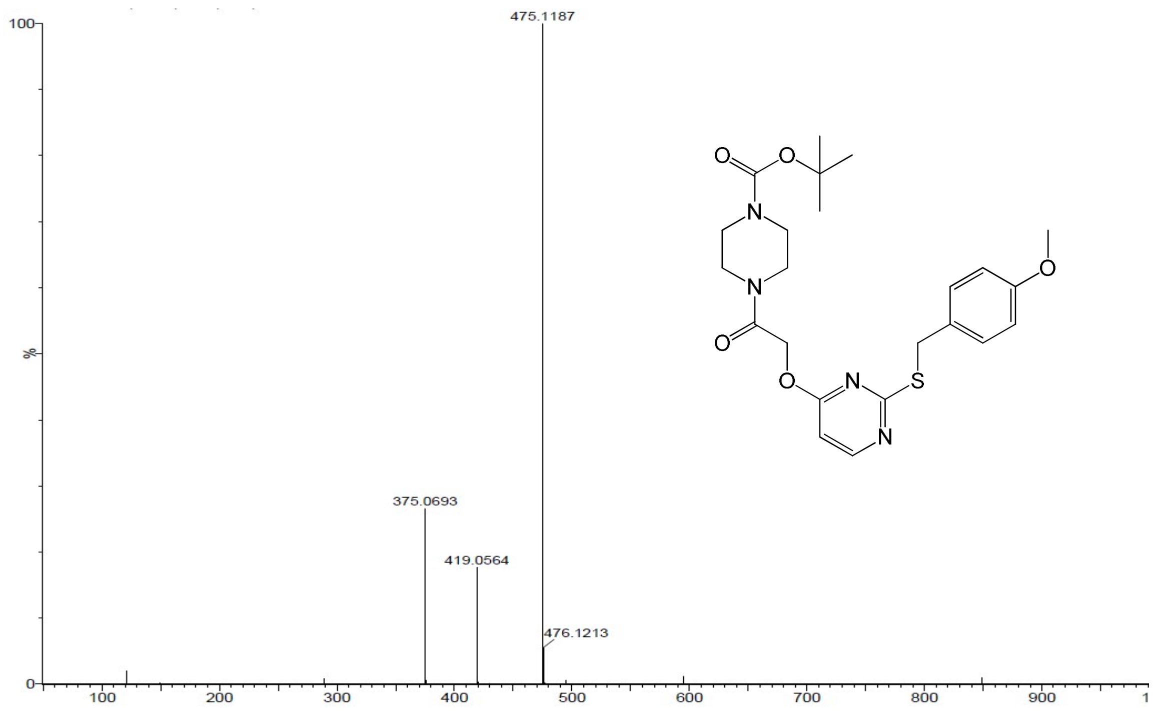

Mass spectra of **5c**

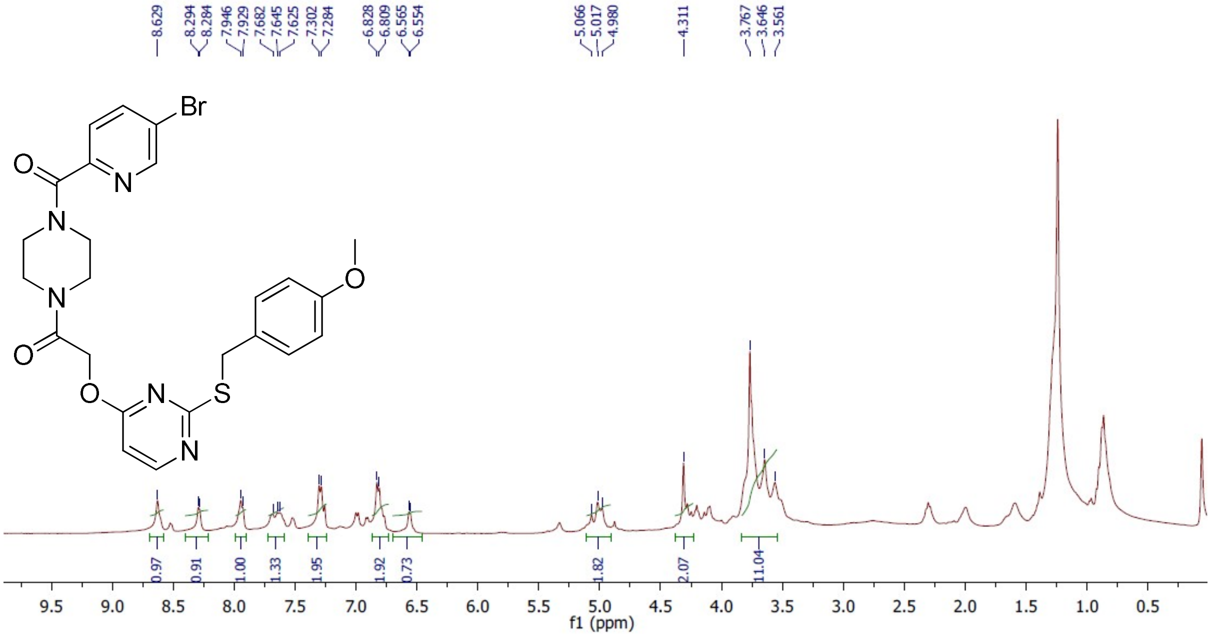

^{1}H NMR of 5**d**

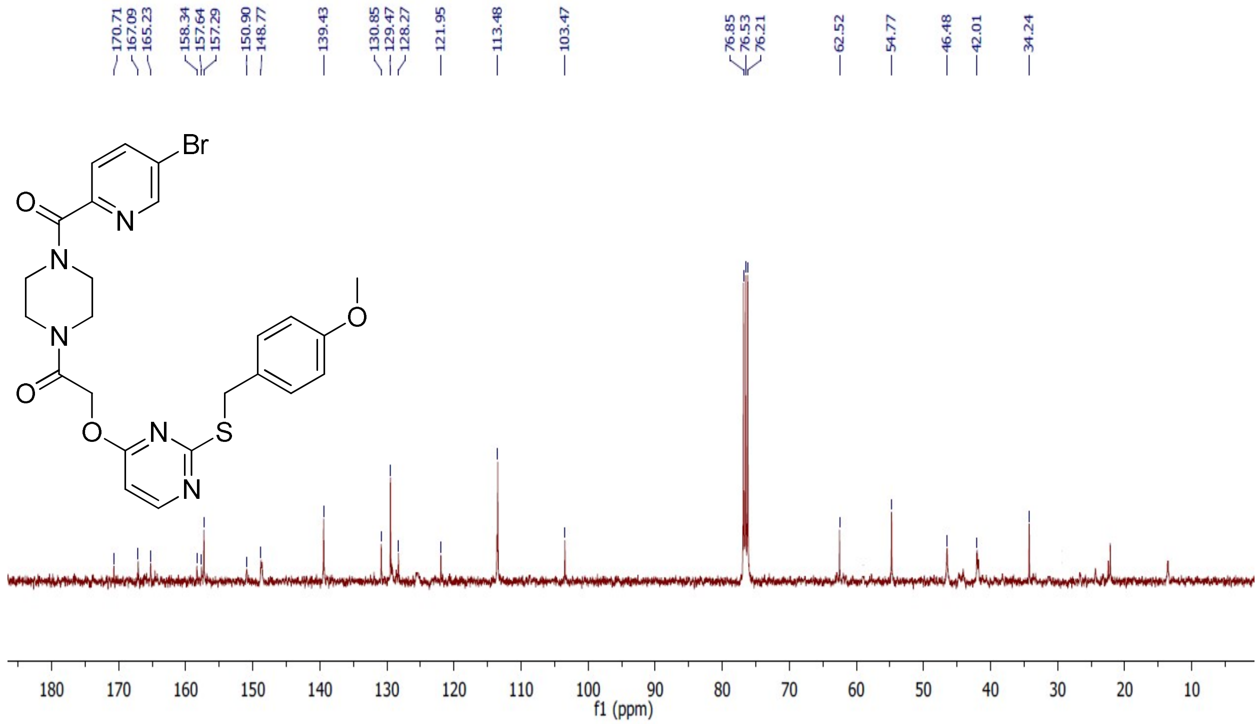

^{13}C NMR of **5d**

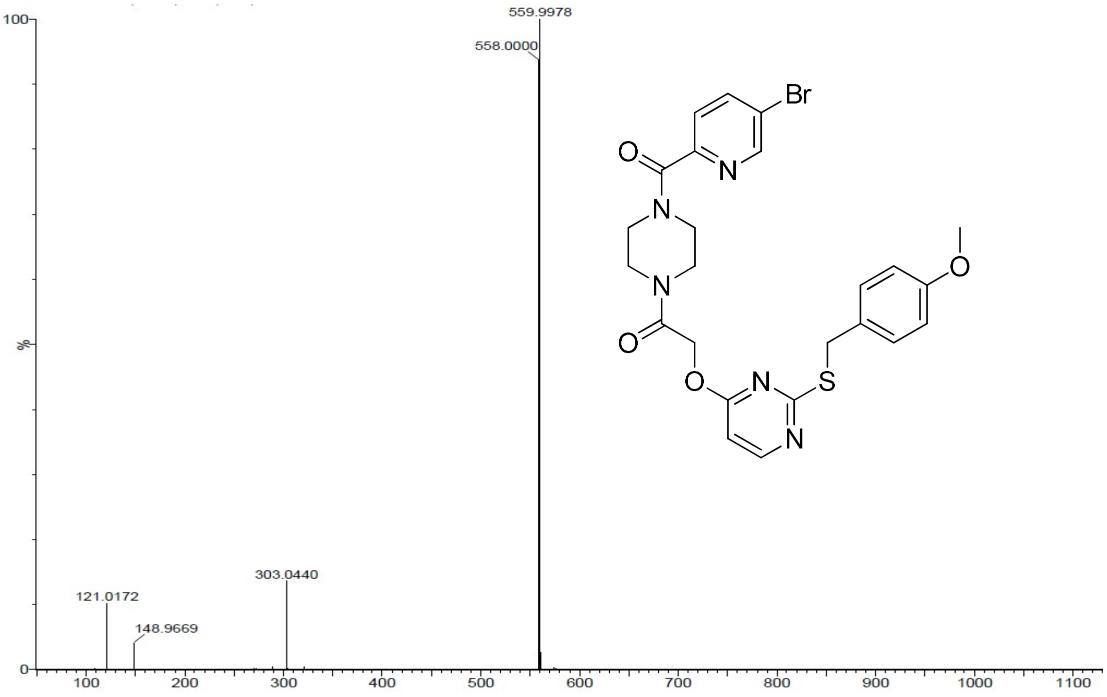

Mass spectra of **5d**

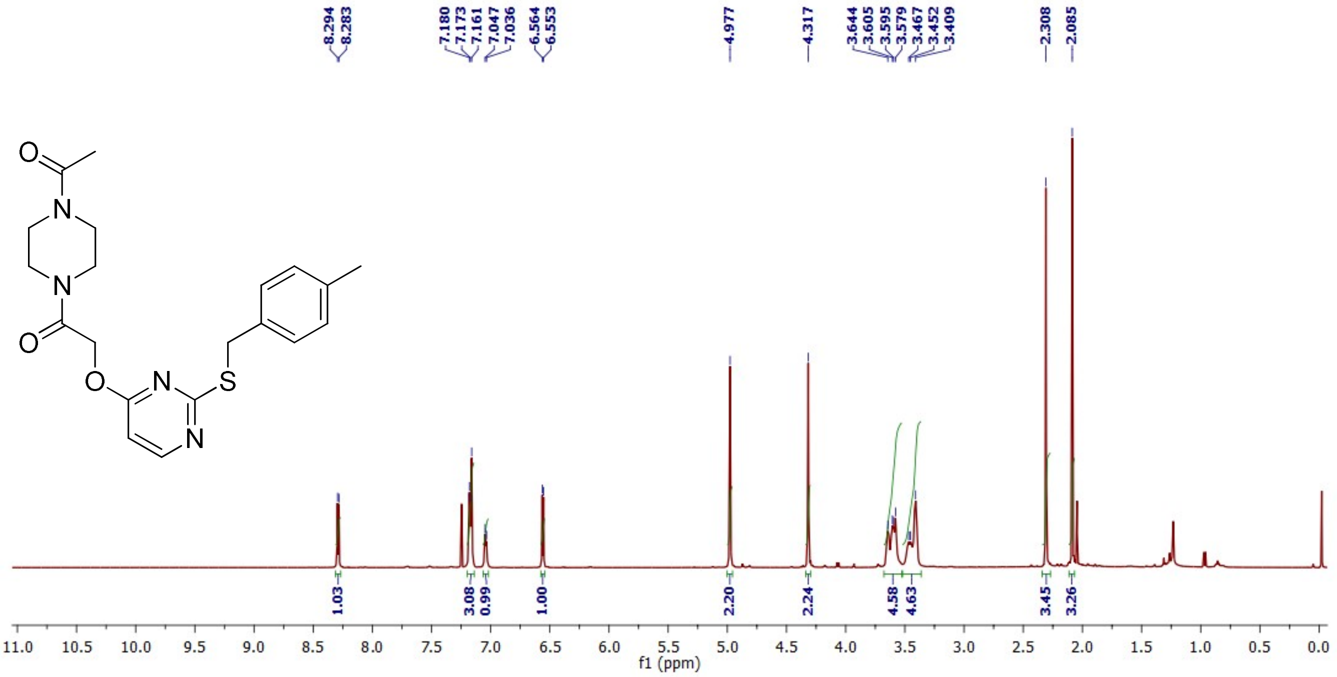

^{1}H NMR of 5**e**

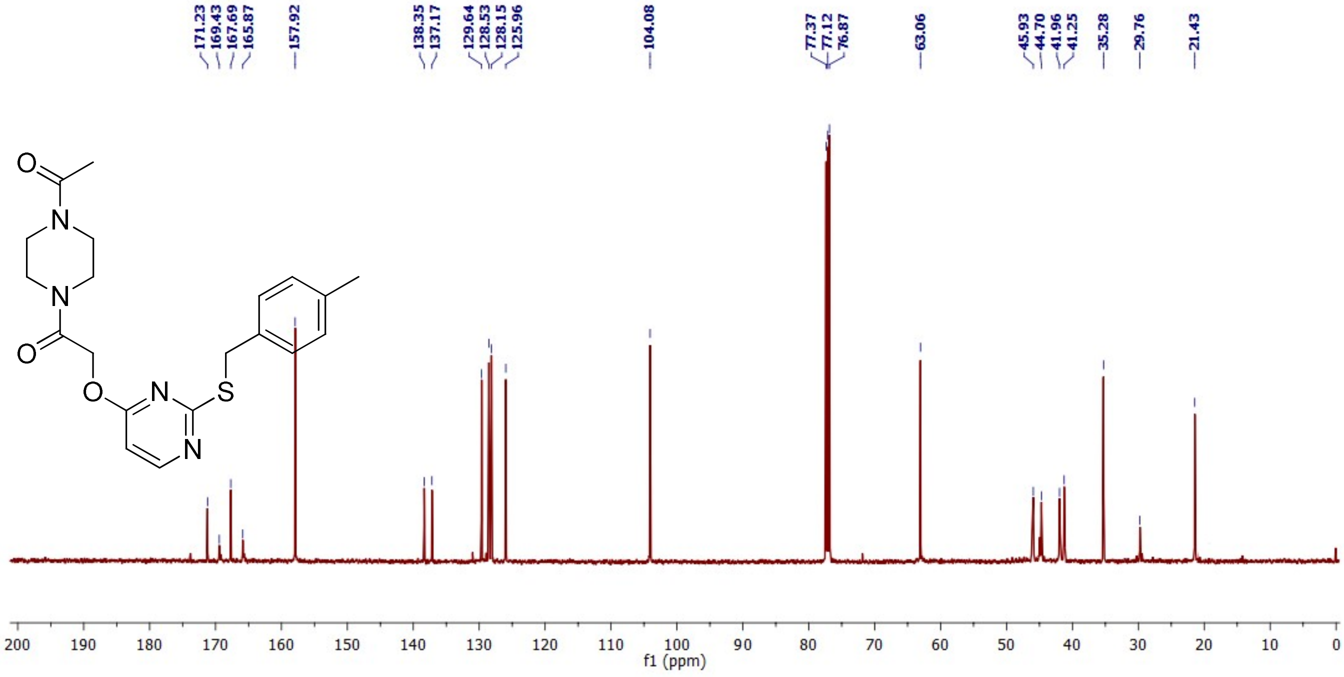

^{13}C NMR of 5**e**

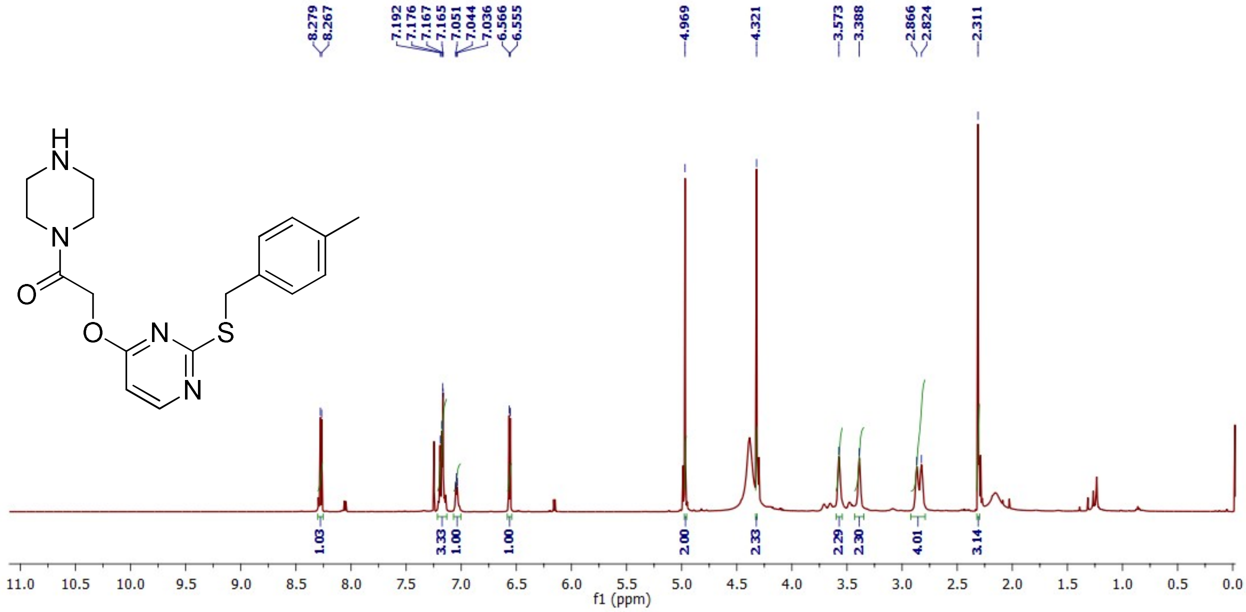

^{1}H NMR of 5**f**

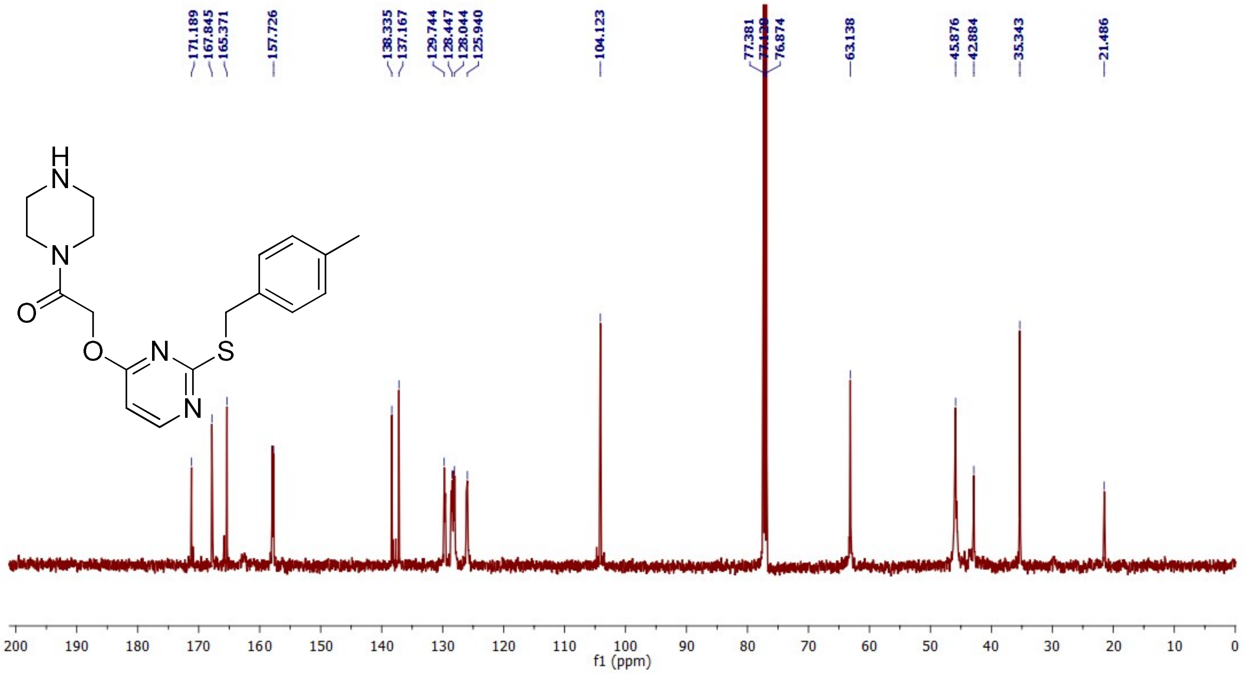

^{13}C NMR of 5**f**

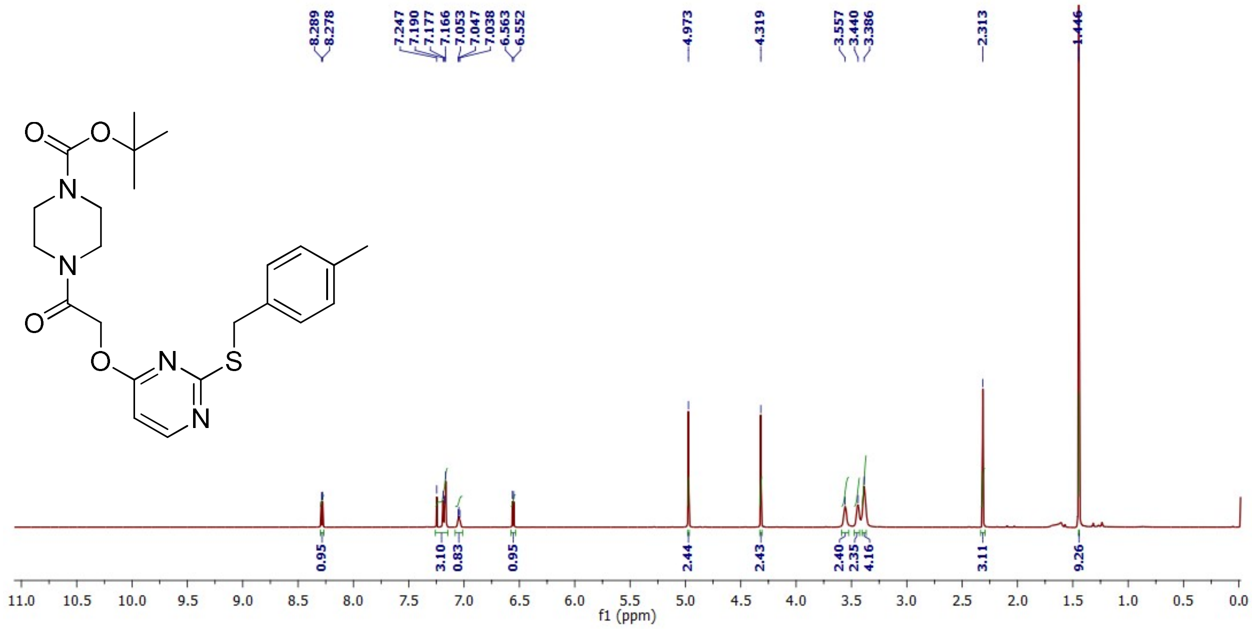

^{1}H NMR of 5**g**

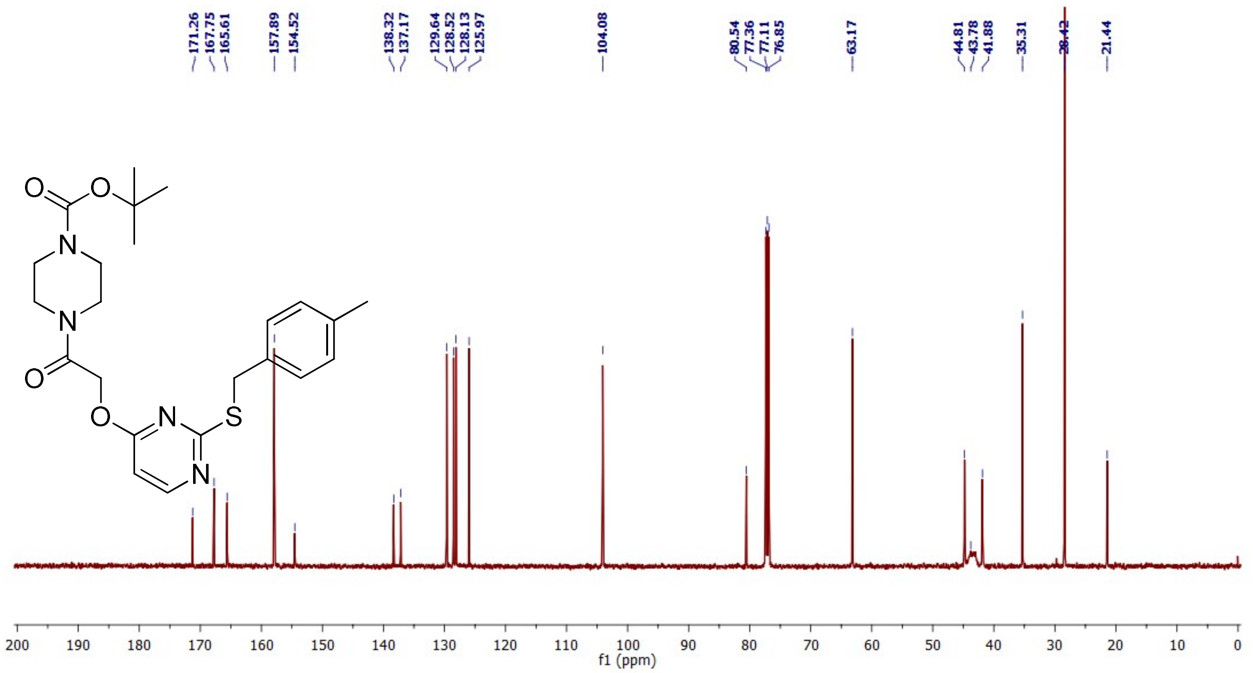

^{13}C NMR of 5**g**

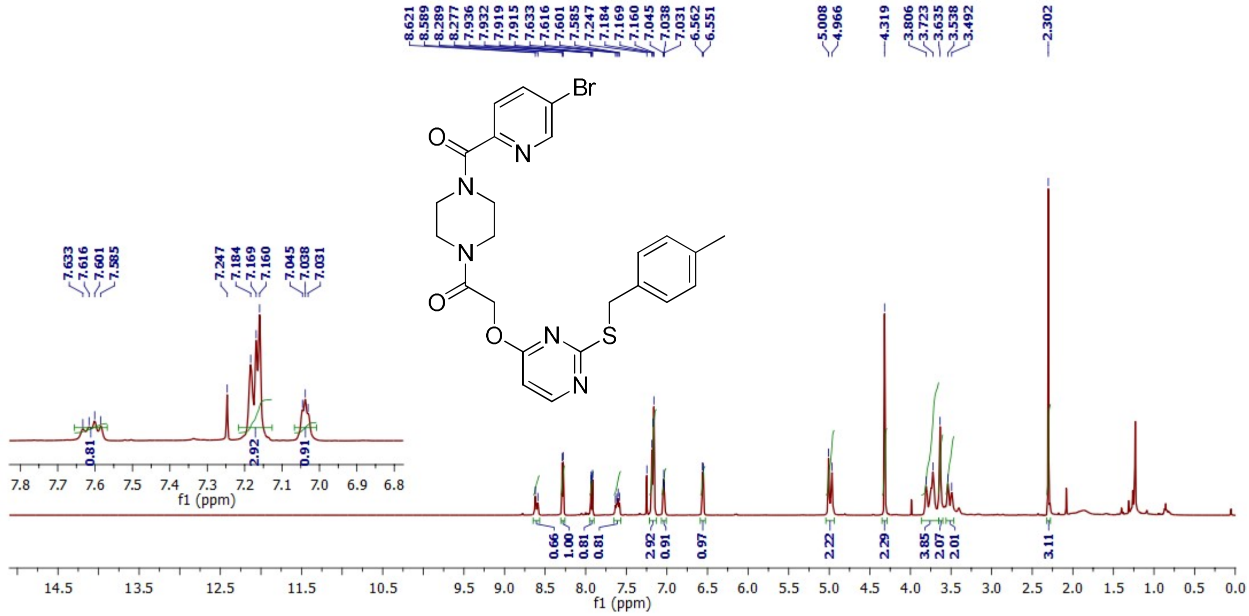

^{1}H NMR of 5**h**

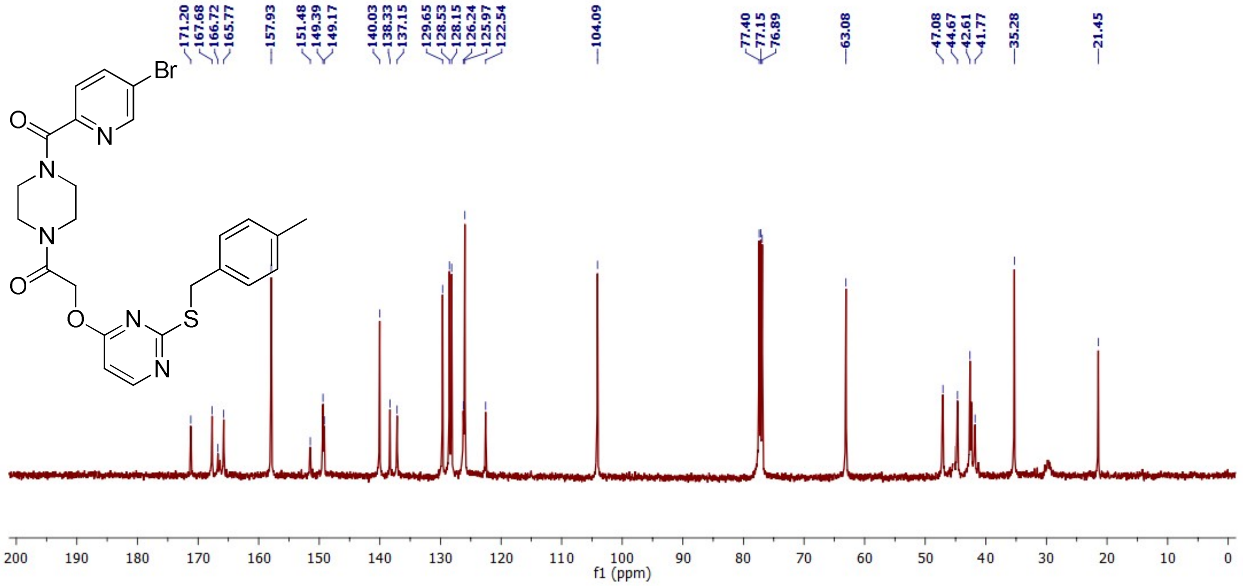

^{13}C NMR of 5**h**

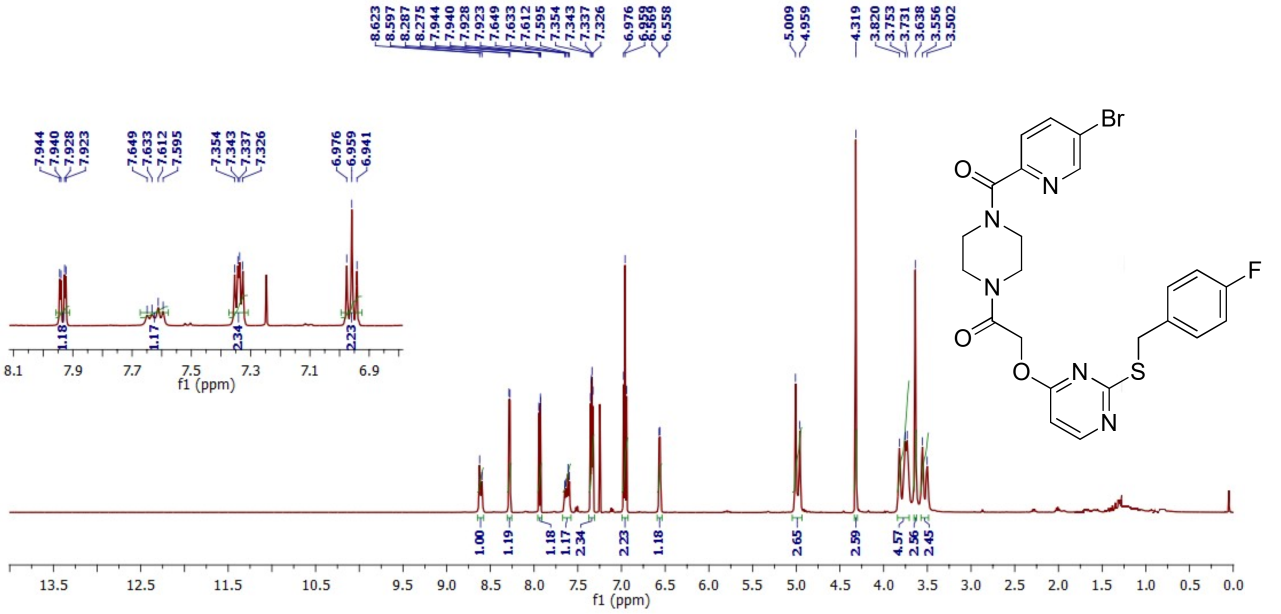

^{1}H NMR of 5**i**

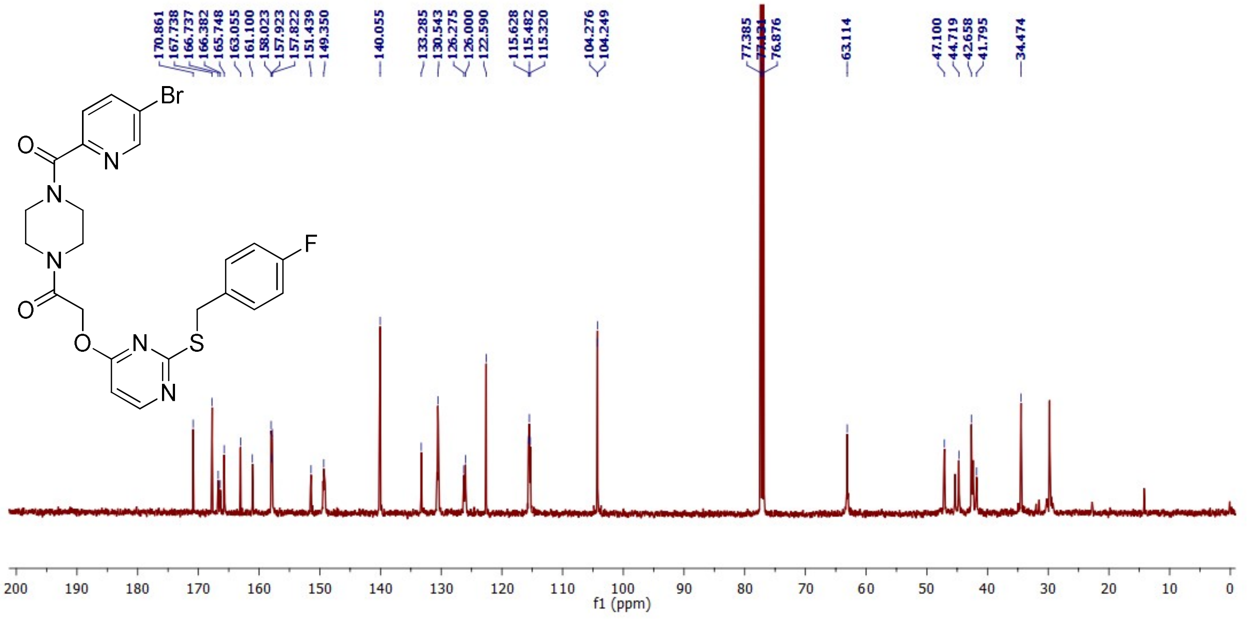

^{13}C NMR of 5**i**

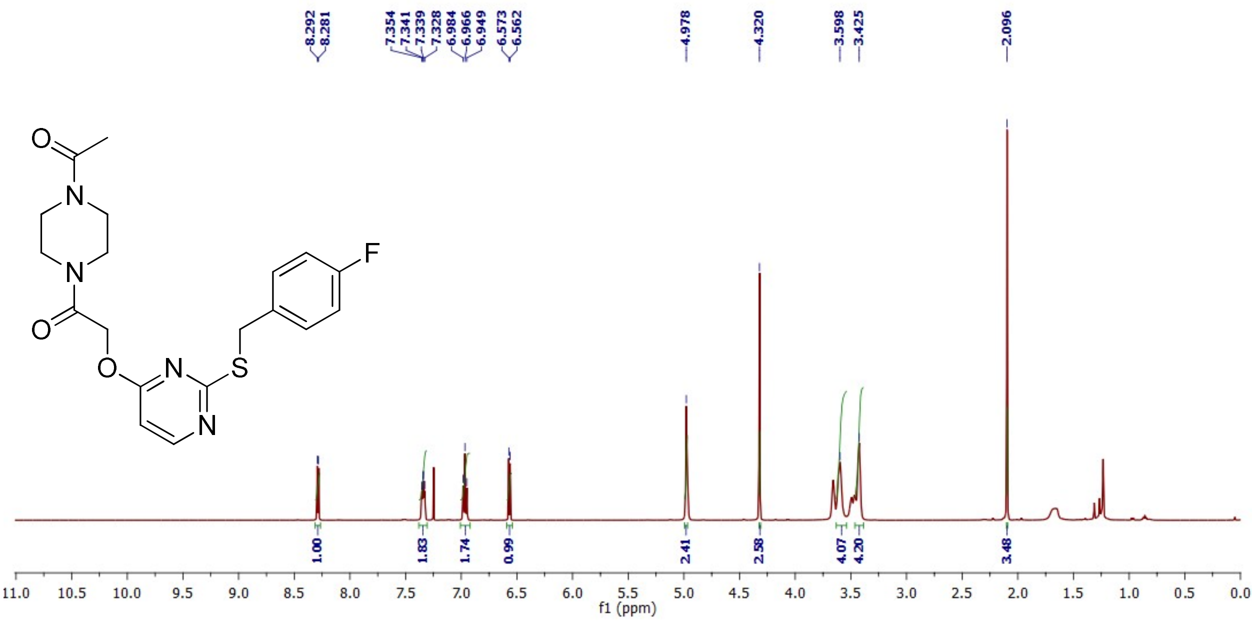

^{1}H NMR of 5**j**

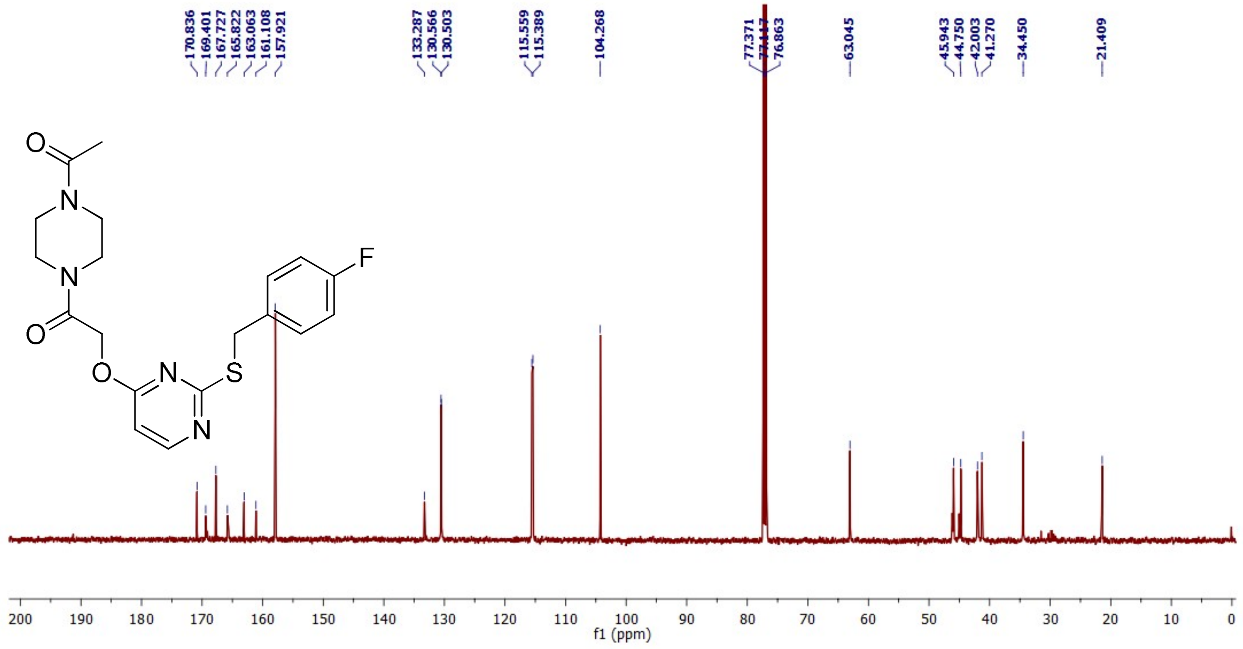

^{13}C NMR of 5**j**

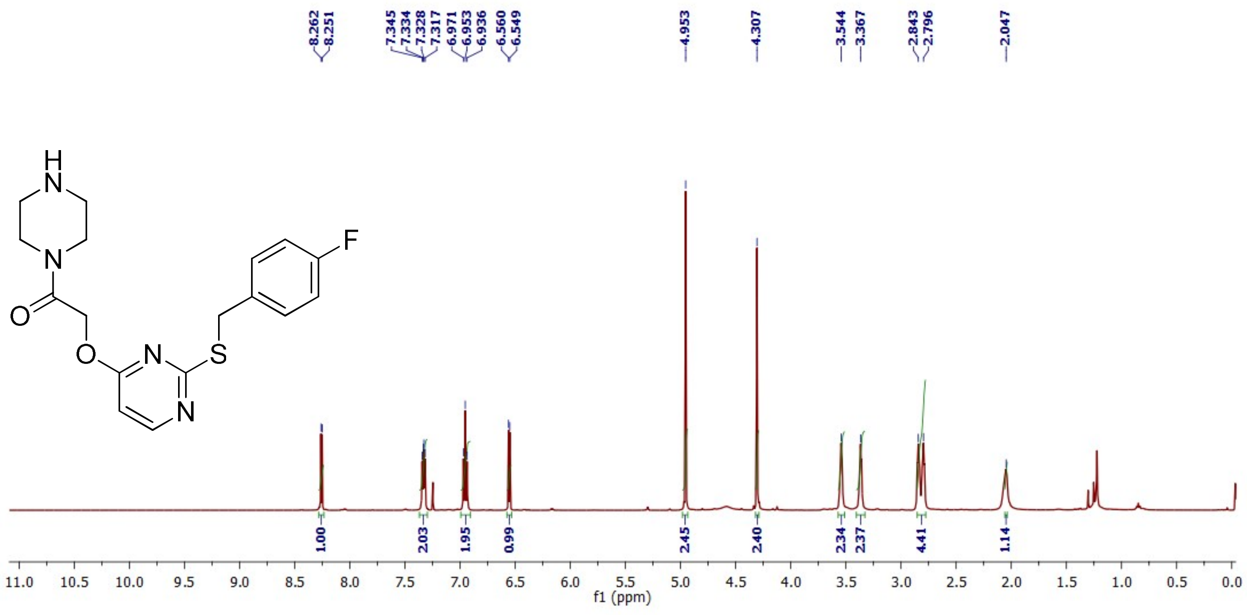

^{1}H NMR of 5**k**

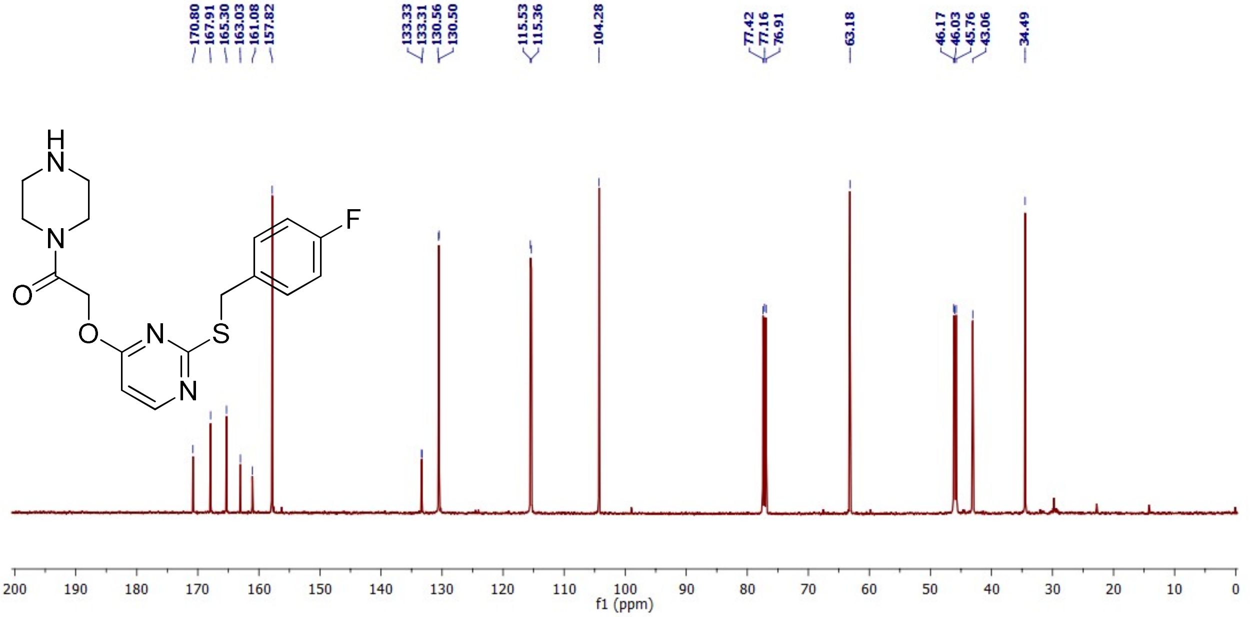

^{13}C NMR of 5**k**

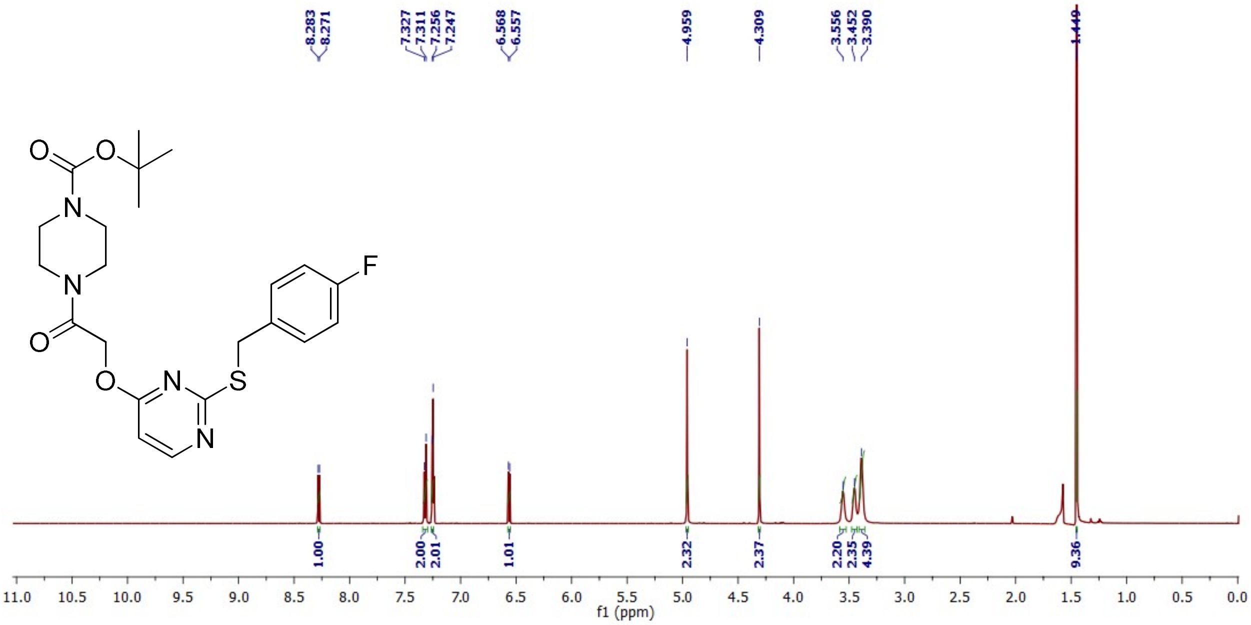

^{1}H NMR of 5**l**

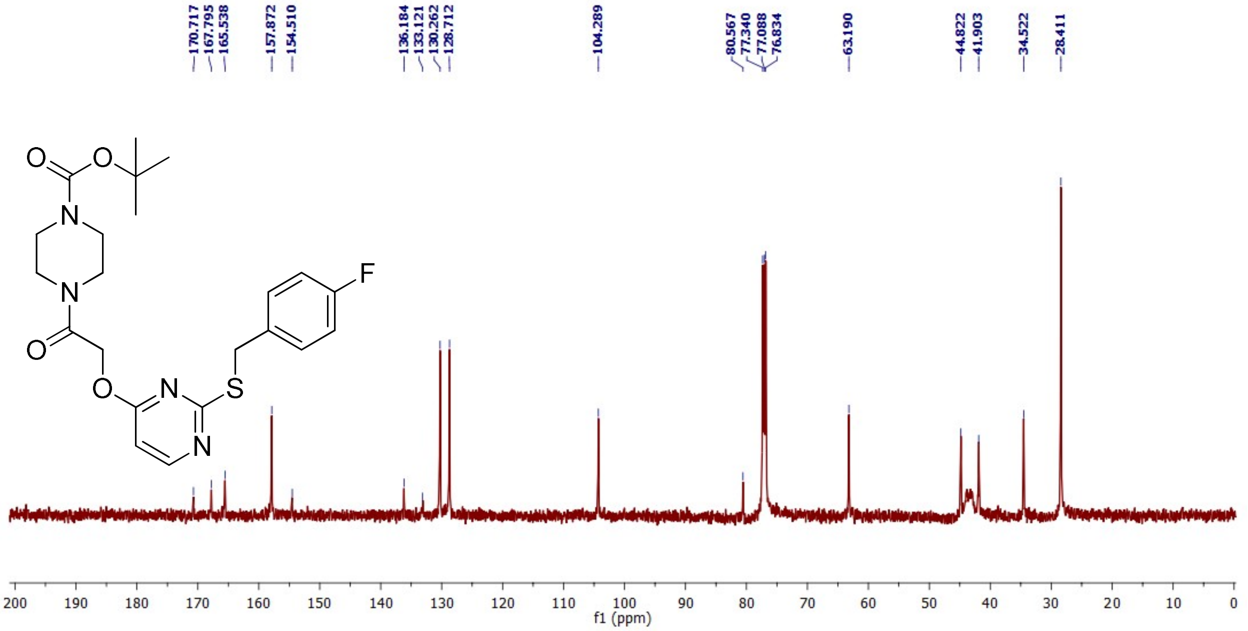

^{13}C NMR of 5**l**

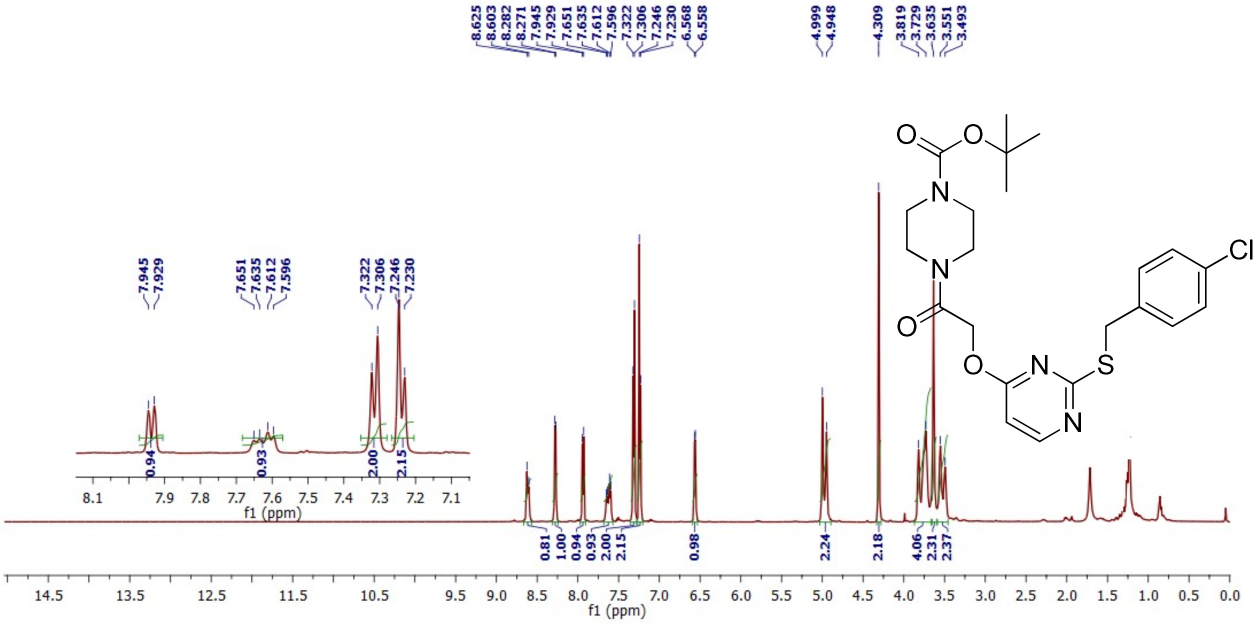

^{1}H NMR of 5**m**

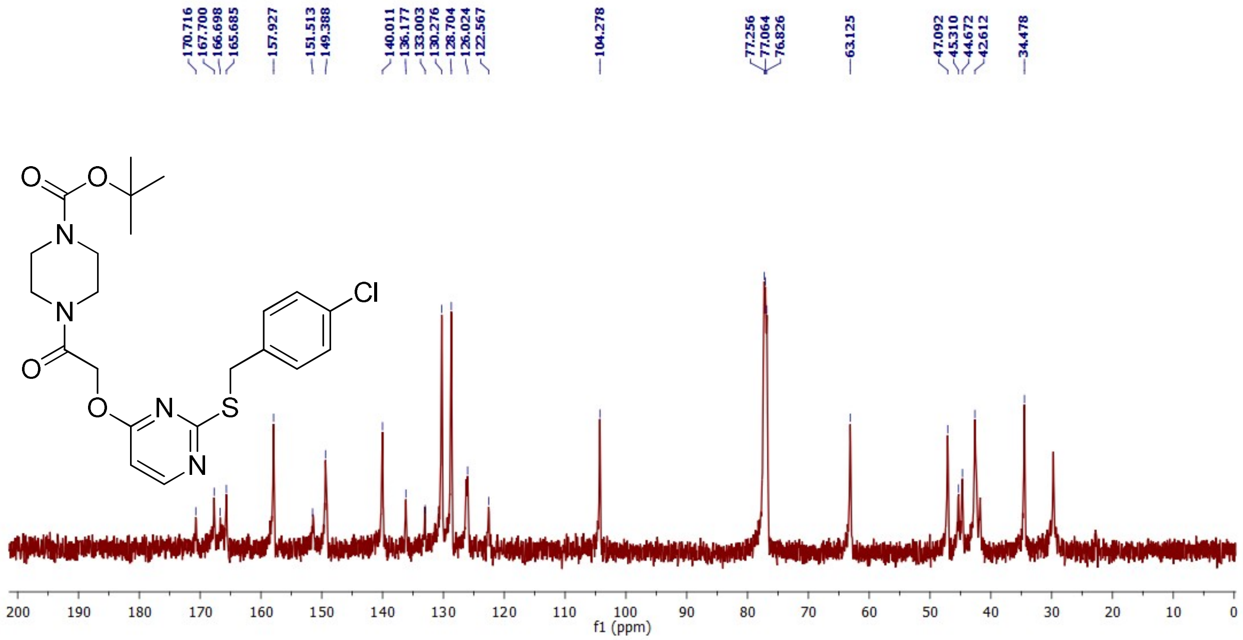

^{13}C NMR of 5**m**

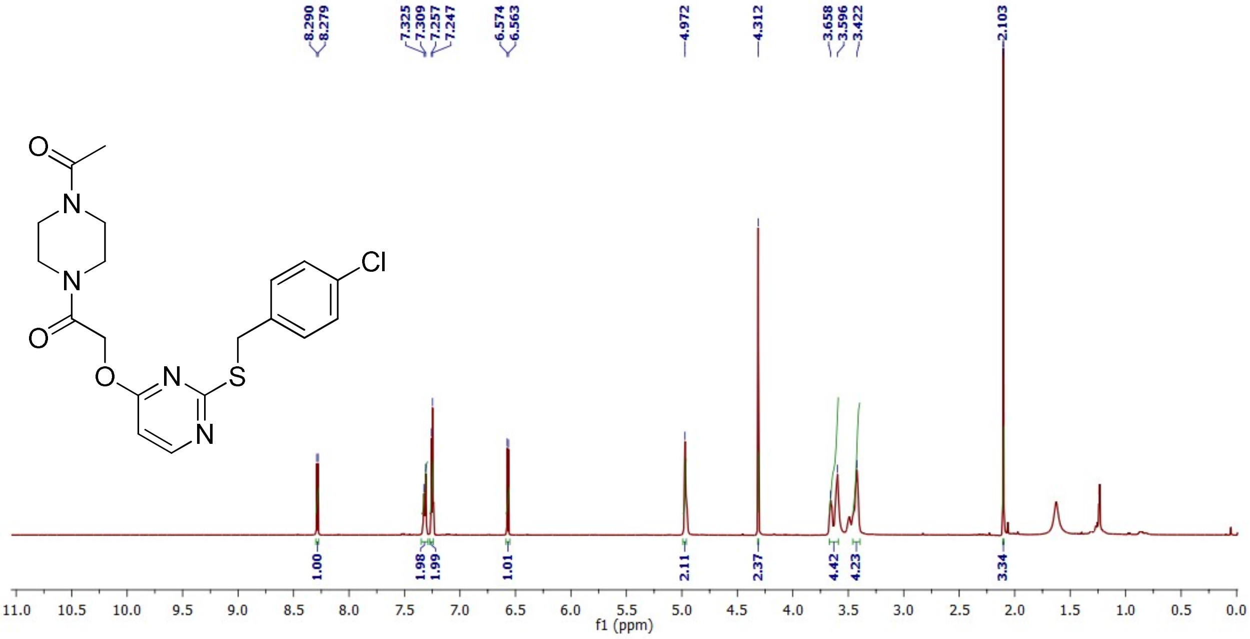

^{1}H NMR of 5**n**

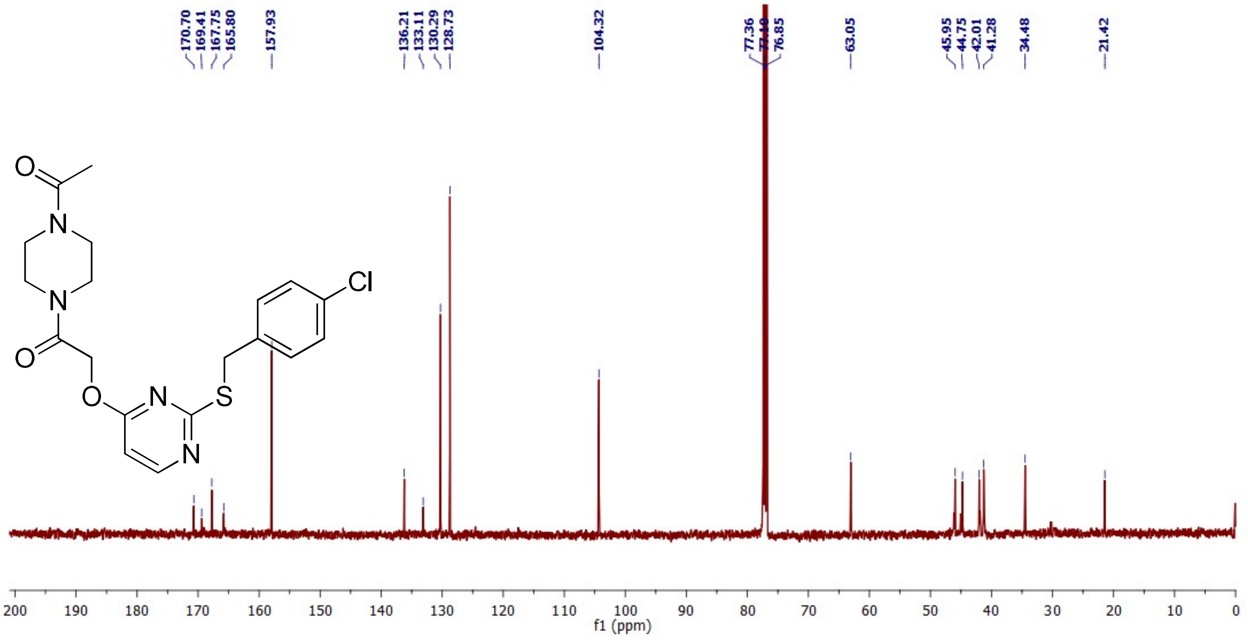

^{13}C NMR of 5**n**

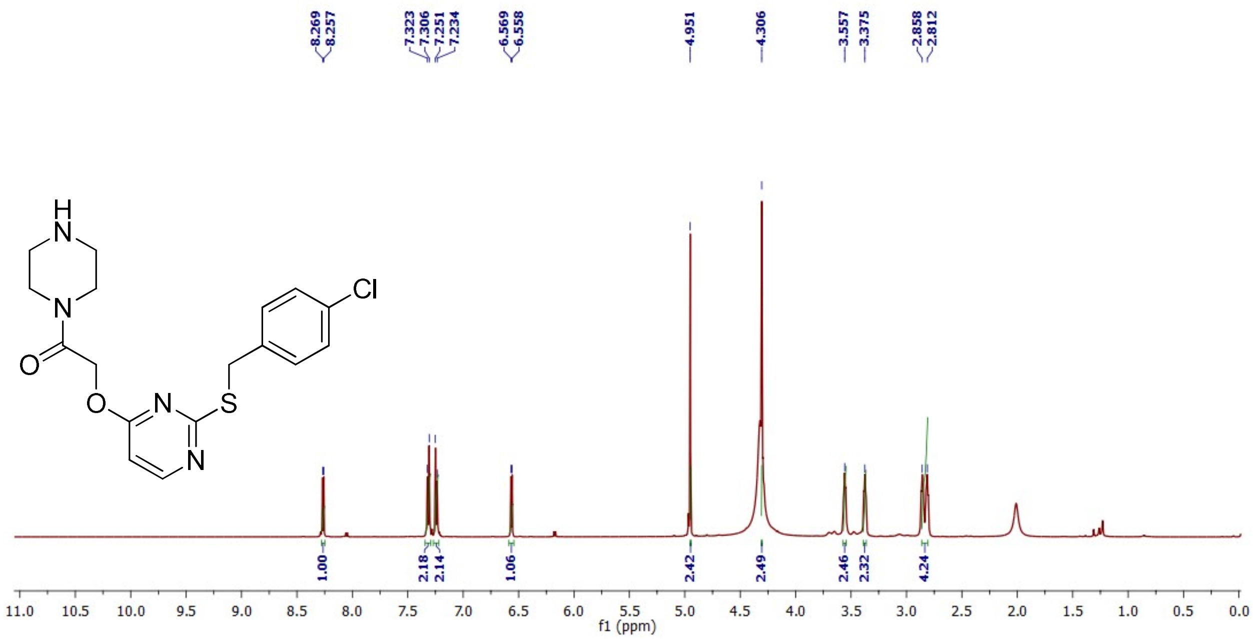

^{1}H NMR of 5**o**

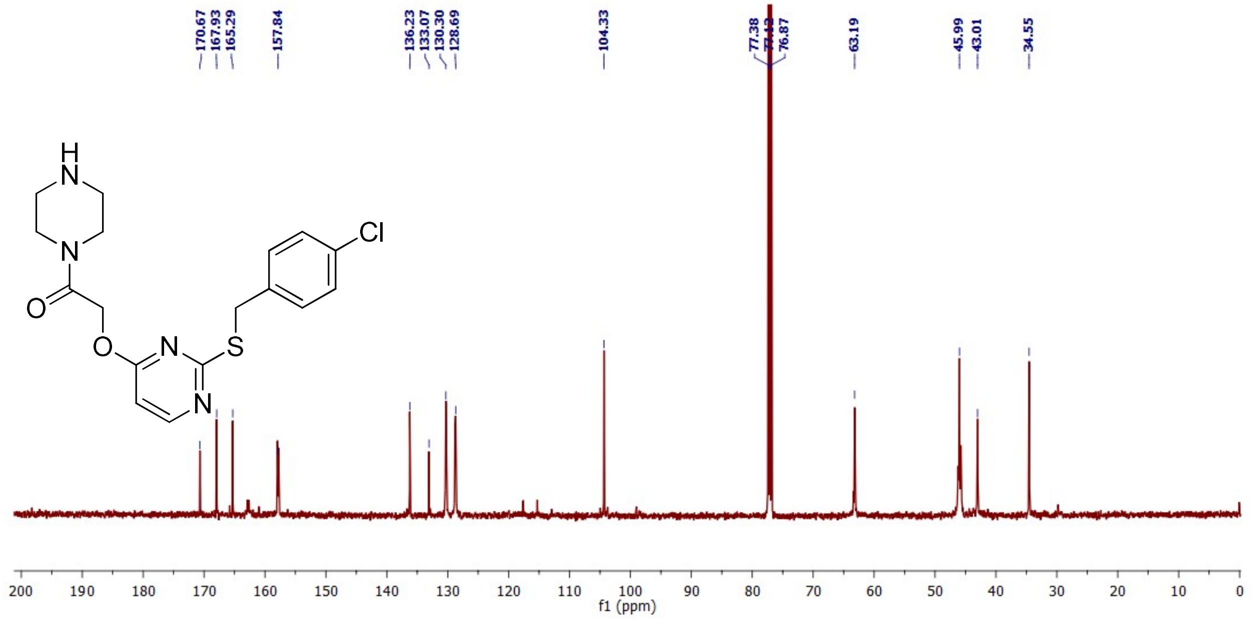

^{13}C NMR of 5**o**

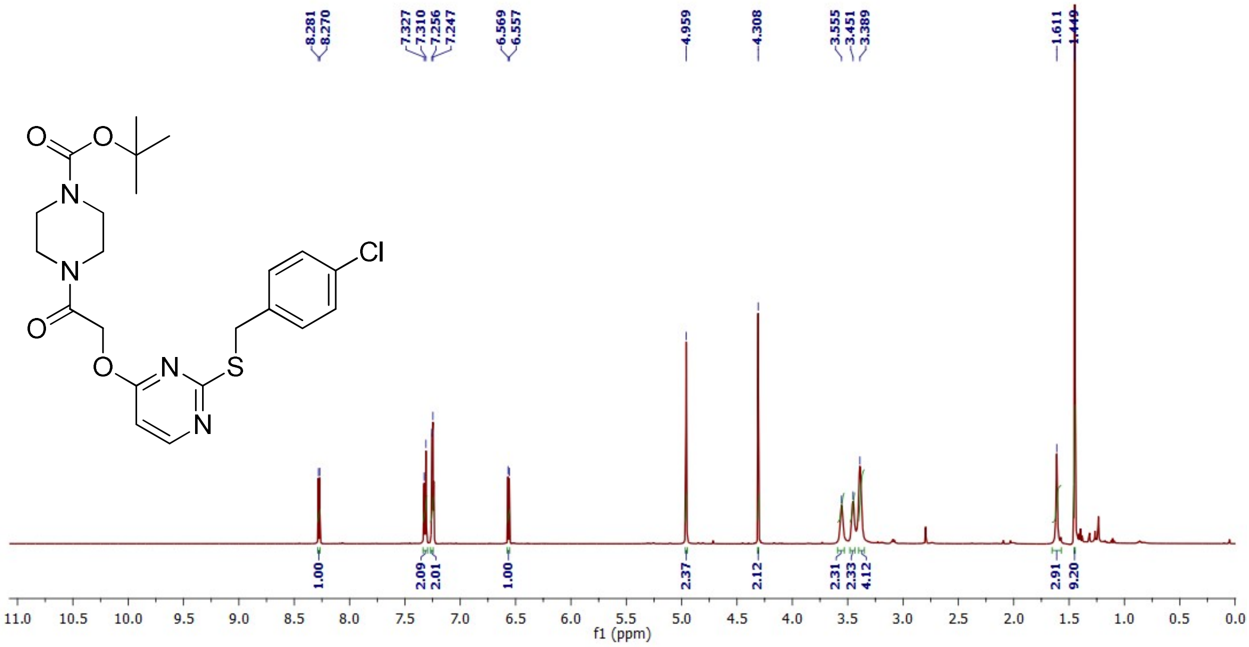

^{1}H NMR of 5**p**

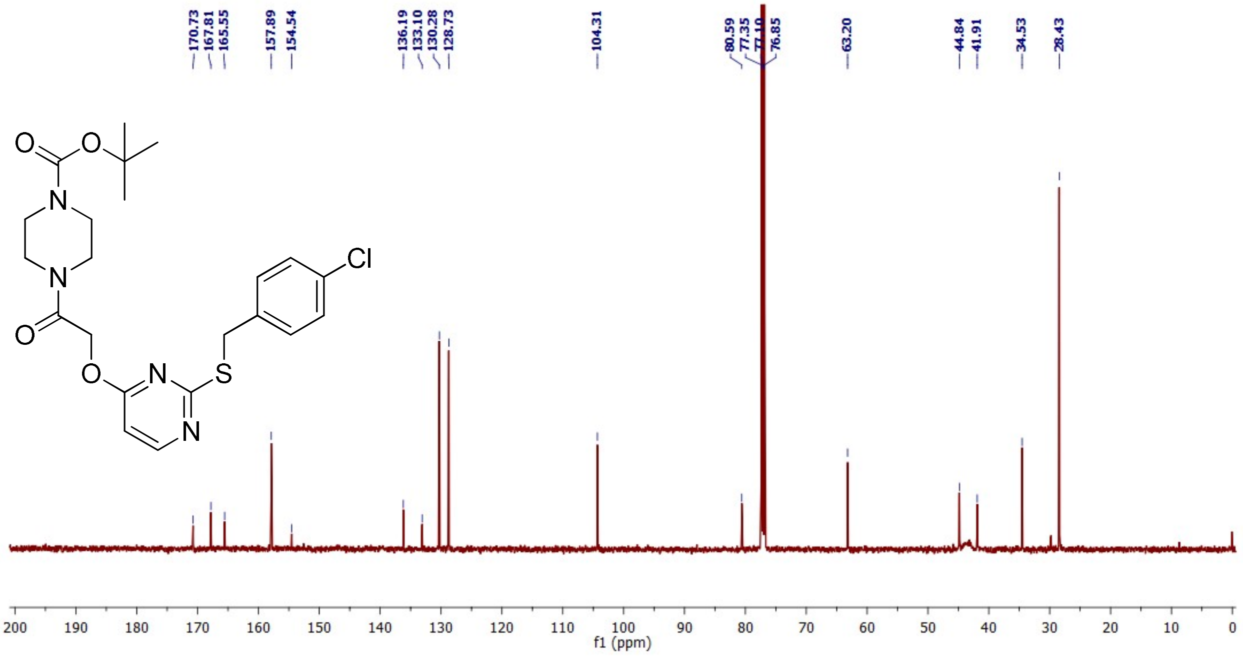

^{13}C NMR of 5**p**

IC_{50} values of molecules **3(a-j)** & **5(a-p)** against MCF-7 cells

- Cell line: MCF7(2000cells/per well 96-well plate)
- Treated time: 72hrs
- Assay: alamarBlue(4hrs incubated) N=3

3a

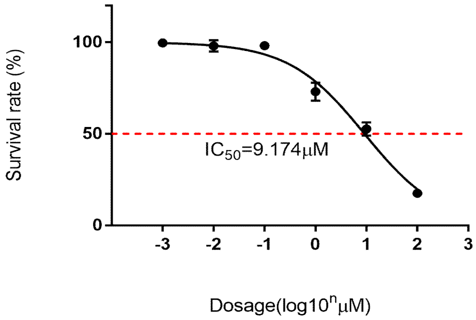

Conc.(μM)	Viability	
	AVE.	±SD
0	100	0.27
0.01	98.06	3.13
0.1	98.11	2.05
1	73.01	4.97
10	52.68	3.62
100	17.52	1.29

3b

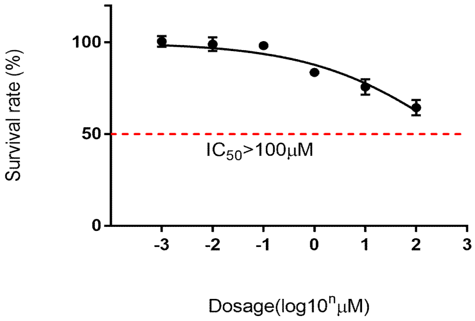

Conc.(μM)	Viability	
	AVE.	±SD
0	100.00	2.93
0.01	99.07	3.75
0.1	98.28	2.14
1	83.64	1.74
10	75.75	4.18
100	64.42	4.23

3c

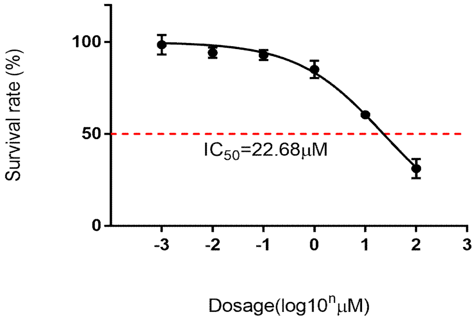

Conc.(μM)	Viability	
	AVE.	±SD
0	100.00	5.34
0.01	94.21	2.83
0.1	92.98	2.71
1	85.10	4.75
10	60.48	0.85
100	31.20	5.23

- Cell line: MCF7(2000cells/per well 96-well plate)
- Treated time: 72hrs
- Assay: alamarBlue(4hrs incubated) N=3

3d

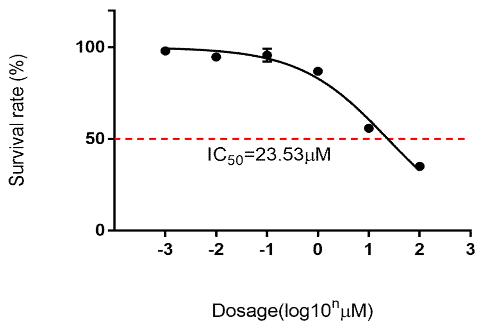

Conc.(μM)	Viability	
	AVE.	±SD
0	100.00	0.76
0.01	94.73	1.24
0.1	95.79	3.50
1	86.90	1.67
10	55.82	0.61
100	35.09	0.30

3e

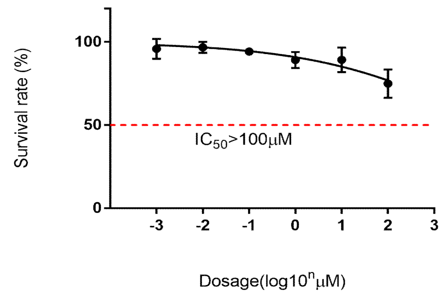

Conc.(μM)	Viability	
	AVE.	±SD
0	100.00	5.97
0.01	96.79	3.36
0.1	94.21	1.42
1	89.20	4.87
10	89.28	7.38
100	74.97	8.48

3f

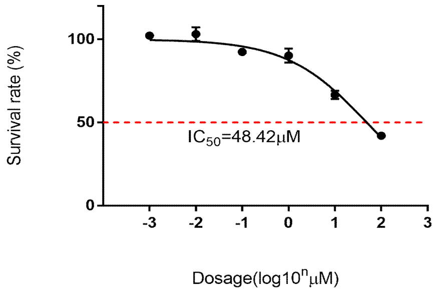

Conc.(μM)	Viability	
	AVE.	±SD
0	100.00	1.36
0.01	103.18	4.10
0.1	92.45	1.15
1	90.23	4.19
10	66.65	2.52
100	42.01	0.80

- Cell line: MCF7(2000cells/per well96-well plate)
- Treated time: 72hrs
- Assay: alamarBlue(4hrs incubated) N=3

3g

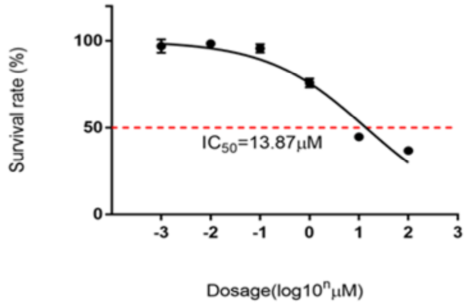

Conc.(μM)	Viability	
	AVE.	±SD
0	100.00	3.81
0.01	98.47	1.22
0.1	95.72	2.40
1	75.82	2.54
10	44.77	2.26
100	36.87	2.04

3h

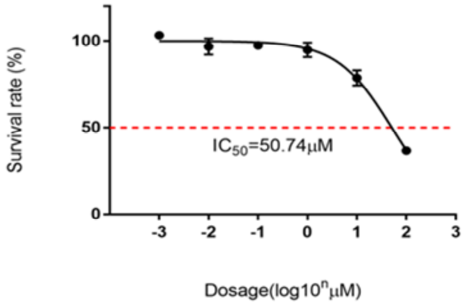

Conc.(μM)	Viability	
	AVE.	±SD
0	100.00	2.21
0.01	96.84	4.58
0.1	97.52	1.24
1	94.95	4.06
10	78.76	4.46
100	36.73	1.30

3j

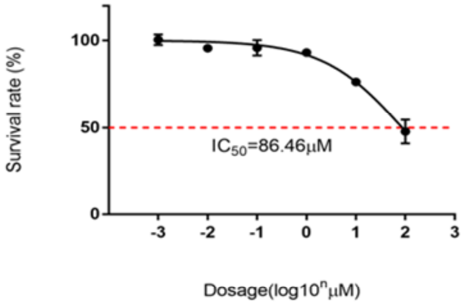

Conc.(μM)	Viability	
	AVE.	±SD
0	100.00	3.03
0.01	95.66	1.14
0.1	95.82	4.39
1	93.15	1.66
10	76.31	0.98
100	47.83	6.86

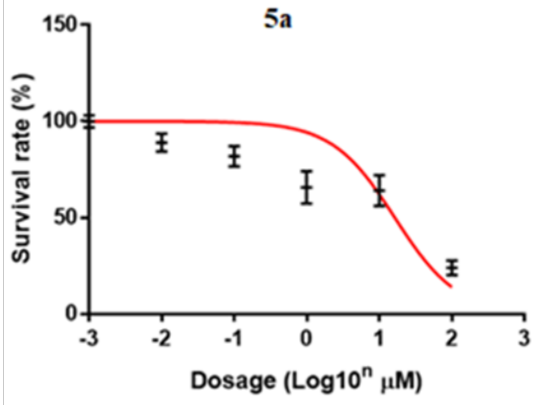

IC_{50} (μM) = 16.38
Log IC_{50} (μM) = 1.21

- Cell line: MCF7 (2000 cells/per well[96])
- Treated time: 72hrs
- Assay: MTT (90mins incubated)

Conc. (μM)	Viability	
	AVE.	± SD.
0	100.00	3.24
0.01	88.99	4.69
0.1	81.77	5.28
1	65.64	8.43
10	64.11	8.07
100	23.90	3.79

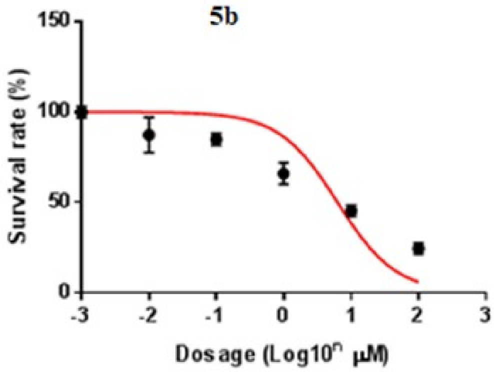

IC_{50} (μM) = 6.29
Log IC_{50} (μM) = 0.80

- Cell line: MCF7 (2000 cells/per well[96])
- Treated time: 72hrs
- Assay: MTT (90mins incubated)

Conc. (μM)	Viability	
	AVE.	± SD.
0	100.00	3.24
0.01	87.34	9.78
0.1	84.81	3.29
1	65.95	6.08
10	45.36	3.18
100	24.47	3.01

- Cell line: MCF7 (2000 cells/per well[96])
- Treated time: 72hrs
- Assay: MTT (90mins incubated)

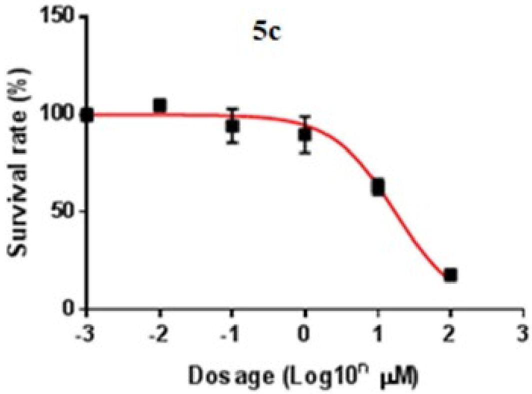

IC$_{50}$ (μM) = 17.26
Log IC$_{50}$ (μM) = 1.24

Conc. (μM)	Viability	
	AVE.	± SD.
0	100.00	3.24
0.01	104.74	2.95
0.1	94.25	8.87
1	89.71	9.39
10	62.88	4.10
100	17.74	0.85

- Cell line: MCF7 (2000 cells/per well[96])
- Treated time: 72hrs
- Assay: MTT (90mins incubated)

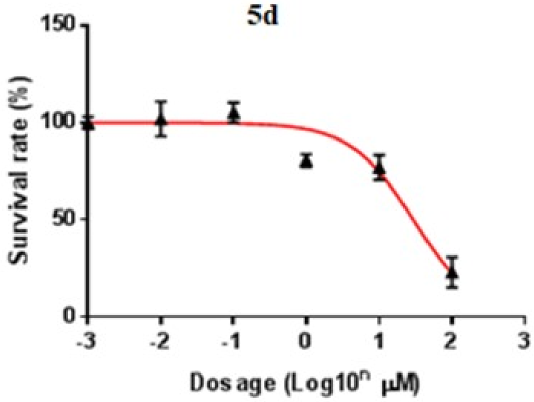

IC$_{50}$ (μM) = 29.38
Log IC$_{50}$ (μM) = 1.47

Conc. (μM)	Viability	
	AVE.	± SD.
0	100.00	3.24
0.01	101.90	9.09
0.1	105.19	5.08
1	80.57	3.12
10	76.95	6.40
100	23.00	7.76

- Cell line: MCF7(2000cells/per well 96-well plate)
- Treated time: 72hrs
- Assay: alamarBlue(4hrs incubated) N=3

5e

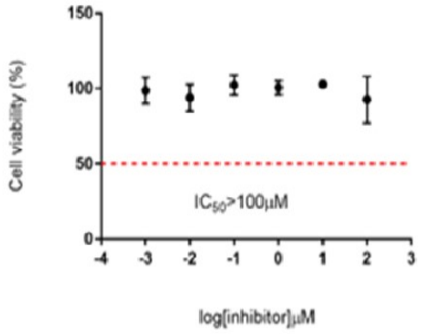

Conc.(μM)	Viability (%)	
	AVE.	±SD
0	100.0	8.7
0.01	94.1	8.9
0.1	102.5	6.5
1	100.7	4.6
10	103.0	2.3
100	92.9	15.5

5f

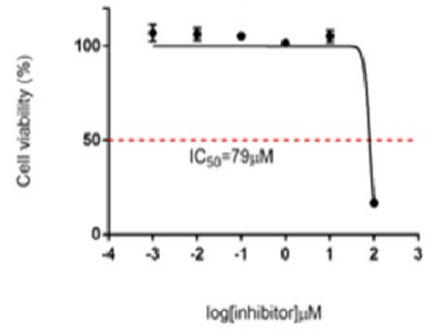

Conc.(μM)	Viability (%)	
	AVE.	±SD
0	100.0	4.3
0.01	106.3	3.3
0.1	105.4	2.3
1	101.6	2.3
10	105.2	3.2
100	16.7	2.0

5g

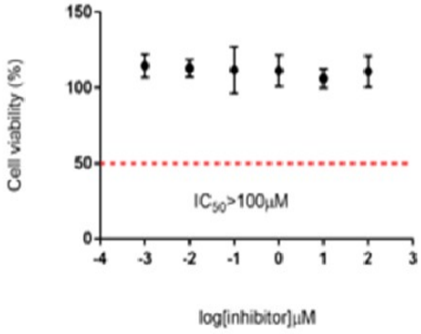

Conc.(μM)	Viability (%)	
	AVE.	±SD
0	100.0	7.7
0.01	113.0	5.8
0.1	111.8	15.3
1	111.4	10.4
10	106.2	6.4
100	110.8	10.4

- Cell line: MCF7 (2000 cells/per well[88])
- Treated time: 72hrs
- Assay: MTT (4 hrs incubated)

5h

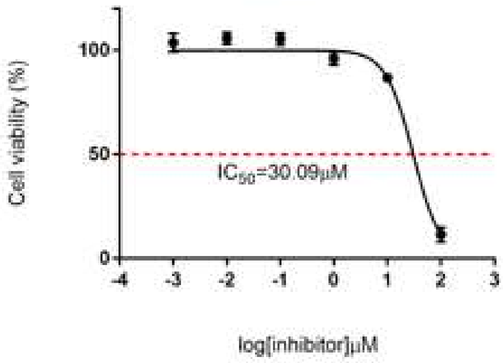

Conc.(μM)	Viability (%)	
	AVE.	±SD
0	100.0	4.4
0.01	105.8	2.9
0.1	105.4	2.9
1	96.2	3.1
10	86.8	0.8
100	11.4	3.6

5i

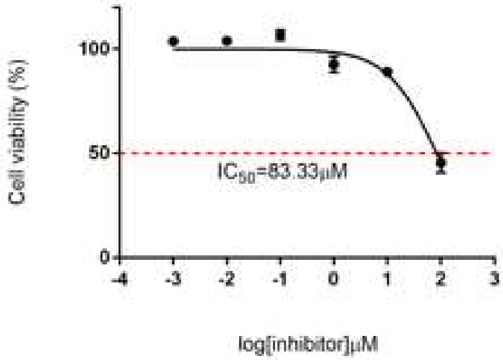

Conc.(μM)	Viability (%)	
	AVE.	±SD
0	100.0	1.4
0.01	104.0	1.2
0.1	106.9	2.4
1	92.7	3.8
10	89.2	2.2
100	45.5	4.8

5j

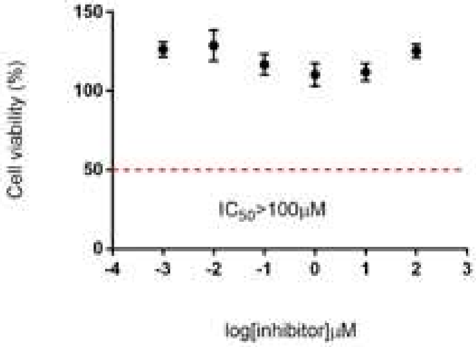

Conc.(μM)	Viability (%)	
	AVE.	±SD
0	100.0	4.8
0.01	128.8	9.7
0.1	116.8	6.7
1	110.3	7.3
10	111.9	5.6
100	125.3	4.3

- Cell line: MCF7 (2000 cells/per well[96])
- Treated time: 72hrs
- Assay: MTT (4 hrs incubated)

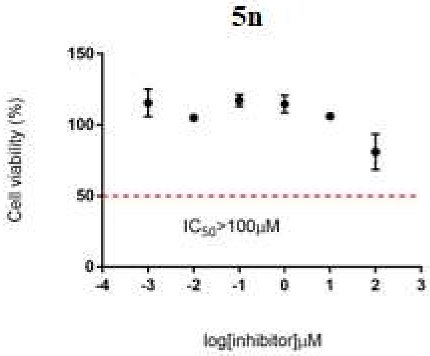

Conc.(μM)	Viability (%)	
	AVE.	±SD
0	100.0	9.6
0.01	105.0	0.5
0.1	117.3	4.1
1	114.7	6.1
10	106.2	2.9
100	81.1	12.7

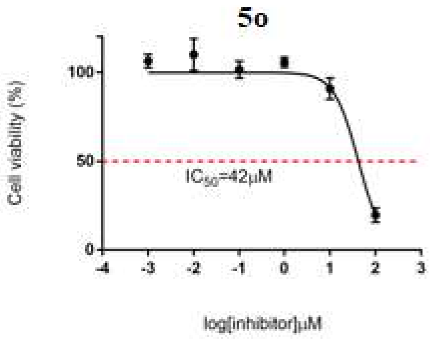

Conc.(μM)	Viability (%)	
	AVE.	±SD
0	100.0	3.7
0.01	110.1	8.9
0.1	101.6	4.8
1	105.7	2.8
10	90.8	6.1
100	19.7	4.0

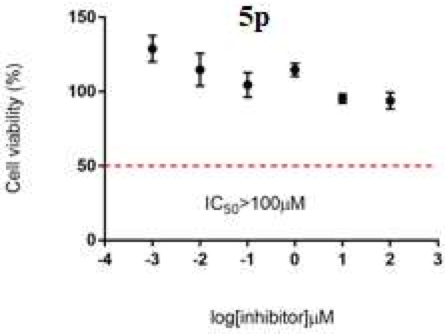

Conc.(μM)	Viability (%)	
	AVE.	±SD
0	100.0	8.8
0.01	114.8	10.9
0.1	104.6	8.3
1	114.7	4.3
10	95.4	3.0
100	93.9	5.5

IC_{50} of **3a** against MDA-MB-231, BT-549, SUM159PT

- Cell line: TNBC :MDAMB231;BT-549;SUM159PT (2000cells/per well96-well plate)
- Treated time: 72hrs
- Assay: alamarBlue (4hrs incubated)

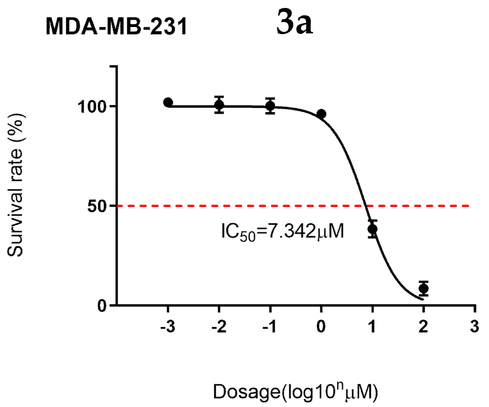

Conc.(μM)	Viability	
	AVE.	±SD
0	100.0	1.1
0.01	100.8	4.1
0.1	100.3	3.8
1	96.3	1.1
10	38.4	4.2
100	8.6	3.4

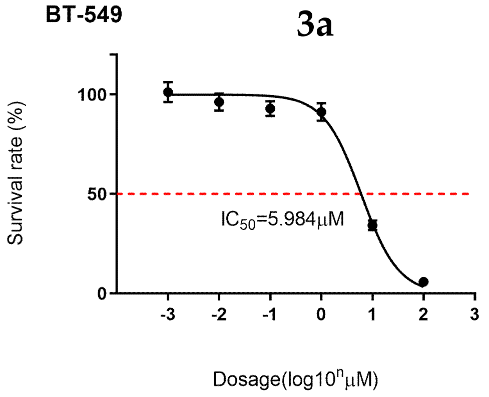

Conc.(μM)	Viability	
	AVE.	±SD
0	100.0	5.0
0.01	96.3	4.3
0.1	92.9	3.7
1	91.3	4.4
10	34.2	2.5
100	5.8	2.2

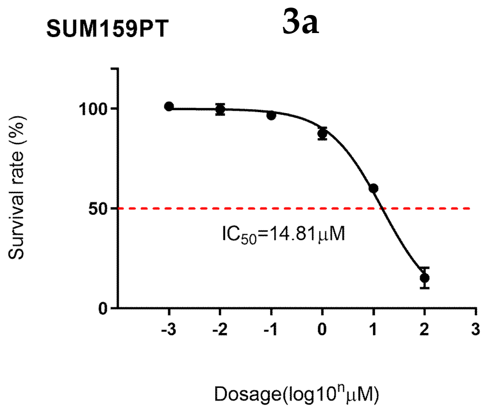

Conc.(μM)	Viability	
	AVE.	±SD
0	100.0	1.6
0.01	99.7	2.6
0.1	96.8	1.4
1	87.6	2.9
10	60.2	2.0
100	15.2	5.1

IC$_{50}$ of **5b** against MDA-MB-231, BT-549, SUM159PT

- ➢Cell line: TNBC :MDAMB231;BT-549;SUM159PT (2000cells/per well96-well plate)
- ➢Treated time: 72hrs
- ➢Assay: alamarBlue (4hrs incubated)

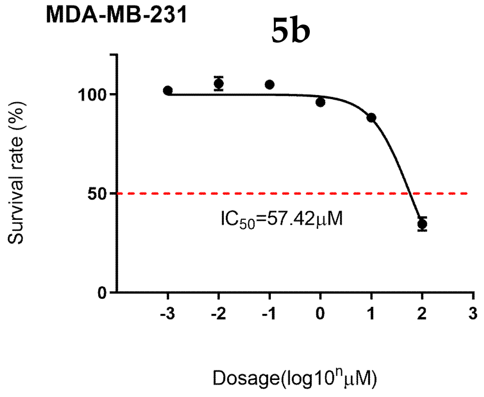

Conc.(μM)	Viability	
	AVE.	±SD
0	100.0	1.8
0.01	105.6	3.3
0.1	105.1	1.3
1	96.1	0.7
10	88.3	1.2
100	34.7	3.3

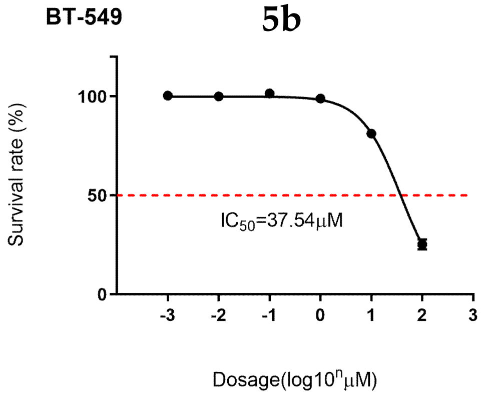

Conc.(μM)	Viability	
	AVE.	±SD
0	100.0	0.4
0.01	100.0	1.0
0.1	101.6	1.7
1	98.9	1.2
10	81.2	1.6
100		2.6

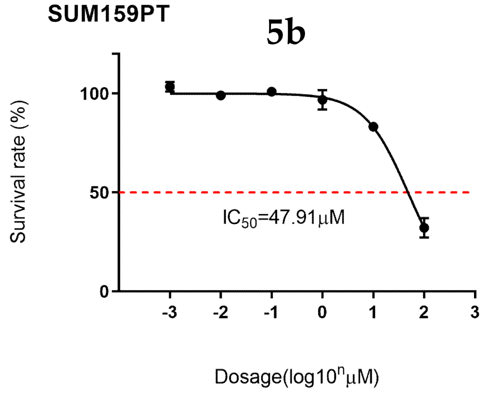

Conc.(μM)	Viability	
	AVE.	±SD
0	100.0	2.5
0.01	99.0	2.2
0.1	101.0	1.4
1	96.9	4.9
10	83.2	2.4
100	32.2	4.9

- Cell line: MCF7 (100000cells/per 6cm dish)
- Treated time: 24hrs
- Assay: Western blot
- Antibody:

Phospho-NFKBp65(S536)	Cell Signaling 30333S
NFKBp65	Cell Signaling 8242S
β-ACTIN	Santa Cruzesc-47778

Protein Ladder: 4A biotech MPM9207

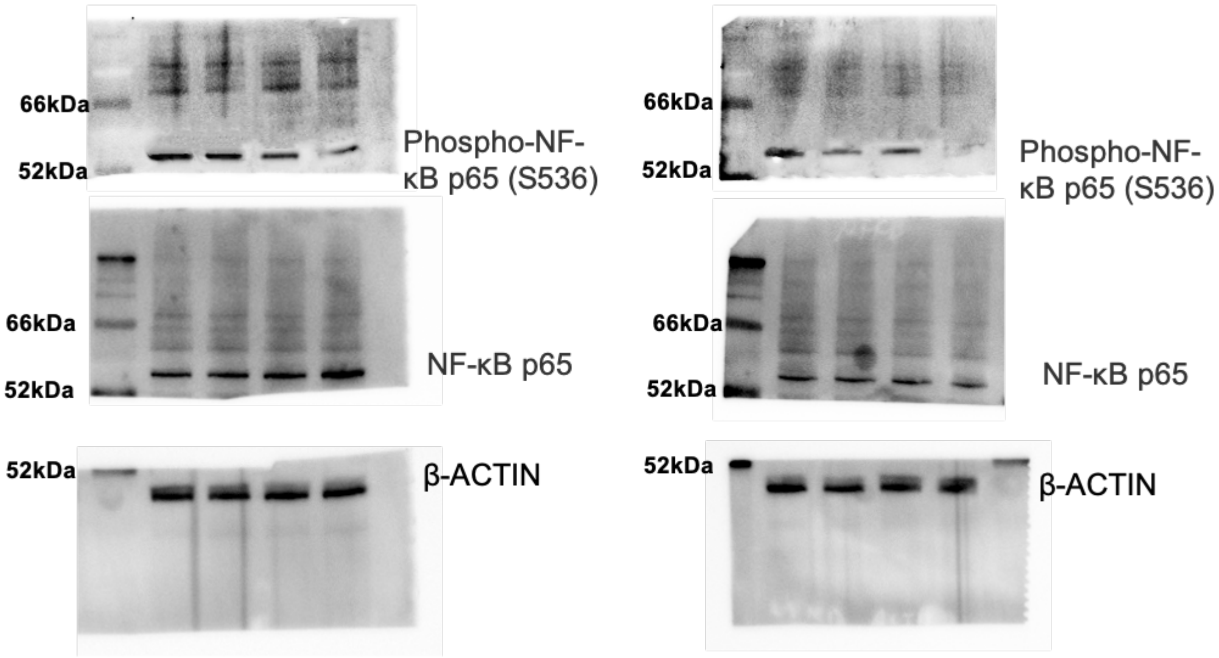

Supplement: Supplementary file 1 [file biomedicines-11-02716-s001.zip › biomedicines-2500216-supplementary.pdf]
